# Site-to-site peptide transport on a molecular platform using a small-molecule robotic arm†

**DOI:** 10.1039/d0sc05906d

**Published:** 2020-12-10

**Authors:** Salma Kassem, Alan T. L. Lee, David A. Leigh, Augustinas Markevicius, Daniel J. Tetlow, Naoyuki Toriumi

**Affiliations:** Department of Chemistry, University of Manchester Oxford Road Manchester M13 9PL UK david.leigh@manchester.ac.uk

## Abstract

Peptides attached to a cysteine hydrazide ‘transporter module’ are transported selectively in either direction between two chemically similar sites on a molecular platform, enabled by the discovery of new operating methods for a molecular transporter that functions through ratcheting. Substrate repositioning is achieved using a small-molecule robotic arm controlled by a protonation-mediated rotary switch and attachment/release dynamic covalent chemistry. A polar solvent mixtures were found to favour *Z* to *E* isomerization of the doubly-protonated switch, transporting cargo in one direction (arbitrarily defined as ‘forward’) in up to 85% yield, while polar solvent mixtures were unexpectedly found to favour *E* to *Z* isomerization enabling transport in the reverse (‘backward’) direction in >98% yield. Transport of the substrates proceeded in a matter of hours (compared to 6 days even for simple cargoes with the original system) without the peptides at any time dissociating from the machine nor exchanging with others in the bulk. Under the new operating conditions, key intermediates of the switch are sufficiently stabilized within the macrocycle formed between switch, arm, substrate and platform that they can be identified and structurally characterized by ^1^H NMR. The size of the peptide cargo has no significant effect on the rate or efficiency of transport in either direction. The new operating conditions allow detailed physical organic chemistry of the ratcheted transport mechanism to be uncovered, improve efficiency, and enable the transport of more complex cargoes than was previously possible.

## Abstract 2

Peptides are transported in either direction between chemically similar sites on a molecular platform, substrate repositioning is achieved using a cysteine hydrazide transporter module and a small-molecule robotic arm controlled by a rotary switch.

## Graphical abstract

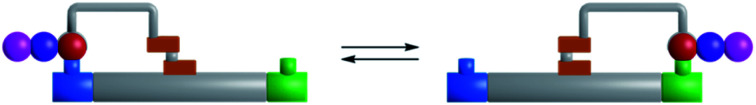

## Introduction

The manipulation of matter with molecular machines has intrigued scientists since it was proposed by Feynman over 60 years ago.^1^ The capability of one molecule to mechanically position another has some precedence in biology: fatty acid synthase^2^ and polyketide synthase^3^ both pass tethered molecules between domains located in different parts of enzyme superstructures. In doing so the substrate is prevented from exchanging with others in the bulk enabling the controlled synthesis of complex natural products.^4–6^ Such physical manipulation is reminiscent of the way robots position and process parts on factory assembly lines. A key feature of assembly line robots, shared by some biological molecular machines, is the ability to deliver different outcomes in response to different programed commands (for example, to build different protein sequences in the case of the ribosome).

A step towards the long-term vision of using small-molecule robots to perform useful tasks by passing building blocks and other cargoes between machines and along polymer chains and across surfaces, is to develop molecules that can pick up, reposition and put down substrates at specific sites. Programable DNA devices^7–16^ include examples that combine building blocks in a defined sequence^11–15^ or move gold nanoparticles.^16^ Small-molecule artificial molecular machines^17–23^ have been developed that can transport macroscopic droplets along a surface^24^ and perform other mechanical tasks,^25–29^ and that can carry out sequence-specific^30–33^ and programable^34–40^ chemical synthesis. Here we demonstrate that a molecular ‘robotic arm’^40–42^ can be programed to transport cysteine-containing peptide derivatives, rapidly and with good efficiency in either direction, between two chemically similar sites 2 nm apart on a molecular platform.

## Molecular robotic arm and platform design

A programable artificial molecular machine has been developed that is able to pick up, reposition, and set down 3-mercaptopropanehydrazide at either one of two different sites through judicious control of a robotic arm and ‘gripper’.^41^ The gripper picks up/releases the cargo by formation/breakage of a disulfide bond, while dynamic hydrazone chemistry modulates binding of the cargo to the platform.^43^ Transport is achieved by selectively inducing configurational (*E*-to-*Z* rotation about a C=N double bond) and conformational (rotation about a C–N single bond) changes of an embedded bidirectional hydrazone-based rotary switch^44,45^ that steers the arm. In a three-stage operation, 3-mercaptopropanehydrazide could be transported in either (chosen) direction between the two platform sites. The machine-induced transport is processive: the 3-mercaptopropanehydrazide does not at any time fully dissociate from the machine nor exchange with other molecules in the bulk, ensuring that substrate transport arises solely from the change in position of the robotic arm.

Limitations of these demonstrations included: (i) experiments were only carried out with a single, simple, molecular cargo; (ii) transport in one direction was slow, as the acid-catalyzed hydrazone exchange takes up to six days to equilibrate at the low concentrations of acid necessary for the mechanism used; and (iii) some steps in the transport process proceed in modest yield. We decided to investigate alternative operation mechanisms for the prototypical molecular robotic system while exploring its application for the site-to-site platform transport of more complex molecular substrates, in particular peptides.

## Results and discussion

*E*-to-*Z* (backward transport) experiments with molecular machine 1 were originally carried out using 5 equivalents of CF_3_CO_2_H.^41^ Selective protonation of the pyridine nitrogen of the hydrazone switch induced transport of 3-mercaptopropanehydrazide from the green site (attachment site 2; *E*-1) to the blue site (attachment site 1; *Z*-1). Although transport was achieved in good yield (91%), the process took six days to achieve this level of conversion. Consideration of the intermediates in the transportation pathway suggested the rate-limiting step to be acyl hydrazone exchange of the cargo between the two platform sites, presumably the rate limited by the low concentration of acid (5 equivalents) used. However, higher acid concentrations favour a different switch configuration, preventing transport in the desired direction.

In an effort to improve the efficacy of substrate transport by the molecular machine, we studied the effect of acid concentration on the process. We treated, independently, *E*-1 and *Z*-1 in C_2_D_2_Cl_4_ (2.5 mM) with increasing amounts of CF_3_CO_2_H (10–100 equivalents) and determined the *E*-1 : *Z*-1 ratio by ^1^H NMR spectroscopy once a steady state had been reached (see ESI, Section S3†).

High acid concentrations had a detrimental effect on *E*-1 to *Z*-1 conversion, decreasing from 90% *Z*-1 with 5 equivalents of CF_3_CO_2_H to 30% *Z*-1 with 70 equivalents. Increasing the concentration of CF_3_CO_2_H further did not vary the *E*-1 : *Z*-1 ratio from 70 : 30. Similarly, when starting from *Z*-1 the same steady state ratio of 70 : 30 *E*-1 : *Z*-1 was reached with 70 (or more) equivalents of CF_3_CO_2_H.

**Fig. 1:**
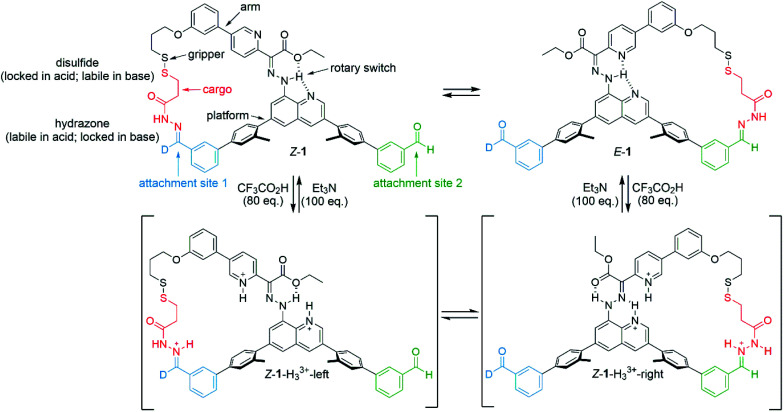
Operation of a platform-bound molecular robotic arm. Mechanism of exchange between *E*-1 and *Z*-1 using (i) CF_3_CO_2_H (80 equiv.), which causes protonation-induced configurational changes in the switch whilst simultaneously permiting hydrazide exchange at the platform sites, followed by (ii) Et_3_N (100 equiv.) which neutralizes the acid and switches off hydrazide exchange dynamics, locking the substrate in place on the platform. *Z*-1-H_3_^3+^-left and *Z*-1-H_3_^3+^-right are intermediates in the reaction mixture that could be identified and characterized by ^1^H NMR (see ESI†). Other conformations and hydrogen bond patterns may also be present.

These results are consistent with the equilibrium between *Z*-1-H_3_^3+^-right and *Z*-1-H_3_^3+^-left (Fig. 1; for identification and characterization of the intermediates, see ESI, Section S4.2†). The conversion between these intermediates occurs *via* rotation about the C–N (quinoline) bond of the transient acyl hydrazides. This type of conformational change was originally described by Aprahamian,^44^ in which a large excess of CF_3_CO_2_H leads to protonation of both the pyridine and quinoline nitrogens resulting in the doubly-protonated *Z*-isomer of the rotary switch (*Z*-1-H_3_^3+^-right; Fig. 1). The 70 : 30 *E* : *Z* ratio of the intermediates in C_2_D_2_Cl_4_ limits the extent of substrate transport possible. However, by using the results shown in Table 1 for C_2_D_2_Cl_4_ : C_6_D_5_CD_3_ (1 : 2), forward transport could be achieved with an improved 83% conversion by treating *Z*-1 with 80 equivalents of CF_3_CO_2_H to generate 83 : 17 *Z*-1-H_3_^3+^-right : *Z*-1-H_3_^3+^-left (Table 1; entry 10).^41^

**Table 1:** The position of equilibrium of 3-mercaptopropanehydrazide transporter *E*/*Z*-1 in various solvents, locked by the addition of Et_3_N to form different ratios of *E*-1 : *Z*-1^a^

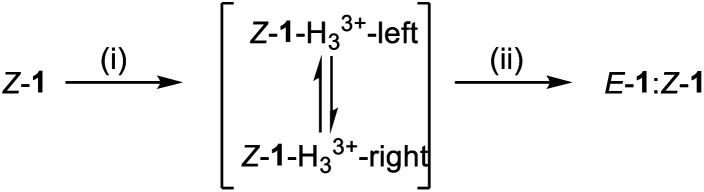			
Entry	Solvent	*E*-1 : *Z*-1	*ε* ^46^
1	C_2_D_2_Cl_4_ : (CD_3_)_2_SO (2 : 1)	>2 : 98	
2	CD_3_CN	>2 : 98	37.5
3	C_2_D_2_Cl_4_ : CD_3_CN (2 : 1)	>2 : 98	
4	C_2_D_2_Cl_4_ : CD_3_OD (2 : 1)	>2 : 98	
5	CD_2_Cl_2_	10 : 90	8.93
6	CF_3_CO_2_D	81 : 19	8.55
7	C_2_D_2_Cl_4_	70 : 30	8.42
8	CDCl_3_	75 : 25	4.81
9	C_2_D_2_Cl_4_ : C_6_D_5_CD_3_ (1 : 1)	81 : 19	4.15
10	C_2_D_2_Cl_4_ : C_6_D_5_CD_3_ (1 : 2)	83 : 17	3.59
11	C_2_D_2_Cl_4_ : C_6_D_5_CD_3_ (1 : 4)	85 : 15	3.11
12	CDCl_3_ : C_6_D_5_CD_3_ (1 : 1)	83 : 17	2.38
13	C_6_D_5_CD_3_	85 : 15	2.38

a Reagents and conditions: (i) CF_3_CO_2_H (80 equiv.), solvent (2.5 mM), r.t. (ii) Et_3_N (100 equiv.). Conversions determined by ^1^H NMR. *ε* is solvent polarity.^46,47^

### Exploring the unanticipated *E*/*Z* solvent effect: forward transport (*Z*-1 to *E*-1)

The conversion of *Z*-1 to *E*-1 was carried out in various solvents to fully explore the effect of the reaction medium on the equilibrium mixture. *Z*-1 was treated with 80 equivalents of CF_3_CO_2_H and the system allowed to reach a steady state. The *E*-1 : *Z*-1 ratios after neutralization and workup are shown in Table 1. In polar solvents and solvent mixtures containing (CD_3_)_2_SO, CD_3_CN and CD_3_OD, little or no conversion to *E*-1 was observed (Table 1, entries 1–4), indicating that the equilibrium favours *Z*-1-H_3_^3+^-left (hydrazone exchange is still dynamic under these reaction conditions). In CD_2_Cl_2_, 10% *E*-1 was formed (Table 1, entry 5). In less polar solvents such as C_2_D_2_Cl_4_ and CDCl_3_, a substantial increase in the amount of *E*-1 (corresponding to forward transport of the substrate) was observed (70% and 75%, respectively; Table 1, entries 7 and 8). Even lower polarity solvents, such as C_6_D_5_CD_3_, increased the amount of *E*-1 formed still further: 85% in 1 : 4 C_2_D_2_Cl_4_ : C_6_D_5_CD_3_ and neat C_6_D_5_CD_3_ (Table 1, entries 11 and 13).

There is a broad overall trend between solvent dielectric constant and the resulting steady state *E*-1 : *Z*-1 ratio (Table 1). Isomer *Z*-1-H_3_^3+^-left is favoured in high polarity solvents; *Z*-1-H_3_^3+^-right in low polarity media.^46–48^ It may be that polar, hydrogen-bond-disrupting, solvents solvate *Z*-1-H_3_^3+^-left more effectively than *Z*-1-H_3_^3+^-right, shifting the equilibrium position to favour the former despite unfavorable interactions between the hydrazone NH and the quinolinium NH proton. In apolar, non-hydrogen-bond-disrupting, solvents, *Z*-1-H_3_^3+^-right is favoured because of the more extensive intramolecular hydrogen bonding network. Even in neat CF_3_CO_2_D (Table 1, entry 6), a 81 : 19 *E*-1 : *Z*-1 ratio is produced broadly in line with the general trend regarding media polarity, despite the large excess of acid present.

### Backward transport (*E*-1 to *Z*-1)

We next turned our attention to improving *E*-1 to *Z*-1 conversion (backward transport of the substrate). We hypothesized that faster and higher conversions of *E*-1 to *Z*-1 might be possible using 80 equivalents of CF_3_CO_2_H in polar solvents that favour intermediate *Z*-1-H_3_^3+^-left. The molecular machine operation was followed by ^1^H NMR (see ESI Section S5†). Addition of 80 equivalents of CF_3_CO_2_H to *E*-1 in C_2_D_2_Cl_4_ : CD_3_CN (2 : 1) led to *Z*-1-H_3_^3+^-right, which converts to *Z*-1-H_3_^3+^-left (identified by ^1^H NMR) over an 8 hour period. The ^1^H NMR spectrum of the product after workup showed >98% conversion to *Z*-1. This is a significant improvement in speed and efficiency of operation (>98%, 8 h) compared to the previously reported system (91%, 6 days). The findings demonstrate that the direction of cargo transport can be controlled solely by adjusting the polarity of the solvent while keeping other factors (*e.g.* acid concentration) unchanged. Polar solvent mixtures favour *E*-1 to *Z*-1 conversion; apolar solvent mixtures favour *Z*-1 to *E*-1 conversion.

A significant aspect of the architecture of molecular machine 1 is that binding of the substrate simultaneously to both the gripper of the machine arm and to the platform, forms a macrocycle. The results in Fig. 1 show that incorporating the isomeric forms of the hydrazone rotary switch into such a macrocycle has a profound effect on the chemistry of the switch. It permits the observation of otherwise transient intermediates in the switching process, which was used to modify the machine operation protocols resulting in significantly improved transport efficiency and dynamics.

### Site-to-site transport of peptide derivatives

A potential useful feature of molecular machine 1 is that the structure of the 3-mercaptopropanehydrazide cargo can be elaborated upon without changing its interaction points with the platform and the arm. This could allow this unit to be used as a ‘transporter module’ for other small-molecules attached to it. 3-Mercaptopropanehydrazide is the hydrazide derivative of the amino acid cysteine, missing the amino group. Accordingly, we explored the site-to-site transport of a series of peptidic cysteine derivatives in both forward and backward directions to investigate whether the modified cargo would have an effect on the transportation process.

**Fig. 2:**
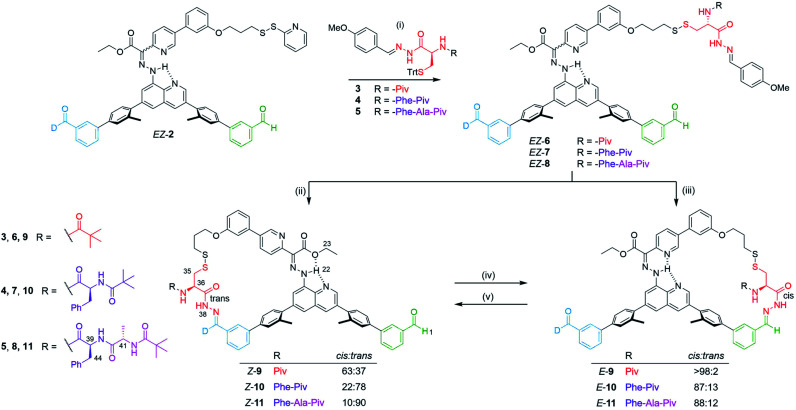
Synthesis and selective site-to-site transport of peptide derivatives in either direction by a small-molecule robotic arm. Reagents and conditions: (i) AgNO_3_ (1.3 equiv.), CHCl_3_ : MeOH (2 : 1), r.t., 30–60 min, then *E*/*Z*-2 (1.5 equiv.), CHCl_3_, r.t., 3–27 h, 63–79%. (ii) CF_3_CO_2_H (3.0 equiv.), CHCl_3_, r.t., 16 h, 38–64%. (iii) CF_3_CO_2_H (70 equiv.), CHCl_3_, r.t., 16–23 h, 22–79%. (iv) CF_3_CO_2_H (80 equiv.), C_2_D_2_Cl_4_ : C_6_D_5_CD_3_ (1 : 2), r.t., 2 h, *E*-9 66%, *E*-10 65%, *E*-11 63%. (v). CF_3_CO_2_H (80 equiv.), C_2_D_2_Cl_4_ : CD_3_CN (2 : 1), r.t., 6 h, *Z*-9 > 98%, *Z*-10 > 98%, *Z*-11 > 98%.

A series of peptides (3–5, Fig. 2, see ESI Sections 2.1,† 2.2 and 2.3 for synthesis and characterization) were prepared bearing a cysteine hydrazide at the C-terminus. The thiol was trityl-protected to facilitate loading of the substrate onto the molecular machine arm gripper *via* silver-mediated disulfide formation.^49,50^ Deprotection of the trityl group was achieved with silver nitrate, generating the silver thiolate intermediate which reacts with the 2-pyridyl disulfide moiety of *E*/*Z*-2 to afford *E*/*Z*-6–8 in 63–79% yield (Fig. 2, step (i)). The C-terminus acyl hydrazide was capped as the corresponding *p*-methoxyphenyl acyl hydrazone in order to facilitate cyclization onto the platform *via* hydrazone exchange. Subsequent macrocyclization to the corresponding *Z* and *E* macrocycles was promoted by CF_3_CO_2_H (3 and 70 equivalents respectively, Fig. 2, steps (ii) and (iii)).

**Fig. 3:**
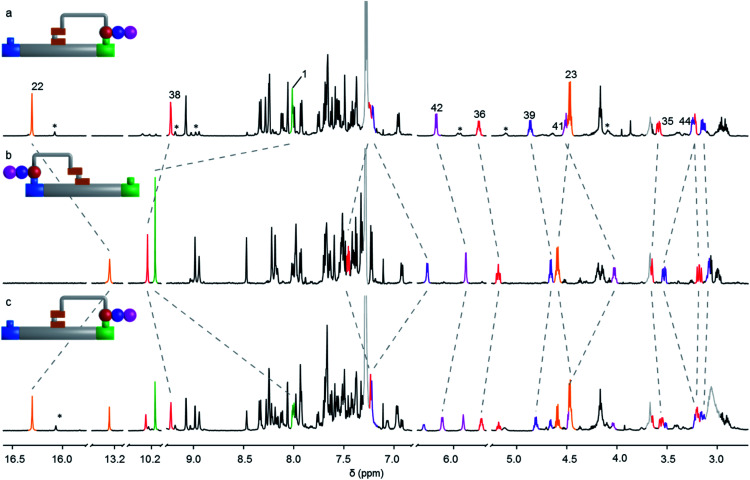
Partial ^1^H NMR (600 MHz, 295 K, CDCl_3_, 2.5 mM) spectra of the interconversion of *E*-11 and *Z*-11. (a) *E*-11 (tripeptide on the platform green site); (b) backward transport of the tripeptide from the green site to the blue site, *i.e.* conversion of *E*-11 to *Z*-11 (CF_3_CO_2_H, 80 equiv., C_2_D_2_Cl_4_ : CD_3_CN (2 : 1), >98%), (c) forward transport of the tripeptide from the blue site to the green site, *i.e.* conversion of *Z*-11 to *E*-11 (CF_3_CO_2_H, 80 equiv., C_2_D_2_Cl_4_ : C_6_D_5_CD_3_ (1 : 2), 63%). Proton assignments correspond to the labelling in Fig. 2. Signals marked with an asterisk (*) correspond to the minor rotamer of the acyl hydrazone. Signals due to traces of residual solvents and organic salts are shown in grey.

^1^H NMR spectroscopy (*e.g.*Fig. 3) shows that the machine-substrate-platform macrocycles exist as a mixture of acyl hydrazone rotamers (restricted rotation about the NH–CO bond of the cargo). Generally, acyl hydrazones prefer to adopt a *cis* conformation.^45^ However, substituents can destabilize the *cis* rotamer at the expense of the *trans* conformer.^51^ For the single amino acid derivative, *Z*-9 (R = Piv, Fig. 2), the *cis* conformer is favoured (63%), whereas with the tri-peptide, *Z*-11 (R = Phe-Ala-Piv), the *trans* isomer dominates (90%). The larger *E*-9–11 macrocycles strongly favour the *cis* rotamer of the acyl hydrazone irrespective of the size of the peptide cargo: the *trans* rotamer is not observed in *E*-9 and only a small amount is present in *E*-10 and *E*-11 (13 and 12%, respectively).

Site-to-site transport of the peptides was carried out in the backward direction (*E* to *Z*) using CF_3_CO_2_H (80 equiv.) in C_2_D_2_Cl_4_ : CD_3_CN (2 : 1). Conversion of *E*-9 to *Z*-9 (transport of a single amino acid) was achieved in >98% conversion in line with the results obtained with 3-mercaptopropanehydrazide (Table 1, entry 3). Pleasingly, the same behavior was observed in the backward transport of the di- and tri-peptides, both proceeding in >98% yield. The ^1^H NMR spectra of the operations to form *E*-11 and *Z*-11 are shown in Fig. 3 where successful right-to-left transport of the tripeptide (*E*-11 to *Z*-11) is evident from the characteristic shift of the imine proton H_1_ into the aldehyde region (from 7.94 ppm to 10.16 ppm). The upfield shift of the hydrazone NH proton H_22_ from 16.30 ppm to 13.26 ppm, and the downfield shift of the hydrazone ester group indicate *E* to *Z* isomerization of the rotary switch (Fig. 3a and b). Forward transport (*Z*-11 to *E*-11) was carried out with CF_3_CO_2_H (80 equiv.) in C_2_D_2_Cl_4_ : C_6_D_5_CD_3_ (1 : 2). Conversion of *Z*-11 to *E*-11 proceeded in 63% yield with the outcome of the operation analyzed by ^1^H NMR spectroscopy in a similar fashion (Fig. 3c). A decrease in forward transport compared to the 3-mercaptopropanehydrazide cargo (*Z*-1 to *E*-1, 83%) was found for *Z*-9, *Z*-10 and *Z*-11 (66%, 65% and 63% respectively). Nevertheless, successful site-to-site transport of the series of peptide-derived cargoes indicates that the size of the cargo does not prevent transport, nor does the size of the peptide affect the efficiency of transport.

## Conclusions

The new operating conditions allow detailed physical organic chemistry of the mechanism of a ratcheted molecular transporter to be uncovered, its efficiency improved, and the transport of more complex cargoes with an artificial molecular machine. The findings demonstrate that site-to-site transport of molecular substrates by a small-molecule robotic arm can proceed within hours in either (chosen) direction with >98% conversion in one direction and 63–85% conversion (depending on the substrate) in the other. The direction of transport can be reversed by changing the polarity of the medium rather than control of the protonation level of the switch. The new operation conditions allow key interconvertible intermediates involved in the process to be identified and characterized. Cysteine hydrazide can act as a ‘transporter module’ through which to attach and transport mono, di- and tripeptide cargoes between different sites on a molecular scaffold within reach of the robotic arm and gripper. The size of the peptide does not affect the effectiveness of programable transport in either direction. The ability to pass a substrate selectively in either direction between chemically similar sites through a ratchet mechanism, without the substrate ever being able to exchange with others in the bulk, is a rare feature for a synthetic molecular machine. The use of a cysteine transporter module for programable repositioning is a significant step towards the vision of complex molecular substrates being manipulated with atomic precision between multiple sites by robotic molecular machines.^22,40^

## Conflicts of interest

The authors declare no conflicts of interests.

## Supplementary Material

SC-012-D0SC05906D-s001

## References

[cit1] Feynman R. P. (1960). Eng. Sci..

[cit2] Maier T., Jenni S., Ban N. (2006). Science.

[cit3] Dutta S., Whicher J. R., Hansen D. A., Hale W. A., Chemler J. A., Congdon G. R., Narayan A. R. H., Håkansson K., Sherman D. H., Smith J. L., Skiniotis G. (2014). Nature.

[cit4] Maier T., Leibundgut M., Ban N. (2008). Science.

[cit5] Brignole E. J., Smith S., Asturias F. J. (2009). Nat. Struct. Mol. Biol..

[cit6] Chan D. I., Vogel H. J. (2010). Biochem. J..

[cit7] Ding B., Seeman N. C. (2006). Science.

[cit8] Gartner Z. J., Kanan M. W., Liu D. R. (2002). J. Am. Chem. Soc..

[cit9] Gartner Z. J., Tse B. N., Grubina R., Doyon J. B., Snyder T. M., Liu D. R. (2004). Science.

[cit10] Liao S., Seeman N. C. (2004). Science.

[cit11] He Y., Liu D. R. (2011). J. Am. Chem. Soc..

[cit12] McKee M. L., Milnes P. J., Bath J., Stulz E., O'Reilly R. K., Turberfield A. J. (2012). J. Am. Chem. Soc..

[cit13] McKee M. L., Milnes P. J., Bath J., Stulz E., Turberfield A. J., O'Reilly R. K. (2010). Angew. Chem., Int. Ed..

[cit14] Snyder T. M., Liu D. R. (2005). Angew. Chem., Int. Ed..

[cit15] Meng W., Muscat R. A., McKee M. L., Milnes P. J., El-Sagheer A. H., Bath J., Davis B. G., Brown T., O'Reilly R. K., Turberfield A. J. (2016). Nat. Chem..

[cit16] Gu H., Chao J., Xiao S.-J., Seeman N. C. (2010). Nature.

[cit17] Erbas-Cakmak S., Leigh D. A., McTernan C. T., Nussbaumer A. L. (2015). Chem. Rev..

[cit18] Abendroth J. M., Bushuyev O. S., Weiss P. S., Barrett C. J. (2015). ACS Nano.

[cit19] Sauvage J.-P. (2017). Angew. Chem., Int. Ed..

[cit20] Stoddart J. F. (2017). Angew. Chem., Int. Ed..

[cit21] Feringa B. L. (2017). Angew. Chem., Int. Ed..

[cit22] Zhang L., Marcos V., Leigh D. A. (2018). Proc. Natl. Acad. Sci. U. S. A..

[cit23] Heard A. W., Goldup S. M. (2019). ACS Cent. Sci..

[cit24] Berná J., Leigh D. A., Lubomska M., Mendoza S. M., Pérez E. M., Rudolf P., Teobaldi G., Zerbetto F. (2005). Nat. Mater..

[cit25] Huang T. J., Brough B., Ho C.-M., Liu Y., Flood A. H., Bonvallet P. A., Tseng H.-R., Stoddart J. F., Baller M., Magonov S. (2004). Appl. Phys. Lett..

[cit26] Eelkema R., Pollard M. M., Vicario J., Katsonis N., Ramon B. S., Bastiaansen C. W. M., Broer D. J., Feringa B. L. (2006). Nature.

[cit27] Li Q., Fuks G., Moulin E., Maaloum M., Rawiso M., Kulic I., Foy J. T., Giuseppone N. (2015). Nat. Nanotechnol..

[cit28] Chen S., Wang Y., Nie T., Bao C., Wang C., Xu T., Lin Q., Qu D.-H., Gong X., Yang Y., Zhu L., Tian H. (2018). J. Am. Chem. Soc..

[cit29] Saura-Sanmartin A., Martinez-Cuezva A., Bautista D., Marzari M. R. B., Martins M. A. P., Alajarin M., Berna J. (2020). J. Am. Chem. Soc..

[cit30] Lewandowski B., De Bo G., Ward J. W., Papmeyer M., Kuschel S., Aldegunde M. J., Gramlich P. M. E., Heckmann D., Goldup S. M., D'Souza D. M., Fernandes A. E., Leigh D. A. (2013). Science.

[cit31] De Bo G., Kuschel S., Leigh D. A., Lewandowski B., Papmeyer M., Ward J. W. (2014). J. Am. Chem. Soc..

[cit32] De Bo G., Gall M. A. Y., Kitching M. O., Kuschel S., Leigh D. A., Tetlow D. J., Ward J. W. (2017). J. Am. Chem. Soc..

[cit33] McTernan C. T., De Bo G., Leigh D. A. (2020). Chem.

[cit34] Beswick J., Blanco V., De Bo G., Leigh D. A., Lewandowska U., Lewandowski B., Mishiro K. (2015). Chem. Sci..

[cit35] Waelès P., Clavel C., Fournel-Marotte K., Coutrot F. (2015). Chem. Sci..

[cit36] Martinez-Cuezva A., Saura-Sanmartin A., Nicolas-Garcia T., Navarro C., Orenes R.-A., Alajarin M., Berna J. (2017). Chem. Sci..

[cit37] Riss-Yaw B., Clavel C., Laurent P., Waelès P., Coutrot F. (2018). Chem.–Eur. J..

[cit38] Martinez-Cuezva A., Marin-Luna M., Alonso D. A., Ros-Ñiguez D., Alajarin M., Berna J. (2019). Org. Lett..

[cit39] Dommaschk M., Echavarren J., Leigh D. A., Marcos V., Singleton T. A. (2019). Angew. Chem., Int. Ed..

[cit40] Kassem S., Lee A. T. L., Leigh D. A., Marcos V., Palmer L. I., Pisano S. (2017). Nature.

[cit41] Kassem S., Lee A. T. L., Leigh D. A., Markevicius A., Solà J. (2016). Nat. Chem..

[cit42] Chen J., Wezenberg S. J., Feringa B. L. (2016). Chem. Commun..

[cit43] von Delius M., Geertsema E. M., Leigh D. A. (2010). Nat. Chem..

[cit44] Su X., Aprahamian I. (2011). Org. Lett..

[cit45] Aprahamian I. (2017). Chem. Commun..

[cit46] Reichardt C. , Solvents and Solvent Effects in Organic Chemistry, Wiley-VCH Verlag GmbH & Co. KGaA, Weinheim, 3rd edn, 2003

[cit47] Nath J., Tripathi A. D. (1984). J. Chem. Soc., Faraday Trans. 1.

[cit48] Raamat E., Kaupmees K., Ovjannikov G., Trummal A., Kütt A., Saame J., Koppel I., Kaljurand I., Lipping L., Rodima T., Pihl V., Koppel I. A., Leito I. (2013). J. Phys. Org. Chem..

[cit49] Hamm M. L., Piccirilli J. A. (1997). J. Org. Chem..

[cit50] Hamm M. L., Piccirilli J. A. (1999). J. Org. Chem..

[cit51] Palla G., Predieri G., Domiano P., Vignali C., Turner W. (1986). Tetrahedron.

