# Supplementary material for: Site-to-site peptide transport on a molecular platform using a small-molecule robotic arm
Source: Chem Sci. 2020 Dec 10;12(6):2065–70. doi: 10.1039/d0sc05906d (PMC8179245; doi:10.1039/d0sc05906d)
Supplement: SC-012-D0SC05906D-s001 [file SC-012-D0SC05906D-s001.pdf]

Electronic Supplementary Material (ESI) for Chemical Science.
This journal is © The Royal Society of Chemistry 2020

Site-to-Site Peptide Transport on a Molecular Platform Using a Small-Molecule Robotic Arm

Salma Kassem, Alan T. L. Lee, David A. Leigh*, Augustinas Markevicius, Daniel J. Tetlow, and Naoyuki Toriumi

Department of Chemistry, University of Manchester, Oxford Road, Manchester M13 9PL, United Kingdom.

* E-mail: david.leigh@manchester.ac.uk

-Supporting Information-

Table of Contents

1. General Experimental……2
2. Experimental Procedures……3
2.1 Synthesis of *EZ*-**9**..……3
2.1.1 Synthetic Scheme……3
2.1.2 Synthetic Procedures and Characterisation Data……4
2.2 Synthesis of *EZ*-**10**……7
2.2.1 Synthetic Scheme……8
2.2.2 Synthetic Procedures and Characterisation Data……9
2.3 Synthesis of *EZ*-**11**……13
2.3.1 Synthetic Scheme……13
2.3.2 Synthetic Procedures and Characterisation Data……14
3. Effect of Acid Stoichiometry on *E*-**1**/*Z*-**1** Interconversion……20
3.1 Titration Procedure……20
3.2 Results……20
4. Effect of Solvent on *E*-**1**/Z-**1** Interconversion……20
4.1 Titration Procedure……20
4.2 Characterisation of *Z*-**1**-H_3^{3+}-Left and *Z*-**1**-H_3^{3+}-Right……21
4.2.1 *Z*-**1**-H_3^{3+}-Left in $C_2D_2Cl_4$:CD_3CN (2:1)……21
4.2.1 *Z*-**1**-H_3^{3+}-Right in $C_2D_2Cl_4$:$C_6D_5CD_3$ (1:2) ……23
5. Optimised Backward Transport (*E*-**1** to *Z*-**1**)……24
5.1 General Procedure……24
5.2 ^{1}H NMR Spectra……25
6. Site-to-Site Transport of Peptide Derivatives **9**, **10** and **11**.……26
6.1 Forward Transport……26
6.1.1 General Procedure……26
6.1.2 ^{1}H NMR Spectra……26
6.2 Backward Transport……28
6.2.1 General Procedure……28
6.2.2 ^{1}H NMR Spectra……29
7. Spectroscopic Data……32
8. References……53

1. General Experimental

Unless stated otherwise, reagents were obtained from commercial sources and used without purification. Reactions were carried out in anhydrous solvents and under an N_2 atmosphere. Anhydrous solvents were obtained by passing the solvent through an activated alumina column on a Phoenix SDS (solvent drying system; JC Meyer Solvent Systems, CA, USA). *EZ*-**1** and *EZ*-**2** were synthesized according to our previously reported procedure.[1] Pivaloylphenylalanine and Pivaloylalanine were prepared according to known literature procedures.[2] ^{1}H NMR spectra were recorded on a Bruker Avance III instrument with an Oxford AS600 magnet equipped with a cryoprobe [5mm CPDCH ^{13}C-^{1}H/D] (600 MHz). Chemical shifts are reported in parts per million (ppm) from high to low frequency using the residual solvent peak as the internal reference ($CDCl_3$ = 7.26 ppm, $C_2D_2Cl_4$ = 6.00 ppm, CD_2Cl_2 = 5.32 ppm, CD_3CN = 1.94 ppm, and $(CD_3)_2SO$ = 2.50 ppm).[3,4] All ^{1}H resonances are reported to the nearest 0.01 ppm. The multiplicity of ^{1}H signals are indicated as: s = singlet; d = doublet; t = triplet; q = quartet; quint = quintet; sept = septet; m = multiplet; br = broad; or combinations of thereof. Coupling constants (*J*) are quoted in Hz and reported to the nearest 0.1 Hz. Where appropriate, averages of the signals from peaks displaying multiplicity were used to calculate the value of the coupling constant. ^{13}C NMR spectra were recorded on the same spectrometer with the central resonance of the solvent peak as the internal reference ($CDCl_3$ = 77.16 ppm, $C_2D_2Cl_4$ = 73.00 ppm, CD_2Cl_2 = 54.00 ppm, CD_3CN = 118.26 ppm and $(CD_3)_2SO$ = 39.52 ppm).[3,4] All ^{13}C resonances are reported to the nearest 0.1 ppm, except in cases to aid the differentiation of closely resolved signals (which are reported to the nearest 0.01 ppm). DEPT, COSY, HSQC and HMBC experiments were used to aid structural determination and spectral assignment. Where necessary, 1D NOESY and 2D NOESY or ROESY spectra were used to aid the assignment of ^{1}H spectra. *Cis* and *trans* acyl hydrazone rotamers where assigned according to the characteristic ^{13}C shift of the amide carbon (with a lower field shift for the *cis* conformer). Fully characterized compounds were chromatographically homogeneous. Flash column chromatography was carried out using Silica 60 Å (particle size 40–63 µm, Sigma Aldrich, UK) as the stationary phase. Preparative TLC was performed using either PLC 20×20 cm, 60 F_{254} Preparative plates (Merck) or Silica Gel GF 20 × 20 cm, U_{254} Preparative plates (Analtech) of various thicknesses. TLC was performed on precoated silica gel plates (0.25 mm thick, 60 F_{254}, Merck, Germany) and visualized using both short and long wave ultraviolet light in combination with standard laboratory stains (basic potassium permanganate, acidic ammonium molybdate and ninhydrin). Low resolution ESI mass spectrometry was performed with a Thermo Scientific LCQ Fleet Ion Trap Mass Spectrometer or an Agilent Technologies 1200 LC system with either an Agilent 6130 single quadrupole MS detector or an Advion Expression LCMS single quadrupole MS detector. High-resolution mass spectrometry was carried out by the EPSRC National Mass Spectrometry Service Centre (Swansea, UK) or by staff at the Mass Spectrometry Service, School of Chemistry, The University of Manchester. Melting points (M.p.) were determined using a Büchi M-565 apparatus and are uncorrected.

2. Experimental Procedures

2.1 Synthesis of *EZ*-9

2.1.1 Synthetic Scheme

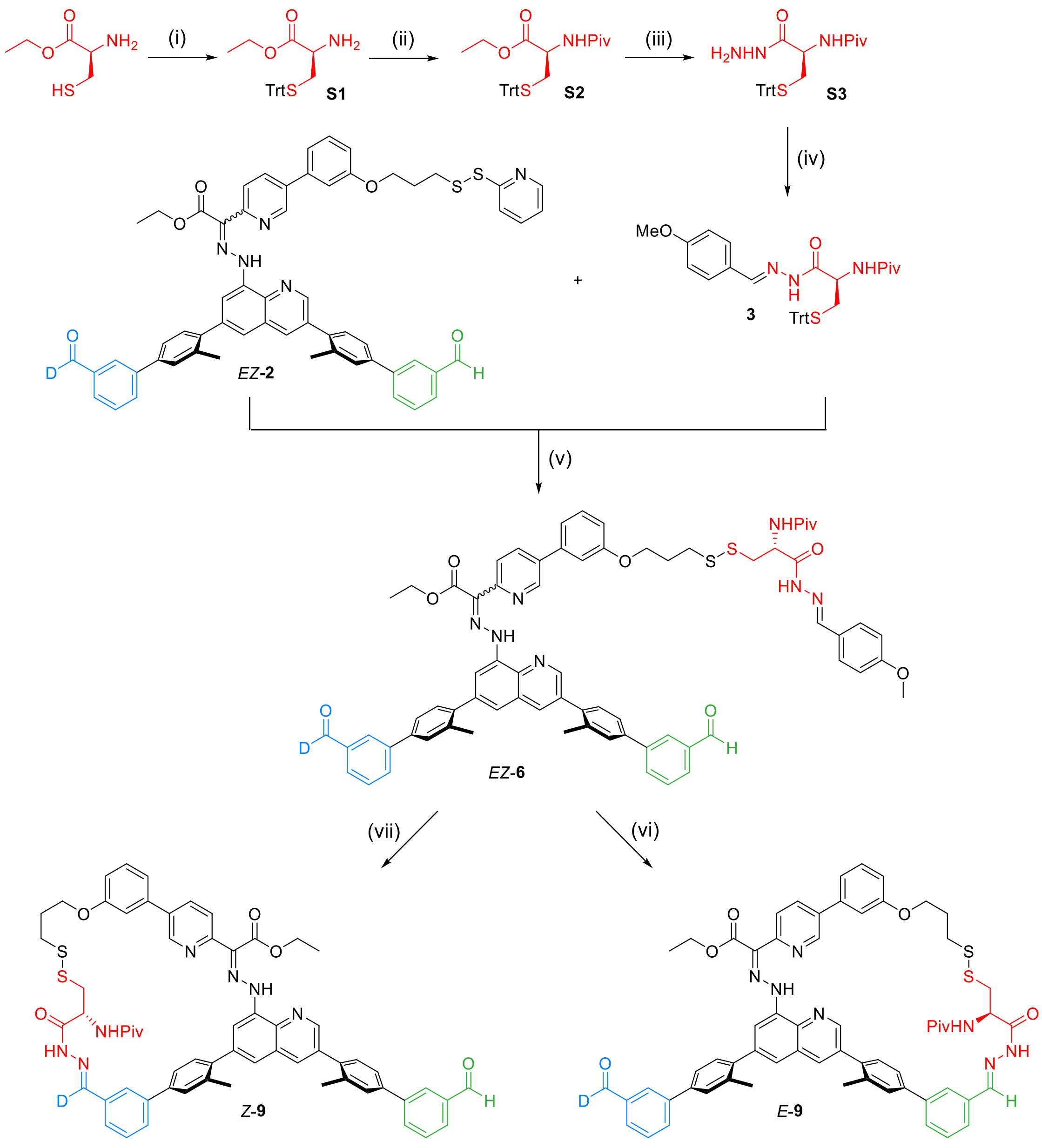

Scheme 1. Synthesis of *Z*-**9** and *E*-**9**. Reagents and conditions: (i) Ph_3COH, TFA, r.t., 75 min, 82%. (ii) PivCl, Et_3N, CH_2Cl_2, -10 °C to r.t., 1.5 h, 45%. (iii) $NH_2NH_2 \cdot H_2O$, MeOH, Δ, 67 h, 89%. (iv) *p*-anisaldehyde, AcOH, CH_2Cl_2, r.t., 24 h, 95%. (v) $AgNO_3$, $CHCl_3$, MeOH, r.t., 30 mins, then *EZ*-**2** in $CHCl_3$, r.t., 3 h, 79%. (vi) TFA, $CHCl_3$, r.t., 17 h, 53%. (vii) TFA, $CHCl_3$, r.t., 41 h, 64%.

2.1.2 Synthetic Procedures and Characterisation Data

Synthesis of **S1**

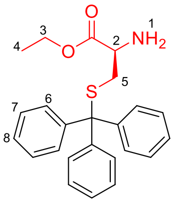

L-Cysteine ethyl ester hydrochloride (7.45 g, 40.1 mmol, 1.1 eq.) and triphenylmethanol (9.50 g, 36.5 mmol, 1 eq.) were dissolved in TFA (25 mL). The orange solution was stirred at room temperature for 75 minutes and the solvent was removed under reduced pressure. The resulting brown oil was dissolved in CH_2Cl_2 (200 mL) and water (200 mL) was added upon which the brown organics turned colourless. The mixture was neutralised by careful portion-wise addition of K_2CO_3 (*ca.* 15 g). The organic layer was separated, dried over $MgSO_4$ and concentrated under reduced pressure to a light purple oil. The crude oil was triturated with *n*-hexane which was then decanted to give the title compound as a yellow oil (12.9 g, 33.0 mmol, 82%). **^{1}H NMR** (600 MHz, DMSO-*d*$_6$) δ 7.37 – 7.29 (m, 12H, H_6+ H_7), 7.27 – 7.21 (m, 3H, H_8), 4.02 (q, *J* = 7.1 Hz, 2H, H_3), 3.14 (t, *J* = 6.4 Hz, 1H, H_2), 2.36 (dd, *J* = 11.9, 6.2 Hz, 1H, H_5), 2.28 (dd, *J* = 11.9, 6.7 Hz, 1H, $H_{5'}$), 1.82 (s, 2H, H_1), 1.13 (t, *J* = 7.1 Hz, 3H, H_4). **^{13}C NMR** (151 MHz, DMSO-*d*$_6$) δ 173.7, 144.4, 129.1, 128.0, 126.7, 65.9, 60.2, 53.7, 36.4, 14.1. **HRMS** (NSI$^+$): Calc. for $C_{24}H_{26}N_1O_2S$: 392.1679, found 392.1673 $[M+H]^+$. $[\alpha]_D^{20}$ +80.0 (*c* = 1.00, MeOH).

Synthesis of **S2**

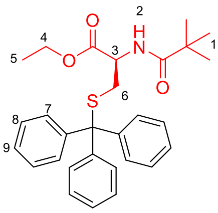

Pivaloyl chloride (130 µL, 1.06 mmol) was added dropwise to a solution of **S1** (338 mg, 0.862 mmol) and triethylamine (130 µL, 1.51 mmol) in CH_2Cl_2 (3 mL) at –10 °C under nitrogen atmosphere. The reaction mixture was warmed to room temperature and stirred for 1.5 hours. The reaction was quenched with H_2O and the layers separated. The aqueous layer was extracted with CH_2Cl_2 and the combined organic layers were dried over $MgSO_4$, filtered and concentrated under reduced pressure. Flash column chromatography (SiO_2, EtOAc/hexane, 20% → 70%) afforded **S2** as a colourless oil (184 mg, 0.387 mmol, 45%). **^{1}H NMR** (600 MHz, $CDCl_3$) δ 7.38 (d, *J* = 7.3 Hz, 6H, H_7), 7.28 (t, *J* = 7.3 Hz, 6H, H_8), 7.21 (t, *J* = 7.3 Hz, 3H, H_9), 6.29 (d, *J* = 7.6 Hz, 1H, H_2), 4.59 (dt, *J* = 7.6, 5.2 Hz, 1H, H_3), 4.17 (q, *J* = 7.1 Hz, 2H, H_4), 2.62 – 2.56 (m, 2H, H_6), 1.24 (t, *J* = 7.1 Hz, 3H, H_7), 1.21 (s, 9H, H_1). **^{13}C NMR** (151 MHz, $CDCl_3$) δ 178.0, 170.7, 144.3, 129.4, 128.0, 126.8, 66.5, 61.7, 50.9, 38.7, 34.0, 27.4, 14.1. **HRMS** (NSI$^+$): Calc. for $C_{29}H_{33}NO_3SNa$: 498.2073, found 498.2070 $[M+Na]^+$.

Synthesis of **S3**

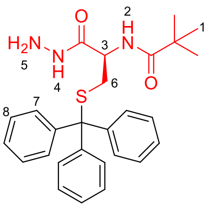

Hydrazine hydrate (45.0 µL, 0.926 mmol) was added to a solution of **S2** (153 mg, 0.322 mmol) in MeOH (2 mL) and the reaction was stirred at reflux for 67 hours. The crude mixture was concentrated under reduced pressure and purified by flash column chromatography (SiO_2, MeOH/CH_2Cl_2, 0% → 4%) to afford **S3** as

colourless solid (133 mg, 0.288 mmol, 89%). **M.p.** 88 °C. **^{1}H NMR** (600 MHz, $CDCl_3$) δ 7.42 (d, *J* = 7.2 Hz, 6H, H_7), 7.30 (t, *J* = 7.2 Hz, 6H, H_8), 7.23 (t, *J* = 7.2 Hz, 3H, H_9), 7.18 (br, s, 1H, H_4), 6.03 (d, *J* = 7.3 Hz, 1H, H_2), 3.98 (q, *J* = 6.9 Hz, 1H, H_3), 3.77 (br, s, 2H, H_5), 2.68 – 2.53 (m, 2H, H_6), 1.15 (s, 9H, H_1). **^{13}C NMR** (151 MHz, $CDCl_3$) δ 178.7, 170.9, 144.2, 129.5, 128.1, 126.9, 67.1, 50.7, 38.7, 33.4, 27.4. **HRMS** (NSI^+): Calc. for $C_{27}H_{31}N_3O_2SNa$: 484.2029, found 484.2015 $[M+Na]^+$.

Synthesis of **3**

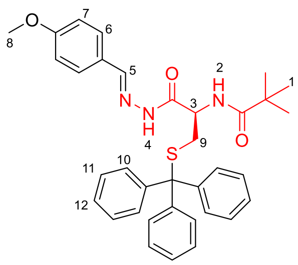

p-Anisaldehyde (49 µL, 0.400 mmol) was added to a solution of **S3** (92.3 mg, 0.200 mmol) and acetic acid (2 drops) in CH_2Cl_2 (1.2 mL). The reaction mixture was stirred at room temperature for 24 hours. The crude mixture was concentrated under reduced pressure and purified by flash column chromatography (SiO_2, MeOH/CH_2Cl_2, 0% → 2%) to afford **3** as a pale yellow solid (110 mg, 0.190 mmol, 95%). **M.p.** 105 °C. **^{1}H NMR** (600 MHz, $CDCl_3$) ~3:2 *cis*:*trans* hydrazone amide rotamers. *Trans*-**3** δ 9.58 (s, 1H, H_4), 7.93 (s, 1H, H_5), 7.63 (d, *J* = 8.8 Hz, 2H, H_6), 7.43 (d, *J* = 7.1 Hz, 6H, H_{10}), 7.28 (t, *J* = 7.1 Hz, 6H, H_{11}), 7.21 (t, *J* = 7.1 Hz, 3H, H_{12}), 6.87 (d, *J* = 8.8 Hz, 2H, H_7), 6.16 (d, *J* = 7.5 Hz, 1H, H_2), 4.05 – 4.01 (m, 1H, H_3), 3.82 (s, 3H, H_8), 2.74 – 2.65 (m, 2H, H_9), 1.17 (s, 9H, H_1). *Cis*-**3** δ 8.66 (s, 1H, H_4), 7.55 (s, 1H, H_5), 7.45 (d, *J* = 8.8 Hz, 2H, H_6), 7.35 (d, *J* = 7.4 Hz, 6H, H_{10}), 7.15 (t, *J* = 7.4 Hz, 6H, H_{11}), 7.09 (t, *J* = 7.3 Hz, 3H, H_{12}), 6.90 (d, *J* = 8.8 Hz, 2H, H_7), 6.72 (d, *J* = 7.9 Hz, 1H, H_2), 5.49 – 5.46 (m, 1H, H_3), 3.87 (s, 3H, H_8), 2.93 – 2.90 (m, 1H, H_9), 2.56 – 2.53 (m, 1H, $H_{9'}$), 1.27 (s, 9H, H_1). **^{13}C NMR** (151 MHz, $CDCl_3$) δ 179.6, 178.0, 171.1, 166.7, 161.7, 161.6, 148.7, 144.6, 144.5, 129.7, 129.6, 129.2, 128.2, 128.0, 127.0, 126.8, 126.2, 125.9, 114.3, 114.2, 67.3, 66.4, 55.6, 55.5, 51.7, 49.5, 39.0, 38.9, 34.0, 32.9, 27.7, 27.5. **HRMS** (NSI^+): Calc. for $C_{35}H_{37}N_3O_3SNa$: 602.2448, found 602.2418 $[M+Na]^+$.

Synthesis of *EZ*-**6**

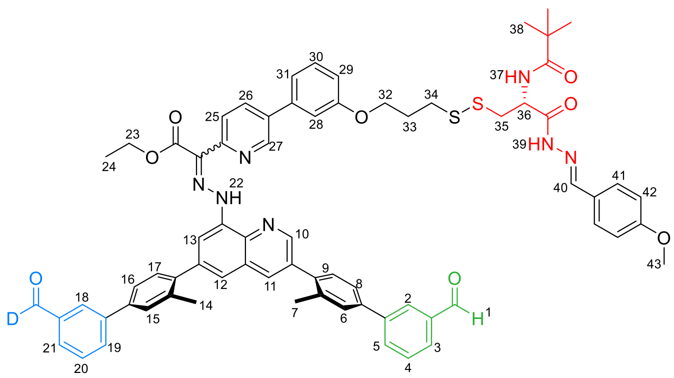

$AgNO_3$ (2.3 mg, 14 µmol) was added to a solution of **3** (6.5 mg, 11 µmol) in a 2:1 $CHCl_3$:MeOH mixture (300 µL), the mixture was stirred at room temperature for 30 minutes. A solution of *EZ*-**2** (18.3 mg, 19 µmol) in $CHCl_3$ (50 µL) was added and the mixture was stirred at room temperature for 3 hours. The reaction mixture was diluted with $CHCl_3$, washed with brine, dried over Na_2SO_4, filtered and concentrated under reduced pressure. Preparative thin layer chromatography (SiO_2, EtOAc/CH_2Cl_2 (0.2% Et_3N), 10%) afforded *EZ*-**6**

as an orange solid (10.6 mg, 8.75 µmol, 79%). **^{1}H NMR** (600 MHz, $CDCl_3$) ~62:38 *E*:*Z* pyridyl-hydrazone isomers and mixture of acyl-hydrazone rotamers. Major isomer reported (*E*-**7**) δ 15.82 (s, 1H, H_{22}), 10.16 – 10.14 (m, 1H, H_1), 9.16 (m, 1H, H_{27}), 9.05 – 8.98 (m, 1H, H_{10}), 8.98 – 8.85 (m, 1H, H_{39}), 8.36 – 8.29 (m, 1H, H_{25}), 8.24 – 7.36 (m, 25H, H_3+ H_2+ H_4+ H_5+ H_6+ H_8+ H_9+ H_{11}+ H_{12}+ H_{13}+ H_{15}+ H_{16}+ H_{17}+ H_{18}+ H_{19}+ H_{20}+ H_{21}+ H_{26}+ H_{28}+ H_{29}+ H_{30}+ H_{31}+ H_{40}+ H_{41}), 6.90 (d, *J* = 8.3 Hz, 2H, H_{42}), 6.79 (m, 1H, H_{37}), 5.78 – 5.62 (m, 1H, H_{36}), 4.47 (q, *J* = 7.2 Hz, 2H, H_{23}), 4.18 – 4.09 (m, 2H, H_{32}), 3.81 (s, 3H, H_{43}), 3.42 – 3.41 (m, 1H, H_{35}), 3.21 – 3.19 (m, 1H, $H_{35'}$), 2.99 – 2.95 (m, 2H, H_{34}), 2.50 (s, 6H, H_7+ H_{14}), 2.27 – 2.18 (m, 2H, H_{33}), 1.48 (t, *J* = 7.2 Hz, 3H, H_{24}), 1.26 (m, 9H, H_{38}). **LRMS** (ESI+) 1211.44 (35%, $[M+H]^+$), 1257.46 (100%, $[M+OC_2H_6+H]^+$).

<u>Synthesis of *E*-**9**</u>

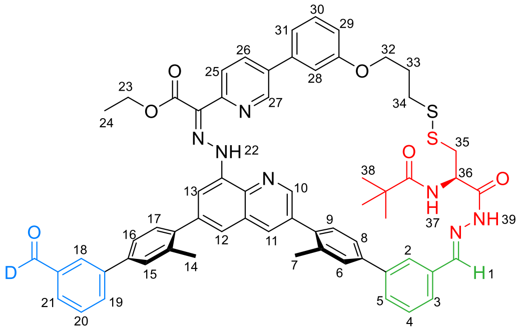

EZ-**6** (9.8 mg, 8.1 µmol) and trifluoroacetic acid (44 µL, 0.568 mmol) were dissolved in $CHCl_3$ (8.1 mL, 1.0 mM) and stirred at room temperature for 17 hours. Triethylamine (160 µL, 1.15 mmol) was added and the reaction mixture was washed with a saturated aqueous solution of $NaHCO_3$. The aqueous layer was washed with CH_2Cl_2 and the combined organic layers were dried over Na_2SO_4, filtered and concentrated under reduced pressure. Preparative thin layer chromatography (SiO_2, EtOAc/CH_2Cl_2 (0.2% Et_3N), 20%) afforded *E*-**9** (4.6 mg, 4.3 µmol, 53%) and *Z*-**9** (1.5 mg, 1.4 µmol, 17%) as yellow solids. **^{1}H NMR** (600 MHz, $CDCl_3$) *E*-**9** δ 16.28 (s, 1H, H_{22}), 9.21 (d, *J* = 2.4 Hz, 1H, H_{27}), 9.05 (d, *J* = 2.2 Hz, 1H, H_{10}), 9.03 (s, 1H, H_{39}), 8.32 (d, *J* = 8.5 Hz, 1H, H_{25}), 8.22 – 8.21 (m, 3H, H_2+ H_{11}+ H_{18}), 8.10 (dd, *J* = 8.6, 2.5 Hz, 1H, H_{26}), 8.04 (d, *J* = 2.2 Hz, 1H, H_{13}), 7.98 (dt, *J* = 7.9, 1.4 Hz, 1H, H_{19}), 7.90 (dt, *J* = 7.6, 1.4 Hz, 1H, H_5), 7.84 (s, 1H, H_1), 7.72 (d, *J* = 7.9 Hz, 1H, H_3), 7.66 (t, *J* = 7.5 Hz, 1H, H_{20}), 7.64 – 7.59 (m, 4H, H_4+H_6+ H_{15}+ H_8), 7.56 – 7.50 (m, 3H, H_9+ H_{16}+ H_{21}), 7.47 (d, *J* = 1.7 Hz, 1H, H_{12}), 7.41 (t, *J* = 7.9 Hz, 1H, H_{30}), 7.36 – 7.34 (m, 2H, H_{28}+ H_{17}), 7.25 (d, *J* = 6.8 Hz, 1H, H_{31}), 6.95 (dd, *J* = 8.2, 2.4 Hz, 1H, H_{29}), 6.87 (d, *J* = 7.7 Hz, 1H, H_{37}), 5.74 – 5.70 (m, 1H, H_{36}), 4.45 (q, *J* = 7.1 Hz, 2H, H_{23}), 4.16 – 4.12 (m, 2H, H_{32}), 3.58 (dd, *J* = 14.2, 4.2 Hz, 1H, H_{35}), 3.26 (dd, *J* = 14.2, 6.9 Hz, 1H, $H_{35'}$), 3.01 – 2.88 (m, 2H, H_{34}), 2.51 (s, 6H, H_7+ H_{14}), 2.31 – 2.21 (m, 2H, H_{33}), 1.46 (t, *J* = 7.1 Hz, 3H, H_{24}), 1.29 (s, 9H, H_{38}). **^{13}C NMR** (151 MHz, $CDCl_3$) *E*-**9** δ 178.5, 171.3, 165.9, 159.6, 151.3, 150.4, 145.3, 144.9, 142.0, 141.8, 141.4, 140.8, 139.4, 139.0, 138.4, 137.7, 137.0, 136.7, 136.6, 136.3, 135.2, 134.8, 134.7, 134.6, 133.6, 133.2, 131.0, 130.8, 130.5, 129.9, 129.7, 129.5, 129.4, 129.3, 129.2, 128.9, 128.4, 128.2, 127.8, 125.4, 124.7, 123.9, 120.5, 119.8, 115.1, 112.1, 112.0, 66.2, 61.3, 51.5, 39.6, 39.0, 34.8, 32.1, 28.6, 27.6, 21.0, 14.6. **HRMS** (NSI^+): Calc. for $C_{63}H_{58}DN_7O_6S_2H$: 1075.4104, found 1075.4100 $[M+H]^+$.

Synthesis of *Z*-**9**

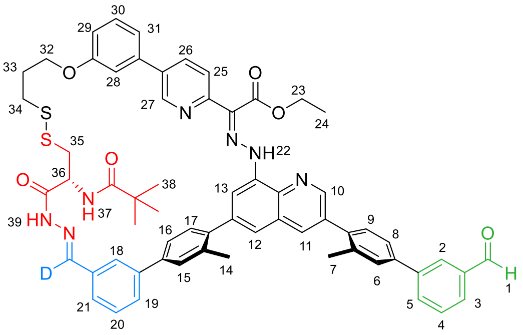

EZ-**6** (9.8 mg, 8.1 µmol) and trifluoroacetic acid (2µL, 26.1 µmol) were dissolved in $CHCl_3$ (3.3 mL) and stirred at room temperature for 41 hours. Triethylamine (8 µL, 57.5 1 µmol) was added and the reaction mixture was washed with a saturated aqueous solution of $NaHCO_3$. The aqueous layer was washed with CH_2Cl_2 and the combined organic layers were dried over Na_2SO_4, filtered and concentrated under reduced pressure. Preparative thin layer chromatography (SiO_2, EtOAc/CH_2Cl_2 (0.2% Et_3N), 20%) afforded *Z*-**9** as a yellow solid (5.6 mg, 5.2 µmol, 64%). **^{1}H NMR** (600 MHz, $CDCl_3$) ~63:37 *cis*:*trans* hydrazone amide rotamers. δ 13.63 (s, 1H, $H_{22\textit{cis}}$), 13.38 (s, 1H, $H_{22\textit{trans}}$), 10.24 (s, 1H, H_1), 10.13 (s, 1H, $H_{39\textit{trans}}$), 9.01 (d, *J* = 2.2 Hz, 1H, H_{10}), 8.99 (s, 1H, $H_{39\textit{cis}}$), 8.88 (s, 1H, H_{27}), 8.27 – 8.14 (m, 3H, H_2+ H_{11}+ H_{26}), 8.06 – 7.85 (m, 6H, H_3+ H_5+ H_{13}+ H_{18}+ H_{19} +H_{25}), 7.70 – 7.61 (m, 4H, H_4+ H_6+ H_{15}+ H_{20}), 7.57 – 7.43 (m, 6H, H_8+ H_9+ H_{16}+ H_{17}+ H_{21}+ H_{31}), 7.40 – 7.35 (m, 2H, H_{12}+ H_{30}), 7.22 (t, *J* = 1.8 Hz, 1H, $H_{28\textit{trans}}$), 7.16 (t, *J* = 1.8 Hz, 1H, $H_{28\textit{cis}}$), 7.08 (d, J = 7.7 Hz, 1H, $H_{37\textit{cis}}$), 6.95 (m, 1H, H_{29}), 6.87 (d, *J* = 7.7 Hz, 1H, $H_{37\textit{trans}}$), 5.74 – 5.70 (m, 1H, $H_{36\textit{cis}}$), 4.79 (q, *J* = 6.5 Hz, 1H, $H_{36\textit{trans}}$), 4.45 (m, 2H, H_{23}), 4.16 – 4.12 (m, 2H, H_{32}), 3.46 – 3.31 (m, 2H, H_{35}), 3.01– 2.88 (m, 2H, H_{34}), 2.51 (m, 6H, H_7+ H_{14}), 2.31 – 2.21 (m, 2H, H_{33}), 1.46 (t, *J* = 7.1 Hz, 3H, H_{24}), 1.29 (s, 9H, H_{38}). **^{13}C NMR** (151 MHz, $CDCl_3$) δ 192.5, 178.1, 171.1, 159.6, 159.4, 149.7, 149.6, 142.3, 142.0, 141.7, 141.6, 141.3, 141.1, 140.2, 140.0, 139.7, 138.9, 138.1, 137.1, 136.8, 136.5, 136.1, 135.8, 135.7, 135.5, 135.4, 135.3, 134.0, 133.4, 133.2, 131.1, 130.5, 130.4, 130.0, 129.8, 129.6, 129.3, 129.1, 128.2, 128.1, 128.0, 126.1, 125.1, 125.0, 124.1, 123.3, 119.5, 119.3, 114.7, 114.0, 113.6, 112.7, 135.8, 129.2, 128.6, 127.2, 70.7, 66.3, 65.8, 63.3, 61.7, 54.8, 52.3, 51.1, 50.1, 45.5, 43.2, 41.9, 40.7, 39.4, 39.2, 39.0, 36.5, 35.3, 35.0, 34.1, 33.7, 33.0, 28.5, 27.6, 22.8, 20.9, 14.5, 14.3. **HRMS** (NSI$^+$): Calc. for $C_{63}H_{58}DN_7O_6S_2H$: 1075.4104, found 1075.4078 $[M+H]^+$.

2.2 Synthesis of *EZ*-10

2.2.1 Synthetic Scheme

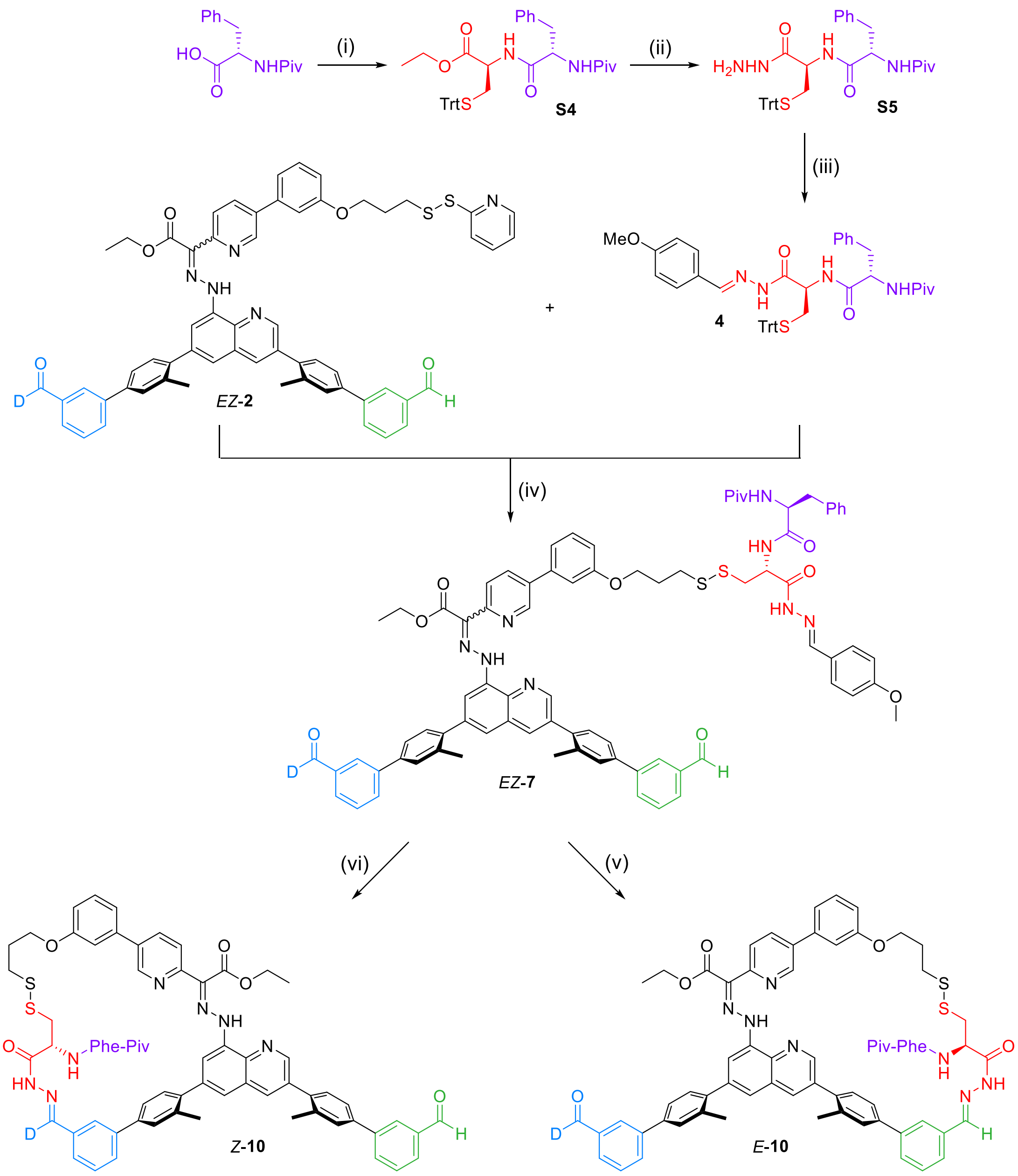

Scheme 2. Synthesis of *Z*-**10** and *E*-**10**. Reagents and conditions: (i) **S1**, HOBt·H_2O, EDCI·HCl, Et_3N, CH_2Cl_2, r.t., 17 h, 99%. (ii) NH_2NH_2·H_2O, MeOH, Δ, 17 h, 65%. (iii) *p*-anisaldehyde, AcOH, CH_2Cl_2, r.t., 18 h, 72%. (iv) $AgNO_3$, $CHCl_3$, MeOH, r.t., 40 mins, then *EZ*-**2** in $CHCl_3$, r.t., 3 h, 69%. (v) TFA, $CHCl_3$, r.t., 16 h, 70%. (vi) TFA, $CHCl_3$, r.t., 65 h, 38%.

2.2.2 Synthetic Procedures and Characterisation Data

Synthesis of **S4**

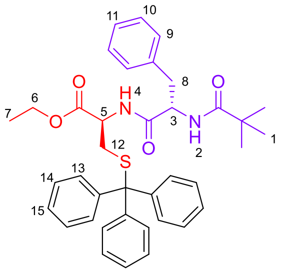

To a solution of **S1** (430 mg, 1.10 mmol) in CH_2Cl_2 (20 mL) was added triethylamine (170 µL, 1.22 mmol), HOBt·H_2O (166 mg, 1.23 mmol), EDCI·HCl (240 mg, 1.25 mmol) and pivaloylphenylalanine (299 mg, 1.20 mmol). The reaction mixture was stirred at room temperature for 17 hours. The reaction mixture was diluted with CH_2Cl_2, washed with a saturated aqueous solution of $NaHCO_3$, a 1 M aqueous solution of HCl, and brine. The organic layer was dried over $MgSO_4$, filtered and concentrated under reduced pressure. Flash column chromatography (SiO_2, MeOH/CH_2Cl_2, 0% → 5%) afforded **S4** as a colourless solid (679 mg, 1.09 mmol, 99%). **M.p.** 61 °C. **^{1}H NMR** (600 MHz, DMSO-d_6) ~1:1 mixture of rotamers. δ 8.47–8.41 (m, 1H, H_4), 7.38 (d, J = 8.6 Hz, 1H, H_2), 7.36 – 7.19 (m, 19H, H_{13}+ H_{14}+ H_{15}+ H_9+ H_{10}), 7.17 – 7.14 (m, 1H, H_{11}), 4.59 – 4.54 (m, 1H, H_3), 4.16 – 4.12 (m, 1H, H_5), 4.05 – 3.99 (m, 2H, H_6), 3.04 – 2.98 (m, 1H, H_8), 2.89 – 2.84 (m, 1H, $H_{8'}$), 2.57 – 2.52 (m, 1H, H_{12}), 2.42 – 2.36 (m, 1H, $H_{12'}$), 1.12 – 1.09 (m, 3H, H_7), 0.97 – 0.96 (m, 9H, H_1). **^{13}C NMR** (151 MHz, DMSO-d_6) δ 177.1, 171.8, 171.6, 170.1, 144.1, 138.0, 129.4, 129.1, 128.2, 127.9, 127.2, 126.9, 126.2, 66.4, 66.3 61.0, 60.9, 53.8, 53.6, 51.8, 51.3, 38.0, 37.0, 33.3, 32.8, 27.2, 14.0. **HRMS** (NSI$^+$): Calc. for $C_{38}H_{42}N_2O_4SNa$: 645.2757, found 645.2732 $[M+Na]^+$.

Synthesis of **S5**

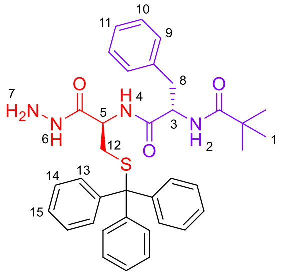

Hydrazine hydrate (15.0 µL, 0.309 mmol) was added to a solution of **S4** (156 mg, 0.250 mmol) in MeOH (1 mL) and the reaction mixture was stirred at reflux for 17 hours. The reaction mixture was concentrated under reduced pressure and purified by flash column chromatography (SiO_2, MeOH/CH_2Cl_2, 0% → 4%) to afford **S5** as a colourless solid (98.5 mg, 0.162 mmol, 65%).**^{1}H NMR** (600 MHz, $CDCl_3$) ~1:2 *cis*:*trans* mixture of hydrazone amide rotamers. Major rotamer reported. δ 7.79 (s, 2H, H_6), 7.38 (d, J = 8.3 Hz, 6H, H_{13}), 7.29 – 7.11 (m, 14H, H_{14}+ H_{15}+ H_9+ H_{10}+ H_{11}), 6.85 (d, J = 7.0 Hz, 1H, H_4), 6.31 (d, J = 7.1 Hz, 1H, H_2), 4.54 (q, J = 7.0 Hz, 1H, H_3), 4.02 (q, J = 7.3, 1H, H_5), 3.76 (s, 3H, H_7), 3.04 (dd, J = 14.0, 7.0 Hz, 1H, H_8), 2.95 (dd, J = 13.8, 7.2 Hz, 1H, $H_{8'}$), 2.59 (dd, J = 13.0, 7.6 Hz, 1H, H_{12}), 2.53 (dd, J = 13.3, 5.9 Hz, 1H, $H_{12'}$), 1.07 (s, 9H, H_1). **^{13}C NMR** (151 MHz, $CDCl_3$) δ 179.1, 171.2, 170.0, 144.3, 136.1, 129.5, 129.3, 128.6, 128.0, 127.0, 126.9, 67.2, 54.4, 51.2, 38.6, 37.9, 33.3, 27.3, 18.4. **HRMS** (NSI$^+$): Calc. for $C_{36}H_{40}N_4O_3SNa$: 631.2713, found 631.2736 $[M+Na]^+$.

<u>Synthesis of **4**</u>

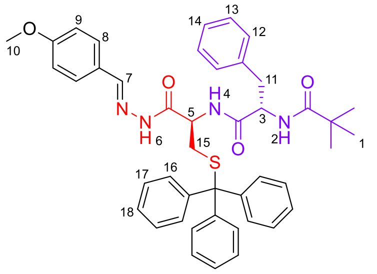

S5 (98.5 mg, 0.162 mmol), *p*-anisaldehyde (24 μL, 0.200 mmol) and acetic acid (2 drops) were combined in CH_2Cl_2 (1.6 mL) and stirred at room temperature for 18 hours. The reaction mixture was concentrated under reduced pressure and purified by flash column chromatography (SiO_2, MeOH/CH_2Cl_2, 0% → 2.5%) to afford **4** as a colourless solid (85.1 mg, 0.117 mmol, 72%). Preparative thin layer chromatography (SiO_2, EtOAc/CH_2Cl_2, 20% × 2) was used to separate the different diastereoisomers. **M.p.** 212 °C. **^{1}H NMR** (600 MHz, $CDCl_3$) ~43:57 *cis*:*trans* hydrazone amide rotamers. *Trans*-**4** δ 9.96 (s, 1H, H_6), 8.29 (s, 1H, H_7), 7.66 (d, *J* = 6.7 Hz, 2H, H_8), 7.41 – 7.17 (m, 19H, H_{16}+ H_{17}+ H_{18}+ H_{12}+ H_{13}), 7.13 – 7.10 (m, 1H, H_{14}), 6.90–6.87 (m, 2H, H_9), 5.99 (d, *J* = 4.9 Hz, 1H, H_2), 5.93 (d, *J* = 8.2 Hz, 1H, H_4), 4.37 – 4.33 (m, 1H, H_5), 4.06 – 4.02 (m, 1H, H_3), 3.82 (s, 3H, H_{10}), 3.10 – 3.07 (m, 1H, H_{11}), 2.98 – 2.90 (m, 3H, H_{15}+$H_{11'}$), 2.63 – 2.60 (m, 1H, $H_{15'}$), 1.08 (s, 9H, H_1). *Cis*-**4** δ9.42 (s, 1H, H_6), 7.64 (s, 1H, H_7), 7.46 (d, *J* = 8.8 Hz, 1H, H_8), 7.41 – 7.17 (m, 19H, H_{16}+ H_{17}+ H_{18}+ H_{12}+ H_{13}), 7.14 (d, *J* = 7.3 Hz, 1H, H_4), 7.13 – 7.10 (m, 1H, H_{14}), 6.90 – 6.87 (m, 2H, H_9), 6.29 (d, *J* = 8.1 Hz, 1H, H_2), 5.46 – 5.43 (m, 1H, H_5), 5.10 – 5.06 (m, 1H, H_3), 3.86 (s, 3H, H_{10}), 3.24 – 3.20 (m, 1H, H_{11}), 3.05 – 3.01 (m, 1H, $H_{11'}$), 2.98 – 2.90 (m, 1H, H_{15}), 2.48 – 2.45 (m, 1H, $H_{15'}$), 1.08 (s, 9H, H_1). **^{13}C NMR** (151 MHz, $CDCl_3$) δ 180.2, 178.4, 171.2, 165.8, 161.5, 149.3, 144.6, 136.7, 129.7, 129.5, 129.2, 129.0, 128.5, 128.2, 128.0, 127.6, 127.1, 127.0, 126.8, 126.7, 114.3, 114.2, 67.4, 66.7, 56.1, 55.5, 53.4, 51.8, 49.7, 38.8, 36.8, 34.1, 33.4, 29.9, 27.5, 27.4. **HRMS** (NSI$^+$): Calc. for $C_{44}H_{46}N_4O_4SNa$: 749.3132, found 749.3119 $[M+Na]^+$.

<u>Synthesis of *EZ*-**7**</u>

$AgNO_3$ (4.3 mg, 25 μmol) was added to a solution of **4** (15.4 mg, 21.2 μmol) in a 2:1 $CHCl_3$:MeOH

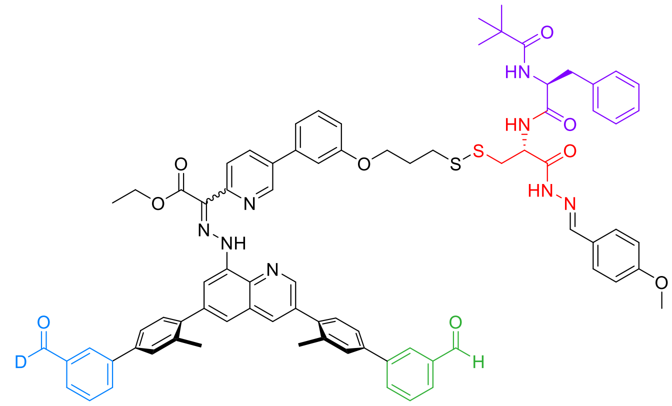

mixture (600 μL) and the mixture was stirred at room temperature for 40 minutes, a solution of *EZ*-**2** (31.3 mg, 31.8 μmol) in $CHCl_3$ (200 μL) was added and the reaction was stirred at room temperature for 3 hours. The reaction mixture was diluted with $CHCl_3$, washed with brine, dried over Na_2SO_4, filtered and concentrated under reduced pressure. Preparative thin layer chromatography (SiO_2, EtOAc/CH_2Cl_2 (0.1% Et_3N), 30%) afforded *EZ*-**7** as a yellow solid (20.0 mg, 14.7 μmol, 69%). **^{1}H NMR** (600 MHz, $CDCl_3$) ~62:38 *E*:*Z* pyridyl-hydrazone isomers and mixture of acyl-hydrazone rotamers. δ 15.82 – 15.78 (m, 1H), 13.48 – 13.41 (m, 1H), 10.35 – 10.31 (m, 1H), 10.15 – 10.05 (m, 2H), 9.16 – 9.10 (m, 1H), 9.05 –

8.96 (m, 2H), 8.87 – 8.85 (m, 1H), 8.37 (d, *J* = 6.6 Hz, 1H), 8.31 – 8.27 (m, 1H), 8.23 – 8.07 (m, 5H), 8.05 – 8.00 (m, 1H), 7.99 – 7.85 (m, 5H), 7.84 – 7.74 (m, 3H), 7.69 – 7.53 (m, 14H), 7.51 – 7.29 (m, 11H), 7.22 – 7.12 (m, 5H), 6.98 – 6.81 (m, 5H), 6.41 – 6.33 (m, 1H), 6.21 – 6.12 (m, 1H), 5.74 (q, *J* = 6.0 Hz, 1H), 5.50 – 5.33 (m, 1H), 5.18 (quint., *J* = 6.6 Hz, 1H), 4.92 – 4.85 (m, 1H), 4.53 (q, *J* = 7.2 Hz, 1H), 4.43 (q, *J* = 7.2 Hz, 2H), 4.24 – 4.18 (m, 1H), 4.16 – 4.06 (m, 3H), 3.83 – 3.74 (m, 5H), 3.43 – 3.35 (m, 6H), 3.34 – 3.28 (m, 1H), 3.25 – 3.21 (m, 1H), 3.20 – 3.14 (m, 2H), 3.09 – 2.96 (m, 3H), 2.95 – 2.85 (m, 4H), 2.52 – 2.42 (m, 9H), 2.25 – 2.16 (m, 4H), 1.47 – 1.41 (m, 5H), 1.25 (s, 5H), 1.17 – 1.14 (m, 7H), 1.13 – 1.08 (m, 2H), 1.07 – 1.05 (m, 6H). **HRMS** (NSI$^+$): Calc. for $C_{80}H_{75}DN_8O_9S_2H$: 1358.5312; found 1358.5290 $[M+H]^+$.

<u>Synthesis of *E*-**10**</u>

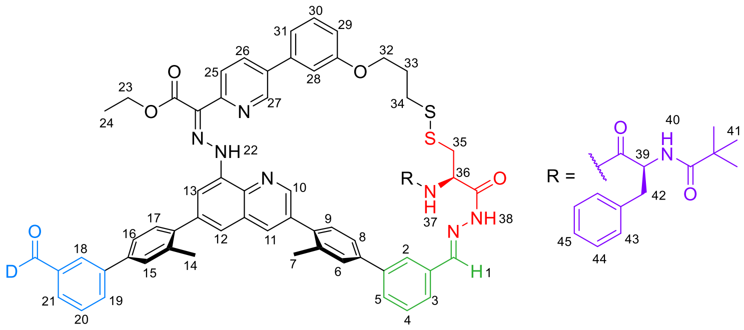

EZ-**7** (10.0 mg, 7.35 µmol) and trifluoroacetic acid (40 µL, 0.515 mmol) were dissolved in $CHCl_3$ (7.4 mL) and stirred at room temperature for 16 h. Triethylamine (143 µL, 1.03 mmol) was added and the mixture was washed with a saturated aqueous solution of $NaHCO_3$. The aqueous layer was extracted with CH_2Cl_2 and the combined organic layers were dried over Na_2SO_4, filtered and concentrated under reduced pressure. Preparative thin layer chromatography (SiO_2, EtOAc/CH_2Cl_2 (0.1% Et_3N), 30%) afforded *E*-**10** as a yellow solid (6.3 mg, 5.2 µmol, 70%) and *Z*-**10** as a yellow solid (2.0 mg, 1.6 µmol, 22%). **^{1}H NMR** (600 MHz, Chloroform-d) ~87:13 *cis*:*trans* hydrazone amide rotamers, major rotamer reported δ 16.34 (s, 1H, H_{22}), 10.53 (s, br, 1H, H_{38}), 9.26 (d, *J* = 2.4 Hz, 1H, H_{27}), 9.11 (d, *J* = 2.1 Hz, 1H, H_{10}), 8.37 – 8.34 (m, 2H, H_{25}+ H_2), 8.28 – 8.21 (m, 2H, H_{11}+ H_{18}), 8.14 (dd, *J* = 8.6, 2.5 Hz, 1H, H_{26}), 8.10 – 8.04 (m, 2H, H_{13}+ H_1), 8.01 (d, *J* = 7.5 Hz, 1H, H_{19}), 7.93 (dt, *J* = 7.5 Hz, 1H, H_5), 7.76 – 7.65 (m, 7H, H_4+ H_6+ H_8+ H_{15}+ H_{16}+ H_{20}+ H_{37}), 7.64 – 7.62 (m, 1H, H_{17}), 7.60 – 7.57 (m, 2H, H_3+ H_{21}), 7.53 – 7.48 (m, 2H, H_{12}+ H_9), 7.46 (t, *J* = 7.8 Hz, 1H, H_{30}), 7.41 (s, 1H, H_{28}), 7.33 – 7.30 (m, 4H, H_{31}+ H_{43}+ H_{45}), 7.27 – 7.24 (m, 2H, H_{44}), 7.00 (dd, *J* = 8.2, 2.0 Hz, 1H, H_{29}), 6.30 (d, *J* = 8.3 Hz, 1H, H_{40}), 5.84 (dt, *J* = 7.2, 6.4 Hz, 1H, H_{36}), 5.25 (dt, *J* = 8.1, 6.8 Hz, 1H, H_{39}), 4.48 (q, *J* = 7.2 Hz, 2H, H_{23}), 4.27 – 4.11 (m, 2H, H_{32}), 3.44 (dd, *J* = 13.3, 5.7 Hz, 1H, H_{35}), 3.32 (dd, *J* = 14.0, 6.1 Hz, 1H, H_{42}), 3.17 (dd, *J* = 13.3, 5.7 Hz, 1H, $H_{35'}$), 3.06 (dd, *J* = 14.0, 6.1 Hz, 1H, $H_{42'}$), 3.02 – 2.92 (m, 2H, H_{34}), 2.57 (s, 3H, H_7), 2.54 (s, 3H, H_{14}), 2.36 – 2.32 (m, 2H, H_{33}), 1.50 (t, *J* = 7.2 Hz, 3H, H_{24}), 1.05 (s, 9H, H_{41}). **^{13}C NMR** (151 MHz, $CDCl_3$) δ 178.7, 171.2, 171.1, 165.8, 159.6, 159.6, 151.1, 150.4, 145.9, 145.2, 141.9, 141.7, 141.6, 141.3, 140.6, 139.2, 138.9, 138.3, 137.4, 136.9, 136.6, 136.5, 136.4, 136.2, 135.1, 134.7, 134.5, 134.1, 133.1, 130.9, 130.7, 130.4, 129.9, 129.6, 129.5, 129.2,

129.1, 129.0, 128.8, 128.5, 128.3, 128.1, 127.7, 126.9, 125.4, 124.6, 123.8, 120.3, 119.9, 114.7, 112.3, 111.8, 66.3, 61.2, 53.4, 49.9, 40.1, 39.1, 38.6, 34.5, 31.9, 29.7, 29.6, 28.7, 27.4, 27.3, 27.3, 27.2, 22.7, 20.9, 20.9, 14.5, 14.2. **HRMS** (ESI$^+$): Calc. for $C_{72}H_{67}DN_8O_7S_2H$: 1222.4788; found 1222.4751 $[M+H]^+$.

Synthesis of *Z*-**10**

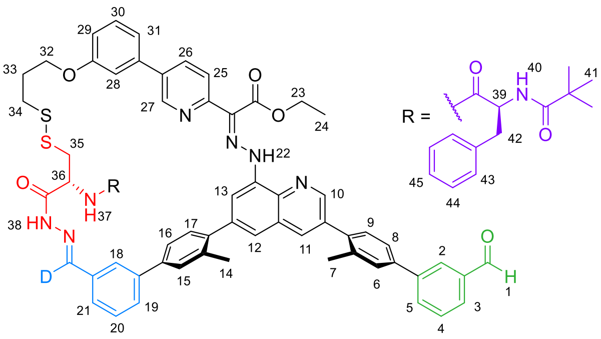

EZ-**7** (10.0 mg, 7.35 µmol) and trifluoroacetic acid (2 µL, 22 µmol) were dissolved in $CHCl_3$ (3.0 mL) and stirred at room temperature for 65 hours. Triethylamine (6 µL, 44 µmol) was added and the mixture was washed with a saturated aqueous solution of $NaHCO_3$. The aqueous layer was extracted with CH_2Cl_2 and the combined organic layers were dried over Na_2SO_4, filtered and concentrated under reduced pressure. Preparative thin layer chromatography (SiO_2, EtOAc/CH_2Cl_2 (0.1% Et_3N), 30%) afforded *Z*-**10** as a yellow solid (3.4 mg, 2.8 µmol, 38%). **^{1}H NMR** (600 MHz, $CDCl_3$) ~22:78 *cis*:*trans* hydrazone amide rotamers. δ 13.65 (s, 1H, H_{22cis}), 13.44 (s, 1H, $H_{22trans}$), 10.59 (s, 1H, $H_{38trans}$), 10.16 (s, 1H, H_1), 9.01 (d, *J* = 2.1 Hz, 1H, H_{10cis}), 8.99 (d, *J* = 2.1 Hz, 1H, $H_{10trans}$), 8.93 (m, 2H, H_{27}+ H_{38cis}), 8.46 (t, *J* = 1.8 Hz, 1H, H_{18}), 8.22 (m, 1H, H_2), 8.18 (d, *J* = 2.2 Hz, 1H, H_{11}), 8.09 (dd, *J* = 8.2, 2.3 Hz, 1H, H_{26}), 7.99 – 7.92 (m, 6H, H_3+ H_5+ H_{13}+ H_{19}+ H_{21}+ H_{25}), 7.73 – 7.67 (m, 4H, H_6+ H_4+ H_{20}+ H_8), 7.66 – 7.62 (m, 2H, H_{15}+ H_{16}), 7.56 (d, *J* = 7.8 Hz, 1H, H_{37}), 7.53 – 7.47 (m, 3H, H_{12}+ H_{17}+ H_9), 7.43 – 7.39 (m, 2H, H_{29}+ H_{30}), 7.37 – 7.34 (m, 2H, H_{43}), 7.31 (d, *J* = 7.8 Hz, 1H, H_{45}), 7.28 – 7.23 (m, 3H, H_{31}+ H_{44}), 6.94 (dd, *J* = 9.8, 8.1 Hz, 1H, H_{28}), 6.36 (d, *J* = 8.7 Hz, 1H, $H_{40trans}$), 6.21 (d, *J* = 4.7 Hz, 1H, $H_{36trans}$), 6.19 (d, *J* = 8.7 Hz, 1H, H_{40cis}), 5.76 (m, 1H, H_{36cis}), 5.12 (m, 1H, H_{39cis}), 4.95 (dt, *J* = 9.4, 5.1 Hz, 1H, $H_{39trans}$), 4.62 – 4.49 (m, 2H, H_{23}), 4.23 – 4.14 (m, 2H, H_{32}), 3.56 (dd, *J* = 13.5, 5.2 Hz, 1H, H_{42}), 3.22 (dd, *J* = 13.1, 8.4 Hz, 1H, H_{35}), 3.15 (dd, *J* = 13.1, 7.7 Hz, 1H, $H_{35'}$), 2.97 – 2.89 (m, 2H, H_{34}), 2.86 (dd, *J* = 13.5, 4.8 Hz, 1H, $H_{42'}$), 2.50 (s, 3H, H_7), 2.47 (s, 3H, H_{14}), 2.31 – 2.15 (m, 2H, H_{33}), 1.50 (t, *J* = 7.2 Hz, 3H, H_{24}), 1.27 (s, 9H, H_{41}). **^{13}C NMR** (151 MHz, $CDCl_3$) δ 192.4, 180.7, 171.1, 166.1, 163.2, 159.4, 154.2, 149.4, 146.9, 141.9, 141.6, 141.4, 140.9, 140.1, 139.6, 138.5, 138.1, 137.0, 136.7, 135.9, 135.6, 135.4, 135.2, 134.8, 134.5, 133.1, 130.9, 130.1, 130.0, 129.8, 129.6, 129.5, 129.4, 129.1, 129.0, 128.9, 128.8, 128.6, 128.4, 128.1, 128.0, 127.9, 127.6, 125.1, 125.0, 124.7, 123.9, 123.2, 119.8, 119.3, 113.9, 113.8, 112.7, 66.8, 61.6, 61.6, 57.6, 50.6, 38.7, 38.6, 38.2, 36.7, 35.2, 34.4, 31.9, 29.7, 29.6, 29.4, 29.2, 27.4, 27.3, 22.7, 20.8, 14.3, 14.2, 14.1. **HRMS** (ESI$^+$): Calc. for $C_{72}H_{67}DN_8O_7S_2H$: 1222.4788; found 1222.4742 $[M+H]^+$.

2.3 Synthesis of *EZ*-11

2.3.1 Synthetic Scheme

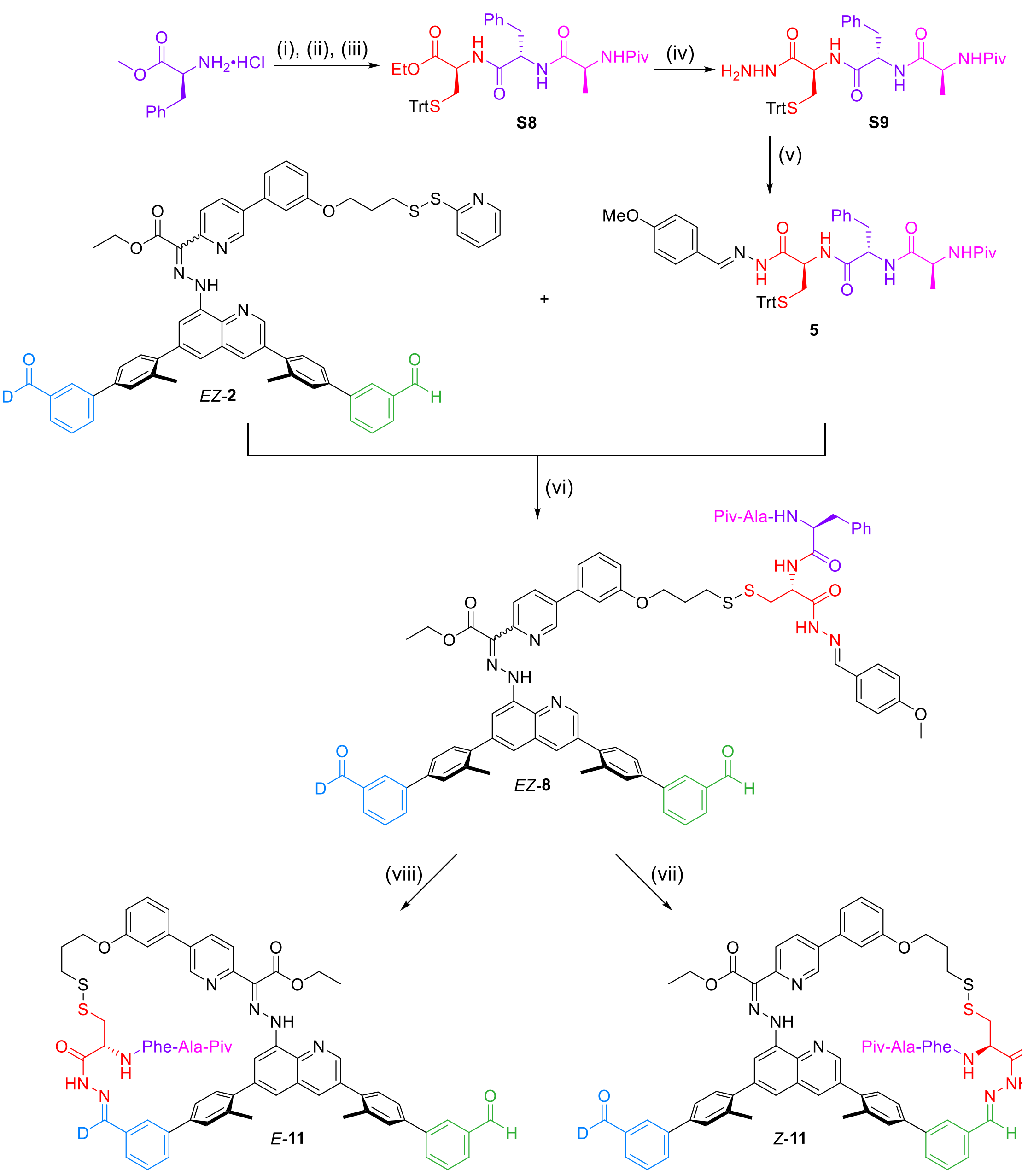

Scheme 3. Synthesis of *Z*-**11** and *E*-**11**. Reagents and conditions: (i) HO-Ala-Piv, HOBt·H_2O, EDCI·HCl, Et_3N, CH_2Cl_2, r.t., 18 h, 97%. (ii) LiOH, H_2O, THF, r.t., 2 h, 95%. (iii) **S1**, HOBt·H_2O, EDCI·HCl, CH_2Cl_2, r.t., 22 h, 91%. (iv) NH_2NH_2·H_2O, MeOH, Δ, 22 h, 50%. (v) *p*-anisaldehyde, AcOH, CH_2Cl_2, 30 °C, 23 h, 99%. (vi) $AgNO_3$, $CHCl_3$, MeOH, r.t., 60 mins, then *EZ*-**2** in $CHCl_3$, r.t., 27 h, 63%. (vii) TFA, $CHCl_3$, r.t., 23 h, 22%. (viii) TFA, $CHCl_3$, r.t., 47 h, 43%.

2.3.2 Synthetic Procedures and Characterisation Data

Synthesis of **S6**

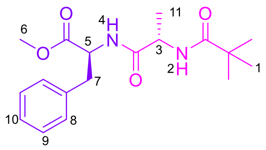

To a solution of L-phenylalanine methyl ester hydrochloride (654 mg, 3.03 mmol) in CH_2Cl_2 (60 mL) was added triethylamine (500 µL, 3.60 mmol,) HOBt·H_2O (489 mg, 3.62 mmol), EDCI·HCl (700 mg, 3.65 mmol) and Pivaloylalanine (624 mg, 3.60 mmol). The reaction mixture was stirred at room temperature for 18 hours. The reaction mixture was diluted with CH_2Cl_2, washed with a saturated aqueous solution of $NaHCO_3$ and brine. The organic layer was dried over $MgSO_4$, filtered and concentrated under reduced pressure. Flash column chromatography (SiO_2, MeOH/CH_2Cl_2, 3% → 6%) afforded **S6** as a colourless solid (980 mg, 2.93 mmol, 97%). **M.p.** 69 °C. **^{1}H NMR** (600 MHz, $CDCl_3$) δ 7.28 (t, *J* = 7.3 Hz, 2H, H_9), 7.23 (t, *J* = 7.3 Hz, 1H, H_{10}), 7.11 (d, *J* = 7.3 Hz, 2H, H_8), 6.74 (d, *J* = 7.7 Hz, 1H, H_2), 6.23 (d, *J* = 7.1 Hz, 1H, H_4), 4.81 (q, *J* = 6.7 Hz, 1H, H_5), 4.48 (quint, *J* = 7.1 Hz, 1H, H_3), 3.71 (s, 3H, H_6), 3.15 (dd, *J* = 13.9, 5.8 Hz, 1H, H_7), 3.07 (dd, *J* = 14.0, 6.6 Hz, 1H, $H_{7'}$), 1.34 (d, *J* = 7.0 Hz, 3H, H_{11}), 1.17 (s, 9H, H_1). **^{13}C NMR** (151 MHz, $CDCl_3$) δ 178.3, 172.2, 171.7, 135.8, 129.2, 128.6, 127.2, 53.4, 52.4, 48.5, 38.6, 37.8, 27.4, 18.4. **HRMS** (NSI$^+$): Calc. for $C_{18}H_{26}N_2O_4Na$: 357.1785, found 357.1767 $[M+Na]^+$.

Synthesis of **S7**

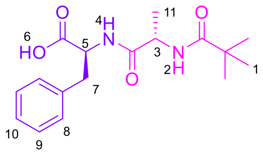

S6 (670 mg, 2.00 mmol) was dissolved in a 3:1 THF:LiOH (1 M) mixture (16 mL) and stirred at room temperature for 2 h. The reaction mixture was acidified with 1 M HCl (4.0 mL) and diluted with EtOAc. The two layers were separated and the aqueous layer extracted with EtOAc. The combined organic layers were washed with brine, dried with Na_2SO_4, filtered and concentrated under reduced pressure. Flash column chromatography (SiO_2, MeOH/CH_2Cl_2, 0% → 5%) afforded **S7** as a colourless solid (609 mg, 1.90 mmol, 95%). **M.p.** 98 °C. **^{1}H NMR** (600 MHz, $CDCl_3$) δ 7.25 (t, *J* = 7.3 Hz, 2H, H_9), 7.20 (t, *J* = 7.3 Hz, 1H, H_{10}), 7.13 (d, *J* = 7.3 Hz, 2H, H_8), 7.10 (d, *J* = 7.6 Hz, 1H, H_2), 6.48 (d, *J* = 7.6 Hz, 1H, H_4), 4.79 (q, *J* = 6.6 Hz, 1H, H_5), 4.56 (quint, *J* = 7.1 Hz, 1H, H_3), 3.17 (dd, *J* = 14.0, 5.6 Hz, 1H, H_7), 2.99 (dd, *J* = 14.0, 6.7 Hz, 1H, $H_{7'}$), 1.29 (d, *J* = 6.9 Hz, 3H, H_{11}), 1.17 (s, 9H, H_1). **^{13}C NMR** (151 MHz, $CDCl_3$) δ 178.9, 173.6, 172.3, 136.0, 129.4, 128.5, 127.0, 53.5, 48.6, 38.7, 37.6, 27.3, 18.3. **HRMS** (NSI$^+$): Calc. for $C_{17}H_{24}N_2O_4H$: 321.1809, found 321.1798 $[M+H]^+$.

Synthesis of **S8**

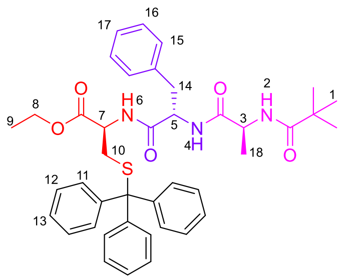

To a solution of **S1** (392 mg, 1.00 mmol) in CH_2Cl_2 (20 mL) was added HOBt·H_2O (162 mg, 1.20 mmol), EDCI·HCl (230 mg, 1.20 mmol) and **S7** (384 mg, 1.20 mmol). The reaction mixture was stirred at room temperature for 22 hours. The reaction mixture was diluted with CH_2Cl_2, washed with a saturated aqueous solution of $NaHCO_3$, a 1 M solution of HCl, and brine. The organic layer was dried over $MgSO_4$, filtered and concentrated under reduced pressure. Flash column chromatography (SiO_2, MeOH/CH_2Cl_2, 9%) afforded **S8** as a pale yellow solid (632 mg, 0.911 mmol, 91%). **M.p.** 104 °C. **^{1}H NMR** (600 MHz, $CDCl_3$) δ 7.33 (d, *J* = 7.3 Hz, 6H, H_{11}), 7.27 (t, *J* = 7.4 Hz, 6H, H_{12}), 7.22 – 7.20 (m, 7H, H_{13}+ H_{15}+ H_{16}), 7.14 – 7.11 (m, 1H, H_{17}), 6.74 (d, *J* = 5.6 Hz, 1H, H_4), 6.33 (d, *J* = 7.0 Hz, 1H, H_5), 6.17 (d, *J* = 6.7 Hz, 1H, H_2), 4.61 (q, *J* = 6.9 Hz, 1H, H_5), 4.44 – 4.39 (m, 2H, H_7+ H_3), 4.17 – 4.11 (m, 2H, H_8), 3.09 – 3.01 (m, 1H, H_5), 2.65 (dd, *J* = 12.6, 6.2 Hz, 1H, H_{14}), 2.53 (dd, *J* = 12.5, 5.0 Hz, 1H, $H_{14'}$), 1.31 (d, *J* = 7.0 Hz, 3H, H_{18}), 1.24 (t, *J* = 7.1 Hz, 3H, H_9), 1.14 (s, 9H, H_1). **^{13}C NMR** (151 MHz, $CDCl_3$) δ 178.5, 172.1, 170.2, 169.8, 144.2, 136.2, 129.5, 129.4, 128.7, 128.0, 127.0, 126.9, 66.9, 61.8, 54.1, 51.5, 48.8, 38.6, 38.0, 33.5, 27.4, 18.3, 14.1. **HRMS** (NSI$^+$): Calc. for $C_{41}H_{47}N_3O_5SH$: 694.3309, found 694.3280 $[M+H]^+$.

Synthesis of **S9**

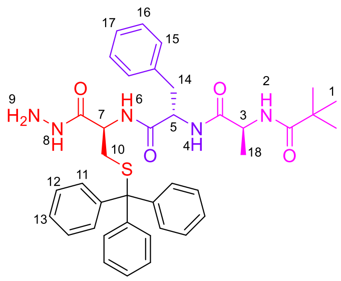

Hydrazine hydrate (42 µL, 0.864 mmol) was added to a solution of **S8** (173 mg, 0.249 mmol) in MeOH (3 mL) and the reaction mixture was stirred at reflux for 22 hours. The reaction mixture was allowed to cool to room temperature to precipitate the product which was isolated by filtration as a colourless solid (84.5 mg, 0.124 mmol, 50%). **^{1}H NMR** (600 MHz, $CDCl_3$) δ 7.91 (s, br, 1H, H_8), 7.38 (d, *J* = 7.2 Hz, 6H, H_{11}), 7.28 (t, *J* = 7.6 Hz, 6H, H_{12}), 7.23 – 7.16 (m, 6H, H_{13}+ H_{16}+ H_{17}), 7.12 (d, *J* = 7.7 Hz, 2H, H_{15}), 6.75 (d, *J* = 6.7 Hz, 1H, H_4), 6.61 (d, *J* = 8.3 Hz, 1H, H_6), 6.06 (d, *J* = 5.3 Hz, 1H, H_2), 4.52 (q, *J* = 6.6 Hz, 1H, H_5), 4.29 – 4.22 (m, 2H, H_7+ H_3), 3.74 (s, br, 2H, H_9), 3.16 (dd, *J* = 14.1, 6.1 Hz, 1H, H_{10}), 2.92 (dd, *J* = 14.0, 6.6 Hz, 1H, $H_{10'}$), 2.73 (dd, *J* = 13.1, 8.7 Hz, 1H, H_{14}), 2.65 (dd, *J* = 13.1, 5.1 Hz, 1H, $H_{14'}$), 1.30 (d, *J* = 7.0 Hz, 3H, H_{18}), 1.09 (s, 9H, H_1). **^{13}C NMR** (151 MHz, $CDCl_3$) δ 179.7, 172.9, 170.5, 169.9, 144.4, 135.6, 129.6, 129.3, 129.0, 128.0, 127.3, 126.9, 67.2, 54.4, 51.8, 50.0, 38.5, 37.3, 33.4, 27.3, 17.8. **HRMS** (NSI$^+$): Calc. for $C_{39}H_{45}N_5O_4SH$: 680.3265, found 680.3246 $[M+H]^+$.

Synthesis of **5**

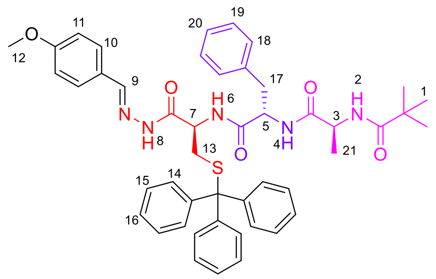

S9 (75.0 mg, 0.110 mmo), *p*-anisaldehyde (27 µL, 0.220 mmol) and acetic acid (2 drops) were combined in CH_2Cl_2 (2 mL) and the reaction mixture was stirred at 30 °C for 23 hours. The reaction mixture was concentrated under reduced pressure and purified by flash column chromatography (SiO_2, MeOH/CH_2Cl_2, 0% → 3%) to afford **5** as a colourless film (87.1 mg, 0.109 mmol, 99%). **^{1}H NMR** (600 MHz, $CDCl_3$) ~32:68 *cis*:*trans* hydrazone amide rotamers. *Trans*-**5** δ 10.13 (s, 1H, H_8), 8.18 (s, 1H, H_9), 7.66 (d, J = 8.8 Hz, 2H, H_{10}), 7.38 (d, J = 7.6 Hz, 6H, H_{14}), 7.28 – 7.07 (m, 14H, H_{15}+ H_{16}+ H_{18}+ H_{19}+ H_{20}), 7.01 (d, J = 7.9 Hz, 1H, H_4), 6.88 (d, J = 8.8 Hz, 2H, H_{11}), 6.80 – 6.77 (m, 1H, H_6), 6.20 (s, br, 1H, H_2), 4.59 – 4.56 (m, 2H, H_{17}), 4.50 – 4.45 (m, 1H, H_7), 4.35 (s, br, 1H, H_3), 3.82 (s, 3H, H_{12}), 3.07 – 2.95 (m, 1H, H_5), 2.79 – 2.73 (m, 2H, H_{13}), 1.28 (d, J = 7.1 Hz, 3H, H_{21}), 1.11 (s, 9H, H_1). *Cis*-**5** δ 9.27 (s, 1H, H_8), 7.62 (s, 1H, H_9), 7.46 (d, J = 8.8 Hz, 2H, H_{10}), 7.31 (d, J = 7.3 Hz, 6H, H_{14}), 7.28 – 7.07 (m, 14H, H_{15}+ H_{16}+ H_{18}+ H_{19}+ H_{20}), 7.01 (d, J = 7.9 Hz, 1H, H_4), 6.90 (d, J = 8.7 Hz, 2H, H_{11}), 6.80 – 6.77 (m, 1H, H_6), 6.27 (d, J = 7.3 Hz, 1H, H_2), 5.37 – 5.34 (m, 1H, H_7), 4.73 – 4.69 (m, 2H, H_{17}), 4.50 – 4.45 (m, 1H, H_3), 3.87 (s, 3H, H_{12}), 3.14 – 3.11 (m, 1H, H_5), 3.07 – 2.95 (m, 1H, H_{13}), 2.61 – 2.58 (m, 1H, $H_{13'}$), 1.31 (d, J = 7.0 Hz, 3H, H_{21}), 1.15 (s, 9H, H_1). **^{13}C NMR** (151 MHz, $CDCl_3$) δ 179.6, 178.4, 173.1, 172.5, 170.8, 170.4, 166.0, 161.6, 149.1, 145.1, 144.6, 144.5, 136.5, 135.8, 129.7, 129.6, 129.5, 129.2, 129.1, 128.2, 128.0, 127.4, 127.1, 127.0, 126.8, 126.7, 126.1, 114.4, 114.2, 67.3, 66.8, 55.6, 55.5, 54.8, 52.2, 50.0, 48.8, 38.7, 38.5, 36.8, 33.9, 33.7, 27.6, 27.5, 18.7, 18.2. **HRMS** (NSI^+): Calc. for $C_{47}H_{51}N_5O_5SNa$: 820.3503, found 820.3476 $[M+Na]^+$.

<u>Synthesis of *EZ*-**8**</u>

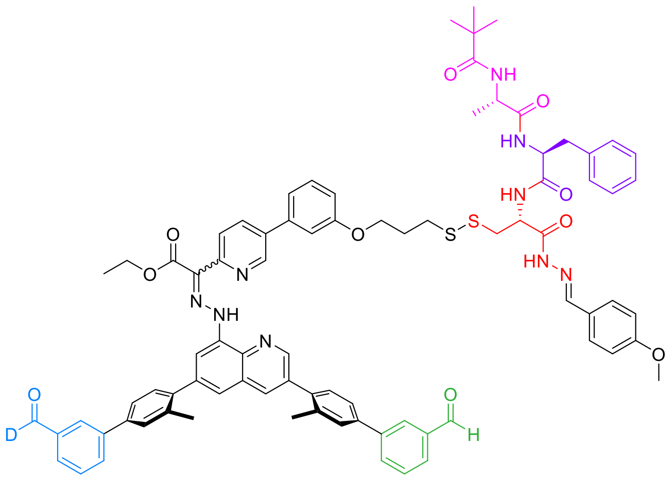

$AgNO_3$ (6.1 mg, 36.0 µmol) was added to a solution of **5** (23.9 mg, 30.0 µmol) in 3:1 $CHCl_3$:MeOH mixture (800 µL) and the mixture was stirred at room temperature for 1 hour. A solution of *EZ*-**2** (44.3 mg, 45.0 µmol) in $CHCl_3$ (450 µL) was added and the reaction was stirred at room temperature for 27 hours. The reaction mixture was diluted with $CHCl_3$, washed with brine, dried over Na_2SO_4, filtered and concentrated under reduced pressure. Preparative thin layer chromatography (SiO_2, EtOAc/CH_2Cl_2 (0.1% Et_3N), 40%) afforded *EZ*-**8** as a yellow solid (27.0 mg, 18.9 µmol, 63%). **^{1}H NMR** (600 MHz, $CDCl_3$) ~70:30 *E*:*Z* pyridyl-hydrazone isomers and mixture of acyl-hydrazone rotamers. δ 16.23 (s, 1H), 13.47 (s, br, 1H), 10.15

(s, 1H), 10.03 (s, br, 1H), 9.18 – 9.14 (m, 1H), 9.05 – 8.97 (m, 2H), 8.34 – 8.28 (m, 2H), 8.24 – 8.15 (m, 4H), 8.12 – 8.10 (m, 1H), 8.08 – 8.03 (dt, *J* = 8.7, 1.8 Hz, 1H), 8.00 – 7.90 (m, 5H), 7.84 – 7.78 (m, 2H), 7.74 – 7.56 (m, 16H), 7.55 – 7.40 (m, 12H), 7.34 – 7.30 (m, 4H), 7.27 – 7.18 (m, 9H), 7.02 – 6.89 (m, 6H), 6.36 (s, br, 1H), 5.93 (dd, *J* = 15.4, 2.8 Hz, 1H), 5.08 – 5.03 (m, 1H), 5.06 (t, *J* = 9.9 Hz, 1H), 4.62 (q, *J* = 6.2 Hz, 1H), 4.59 – 4.54 (m, 1H), 4.46 (q, *J* = 6.8 Hz, 2H), 4.23 – 4.02 (m, 6H), 3.84 (d, *J* = 2.5 Hz, 4H), 3.54 (dt, *J* = 13.8, 3.5 Hz, 1H), 3.43 – 3.38 (m, 13H), 3.20 – 3.06 (m, 4H), 3.01 – 2.97 (m, 3H), 2.87 (s, 5H), 2.54 – 2.46 (m, 10H), 2.40 (t, *J* = 8.1 Hz, 3H), 2.30 – 2.23 (m, 3H), 2.04 (quint, *J* = 8.1 Hz, 4H), 1.48 (t, *J* = 6.9 Hz, 3H), 1.04 – 1.02 (m, 11H).

Synthesis of *E*-**11**

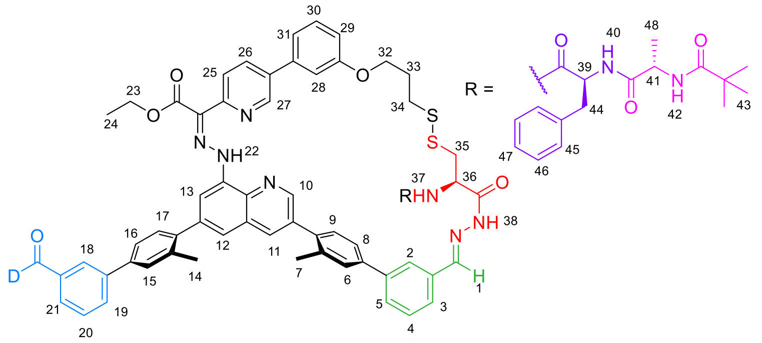

EZ-**8** (8.0 mg, 5.6 µmol) and trifluoroacetic acid (30 µL, 0.392 mmol) were dissolved in $CHCl_3$ (5.6 mL) and stirred at room temperature for 23 hours. Triethylamine (109 µL, 0.784 mmol) was added and the mixture was washed with a saturated aqueous solution of $NaHCO_3$. The aqueous layer was extracted with CH_2Cl_2 and the combined organic layers were dried over Na_2SO_4, filtered and concentrated under reduced pressure. Preparative thin layer chromatography (SiO_2, EtOAc/CH_2Cl_2 (0.2% Et_3N), 50%) afforded *E*-**11** as a yellow solid (1.6 mg, 1.2 µmol, 22%) and *Z*-**11** as a yellow solid (1.2 mg, 0.93 µmol, 17%). **^{1}H NMR** (600 MHz, $CDCl_3$) ~88:12 *cis*:*trans* hydrazone amide rotamers, major rotamer reported. δ 16.28 (s, 1H, H_{22}), 9.93 (s, 1H, H_{38}), 9.20 (s, 1H, H_{27}), 9.05 (s, 1H, H_{10}), 8.31 (d, *J* = 8.4 Hz, 1H, H_{25}), 8.25 (s, 1H, H_{11}), 8.22 (s, 2H, H_2+ H_{18}), 8.09 (d, *J* = 9.6 Hz, 1H, H_{26}), 8.03 (d, *J* = 1.3 Hz, 1H, H_{13}), 7.99 (s, 1H, H_1), 7.97 (d, *J* = 7.8 Hz, 1H, H_{19}), 7.91 (d, *J* = 7.2 Hz, 1H, H_5), 7.72 (d, *J* = 7.2 Hz, 1H, H_3), 7.70 – 7.66 (m, 3H, H_6+ H_8+ H_{15}), 7.63 – 7.62 (m, 1H, H_{20}), 7.59 – 7.53 (m, 4H, H_4+ H_9+ H_{16}+ H_{17}), 7.49 (d, *J* = 1.3 Hz, 1H, H_{12}), 7.46 – 7.37 (m, 4H, H_{21}+ H_{30}+ H_{46}), 7.28 – 7.26 (m, 5H, H_{28}+ H_{31}+ H_{45}+ H_{47}), 7.25 – 7.24 (m, 1H, H_{37}), 7.22 – 7.21 (m, 1H, H_{40}), 6.92 (d, *J* = 7.2 Hz, 1H, H_{29}), 6.13 (d, *J* = 7.8 Hz, 1H, H_{42}), 5.73 (q, *J* = 6.6 Hz, 1H, H_{36}), 4.84 (q, *J* = 7.2 Hz, 1H, H_{39}), 4.49 (quint., *J* = 6.6 Hz, 1H, H_{41}), 4.44 (q, *J* = 6.6 Hz, 1H, H_{23}), 4.18 – 4.12 (m, 2H, H_{32}), 3.56 (dd, *J* = 13.2, 4.2 Hz, 1H, H_{35}), 3.25 – 3.17 (m, 2H, $H_{35'}$+ H_{44}), 3.16 – 3.06 (m, 1H, $H_{44'}$), 3.10 – 2.85 (m, 2H, H_{34}), 2.52 (s, 3H, H_7), 2.51 (s, 3H, H_{14}), 2.33 – 2.21 (m, 2H, H_{33}), 1.46 (t, *J* = 7.2 Hz, 3H, H_{24}), 1.32 (d, *J* = 7.2 Hz, 3H, H_{48}), 1.14 (s, 9H, H_{43}). **^{13}C NMR** (151 MHz, $CDCl_3$) δ 178.6, 172.3, 170.9, 170.5, 165.8, 159.5, 151.1, 150.3, 149.9, 148.4, 145.8, 145.2, 142.9, 141.9, 141.8, 141.7, 141.3, 140.6, 139.2, 138.9, 138.3, 137.5, 136.9, 136.5, 136.5, 136.4, 136.2, 135.1, 134.7, 134.6, 134.5, 133.7, 133.1, 130.9, 130.7, 130.4, 129.8, 129.6, 129.4, 129.3, 129.2, 128.8, 128.6, 128.3, 128.1, 127.7, 126.9,

126.1, 125.3, 125.2, 124.5, 123.7, 122.6, 120.4, 119.6, 115.1, 111.9, 66.2, 61.2, 54.7, 51.0, 48.7, 45.7, 38.6, 38.3, 34.6, 31.9, 31.6, 30.3, 30.1, 29.7, 29.4, 28.6, 27.4, 27.2, 22.7, 20.9, 18.1, 14.51. **HRMS** (ESI^+): Calc. for $C_{75}H_{72}DN_9O_8S_2Na$: 1315.4978, found 1315.3048 $[M+Na]^+$. **HRMS** (ESI^-): Calc. for $C_{75}H_{71}DN_9O_8S_2$: 1291.5014, found 1291.3281 $[M-H]^-$.

<u>Synthesis of *Z*-**11**</u>

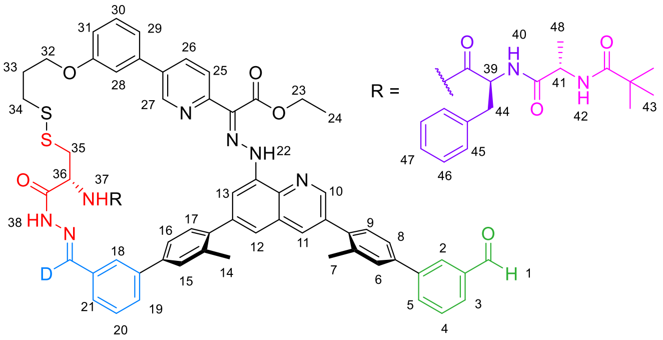

EZ-**8** (8.0 mg, 5.6 µmol) and trifluoroacetic acid (1 µL, 17 µmol) were dissolved in $CHCl_3$ (2.2 mL) and stirred at room temperature for 47 hours. Triethylamine (5 µL, 34 µmol) was added and the mixture was washed with a saturated aqueous solution of $NaHCO_3$. The aqueous layer was extracted with CH_2Cl_2 and the combined organic layers were dried over Na_2SO_4, filtered and concentrated under reduced pressure. Preparative thin layer chromatography (SiO_2, EtOAc/CH_2Cl_2 (0.2% Et_3N), 50%) afforded *Z*-**11** as a yellow solid (3.1 mg, 2.4 µmol, 43%). **^{1}H NMR** (600 MHz, $CDCl_3$) ~10:90 *cis*:*trans* hydrazone amide rotamers, major rotamer reported δ 13.28 (s, 1H, H_{22}), 10.26 (s, 1H, H_{38}), 10.13 (s, 1H, H_1), 8.98 (d, *J* = 1.9 Hz, 1H, H_{10}), 8.94 (d, *J* = 1.5 Hz, 1H, H_{27}), 8.47 (t, *J*= 1.8 Hz, 1H, H_{18}), 8.22 (t, *J* = 1.8 Hz, 1H, H_2), 8.19 (d, J = 1.9 Hz, 1H, H_{11}), 8.17 (dd, *J* = 8.2, 1.5 Hz, H_{26}), 8.01 (d, *J* = 8.2 Hz, 1H, H_{25}), 7.99 – 7.97 (m, 2H, H_3+ H_5), 7.90 (m, 1H, H_{19}), 7.71 – 7.66 (m, 3H, H_4+ H_{13}+ H_{20}), 7.64 (dd, *J* = 8.0, 1.6 Hz, 1H, H_{21}), 7.60 (s, 1H, H_6), 7.55 – 7.45 (m, 5H, H_8+ H_9+ H_{15}+ H_{29}+ H_{31}), 7.43 – 7.37 (m, 4H, H_{12}+ H_{16}+ H_{17}+ H_{47}), 7.34 – 7.31 (m, 3H, H_{46}+ H_{30}), 7.23 (d, *J* = 7.2 Hz, 2H, H_{45}), 6.92 (dd, *J* = 7.2, 1.8 Hz, 1H, H_{28}), 6.32 (s, 1H, H_{40}), 5.93 (s, 1H, H_{42}), 5.14 (ddd, *J* = 10.8, 8.4, 2.4 Hz, 1H, H_{36}), 4.64 (q, *J* = 6.0 Hz, 1H, H_{39}), 4.57 (q, *J* = 7.2 Hz, 2H, H_{23}), 4.19 – 4.08 (m, 2H, H_{32}), 4.07 – 4.00 (m, 1H, H_{41}), 3.67 – 3.59 (m, 1H, H_{35}), 3.48 (dd, *J* = 14.4, 4.8 Hz, 1H, H_{44}), 3.15 (dd, *J* = 13.2, 10.8 Hz, 1H, $H_{35'}$), 3.08 – 3.01 (m, 2H, H_{34}+ $H_{44'}$), 2.95 (ddd, *J* = 13.2, 10.2, 5.4 Hz, 1H, $H_{34'}$), 2.48 (s, 3H, H_7), 2.47 (s, 3H, H_{14}), 2.37 – 2.29 (m, 1H, H_{33}), 2.26 – 2.19 (m, 1H, $H_{33'}$), 1.50 – 1.43 (m, 6H, H_{24}+ H_{48}), 0.99 (s, 9H, H_{43}). **^{13}C NMR** (151 MHz, $CDCl_3$) δ 192.5, 181.4, 174.2, 171.2, 167.4, 163.3, 159.6, 149.5, 142.1, 141.8, 141.4, 140.8, 140.5, 139.7, 138.2, 137.1, 136.8, 135.9, 135.7, 135.5, 135.3, 135.2, 134.7, 133.2, 131.1, 130.3, 130.2, 130.2, 129.7, 129.6, 129.5, 129.0, 128.9, 128.8, 128.6, 128.3, 128.1, 127.9, 125.4, 125.1, 119.5, 114.4, 70.7, 66.5, 63.3, 61.8, 54.8, 52.2, 51.9, 38.9, 38.7, 36.1, 34.8, 33.6, 32.9, 32.1, 29.9, 29.5, 27.3, 25.9, 24.9, 22.8, 21.1, 20.9, 17.3, 14.4. **HRMS** (ESI^+): Calc. for $C_{75}H_{72}DN_9O_8S_2Na$: 1315.4978, found 1315.3048 $[M+Na]^+$. **HRMS** (ESI^-): Calc. for $C_{75}H_{71}DN_9O_8S_2$: 1291.5014, found 1291.3281 $[M-H]^-$.

3. Effect of Acid Stoichiometry on *E*-1/*Z*-1 Interconversion

3.1 Titration Procedure

To a solution of *E*-**1**/*Z*-**1** (1.22 mg, 1.25 µmol) in $C_2D_2Cl_4$ (500 µL, 2.5 mM) in a standard NMR tube was added trifluoroacetic acid and the sample was left to equilibrate at r.t. Triethylamine was then added and the reaction mixture was partitioned between CH_2Cl_2 and water. The layers were separated and washed with a saturated aqueous solution of ammonium chloride, water, a saturated aqueous solution of sodium bicarbonate, and brine. The organic layer was dried with Na_2SO_4, filtered and concentrated under reduced pressure. The residue was analysed by ^{1}H NMR in $C_2D_2Cl_4$ and compared to authentic samples of *E*-**1** and *Z*-**1** to determine the conversion of the operation (based on the integration of the hydrazone ester peaks).

3.2 Results

a

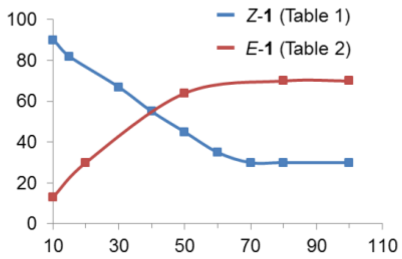

b

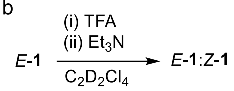

TFA (eq.)	*E*-**1**:*Z*-**1**
5	9:91
10	10:90
15	18:82
30	33:67
40	45:55
50	55:45
60	65:35
70	70:30
80	70:30
100	70:30

c

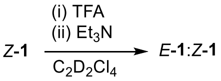

TFA (eq.)	*E*-**1**:*Z*-**1**
10	33:67
20	36:64
50	63:37
80	70:30
100	70:30

Figure 1. a. Graph showing the effect of acid stoichiometry on the *E*-**1**/*Z*-**1** interconversion (margin of error ±2%). b. *E*-**1** titration results. c. *Z*-**1** titration results.

4. Effect of Solvent on *E*-1/*Z*-1 Interconversion

4.1 Titration Procedure

To a solution of *Z*-**1** (1.22 mg, 1.25 µmol) in the adequate solvent (500 µL, 2.5 mM) in a standard NMR tube was added trifluoroacetic acid (8 µL, 80 eq.) and the sample was left to equilibrate at r.t. Triethylamine (16 µL, 100 eq.) was then added and the reaction mixture was partitioned between CH_2Cl_2 and water. The layers were separated and washed with a saturated aqueous solution of ammonium chloride, water, a saturated aqueous solution of sodium bicarbonate, and brine. The

organic layer was dried with Na_2SO_4, filtered and concentrated under reduced pressure. The residue was analysed by ^1H NMR in $C_2D_2Cl_4$ and compared to authentic samples of *E*-**1** and *Z*-**1** to determine the conversion of the operation (based on the integration of the hydrazone ester peaks).

4.2 Characterisation of *Z*-1-H_3^{3+}-Left and *Z*-1-H_3^{3+}-Right

4.2.1 *Z*-1-H_3^{3+}-Left in $C_2D_2Cl_4$:CD_3CN (2:1)

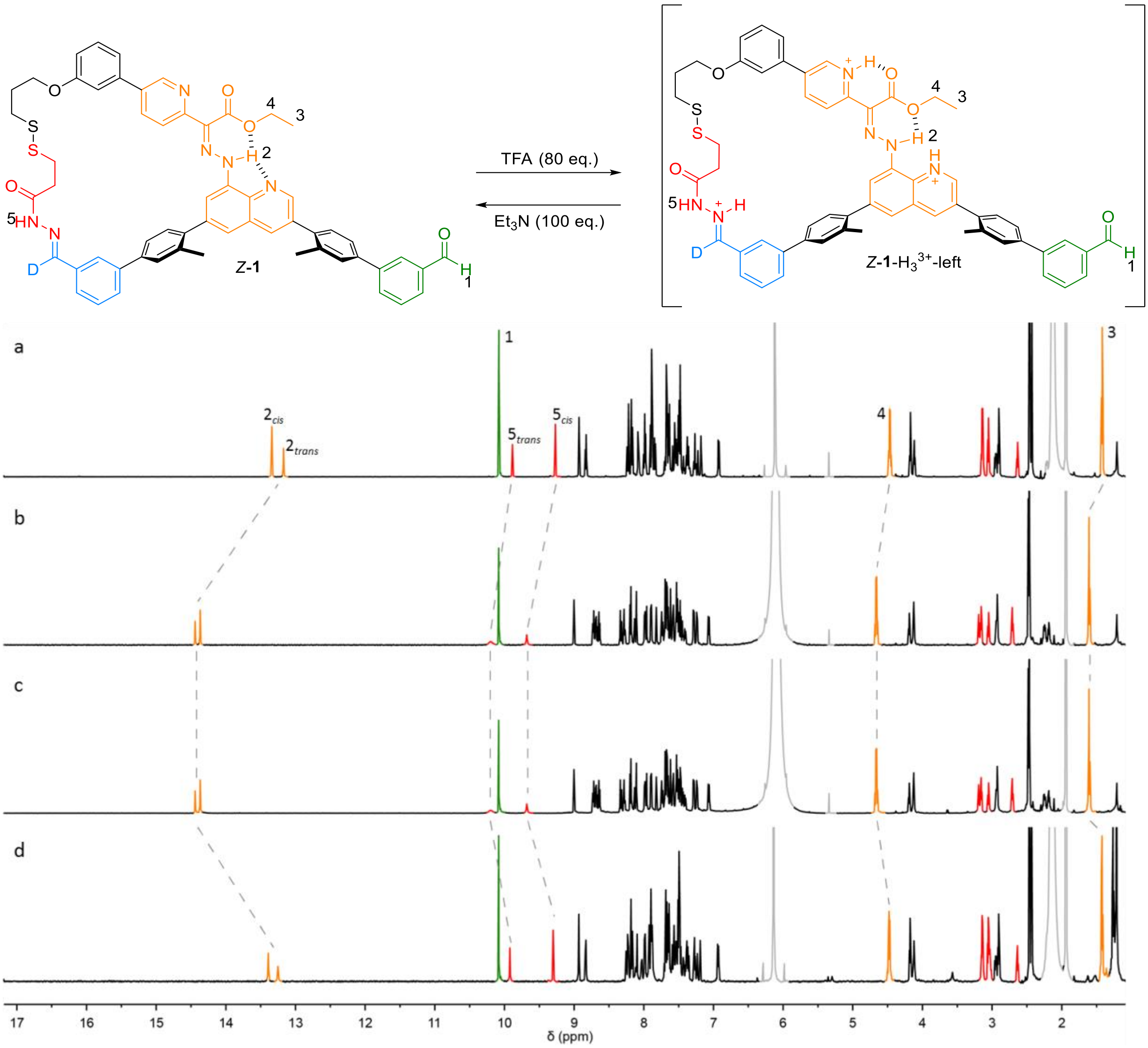

Figure 2. Partial ^1H NMR (600 MHz, 295 K, $C_2D_2Cl_4$:CD_3CN (2:1)) spectra of the transformation and intermediates observed upon the addition of 80 equivalents of TFA to *Z*-**1** in $C_2D_2Cl_4$:CD_3CN (as an example of polar solvent mixture). a) *Z*-**1** (mixture of acyl hydrazone rotamers); b) *Z*-**1**-H_3^{3+}-left 10 mins after the addition; c) *Z*-**1**-H_3^{3+}-left 12 h after the addition; d) sample after neutralisation and work-up (*Z*-**1**). Signals from traces of residual solvents are shown in grey. Dashed lines connect resonances indicative of the configuration of the hydrazone switch (H_2, H_3 and H_4) and the position of the cargo (H_1 and H_5).

2D ROESY spectrum of *Z*-1-H_3^{3+}-left in $C_2D_2Cl_4$:CD_3CN (2:1)

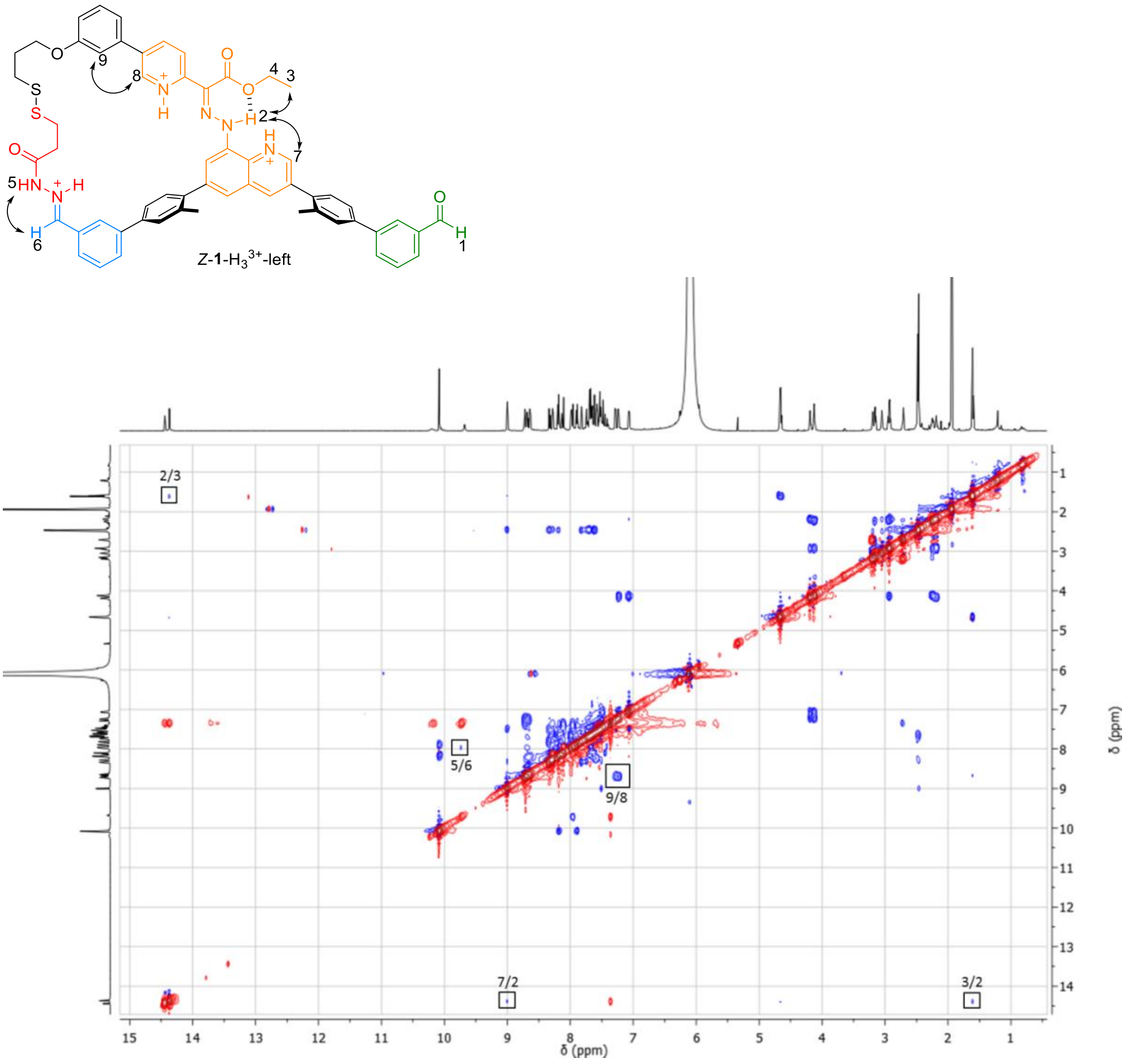

Figure 3. 2D ROESY NMR (600 MHz, 295 K, $C_2D_2Cl_4$:CD_3CN (2:1)) spectrum of *Z*-**1**-H_3^{3+}-left.

4.2.1 *Z*-1-H_3^{3+}-Right in $C_2D_2Cl_4$:$C_6D_5CD_3$ (1:2)

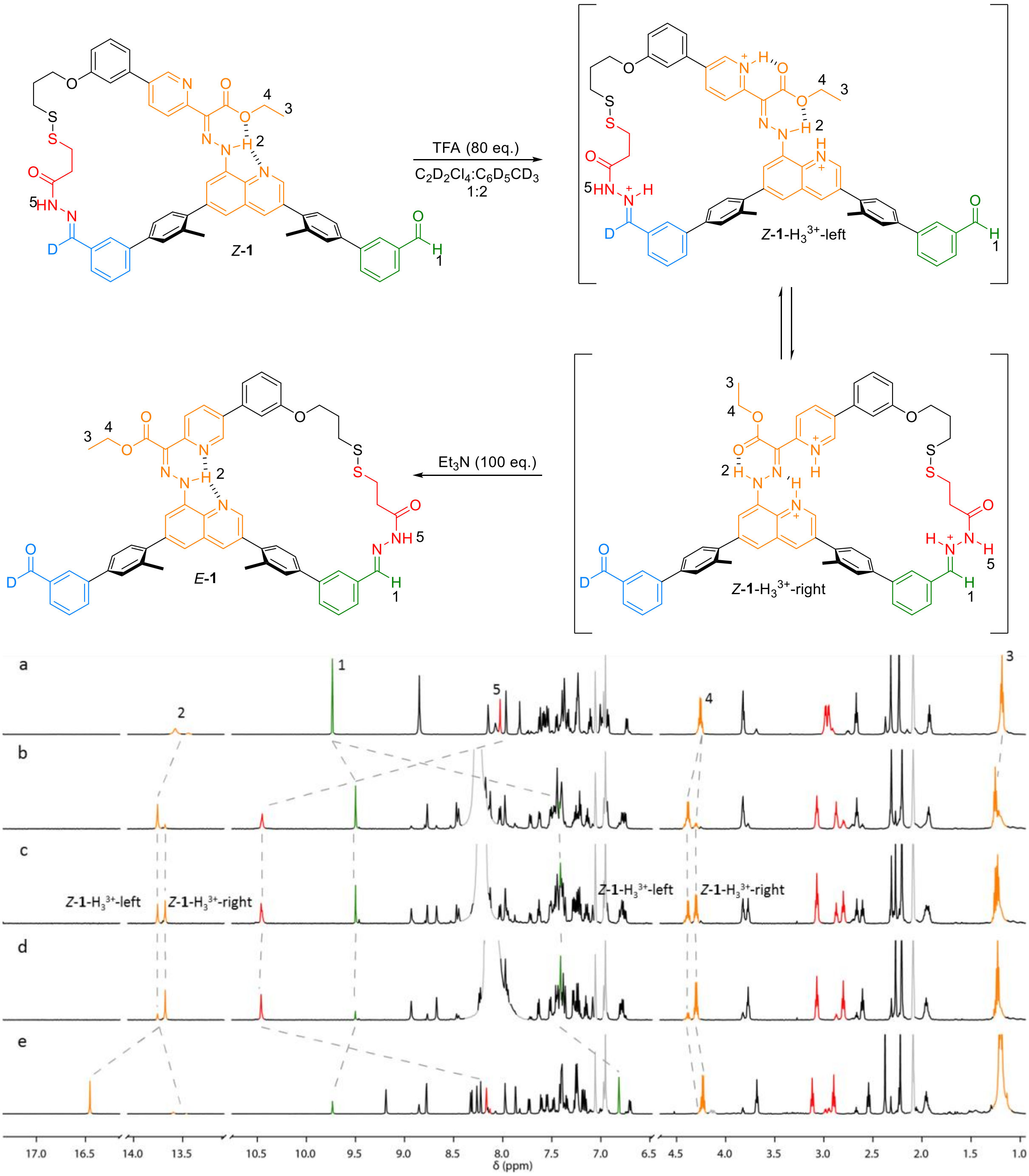

Figure 4. Partial ^{1}H NMR (600 MHz, 295 K, $C_2D_2Cl_4$:$C_6D_5CD_3$ (1:2)) spectra of the transformation and intermediates observed upon the addition of 80 equivalents of TFA to *Z*-**1** in $C_2D_2Cl_4$:$C_6D_5CD_3$ (as an example of unpolar solvent mixture). a) *Z*-**1** (mixture of acyl hydrazone rotamers); b) *Z*-**1**-H_3^{3+}-left: *Z*-**1**-H_3^{3+}-right (81:19) 12 mins after the addition; c) *Z*-**1**-H_3^{3+}-left: *Z*-**1**-H_3^{3+}-right (45:55) 1 h after the addition; d) *Z*-**1**-H_3^{3+}-left: *Z*-**1**-H_3^{3+}-right (17:83) 5 h after the addition; e) sample after neutralisation and work-up (*E*-**1**:*Z*-**1** 83:17). Signals from traces of residual solvents are shown in grey. Dashed lines connect resonances indicative of the configuration of the hydrazone switch (H_2, H_3 and H_4) and the position of the cargo (H_1 and H_5).

2D ROESY spectrum of *Z*-1-H_3^{3+}-right in $C_2D_2Cl_4$:$C_6D_5CD_3$ (2:1)

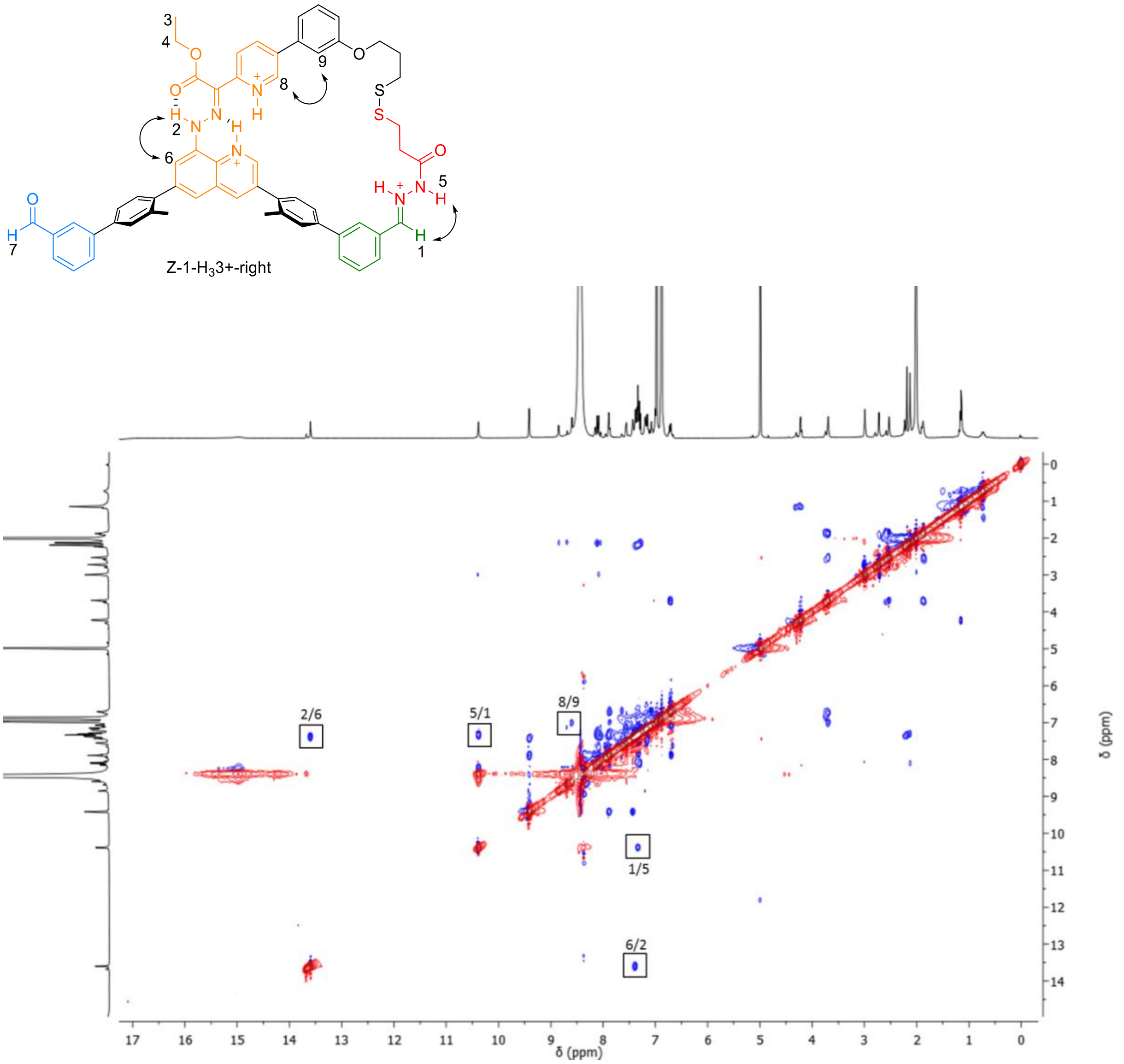

Figure 5. 2D ROESY NMR (600 MHz, 295 K, $C_2D_2Cl_4$:$C_6D_5CD_3$ (1:2)) spectrum of *Z*-**1**-H_3^{3+}-right

5. Optimised Backward Transport (*E*-1 to *Z*-1)

5.1 General Procedure

To a solution of *E*-**1** (1.22 mg, 1.25 µmol) in the $C_2D_2Cl_4$:CD_3CN (2:1, 500 µL, 2.5 mM) in a standard NMR tube was added trifluoroacetic acid (8 µL, 80 eq.) and the sample was left to equilibrate at r.t. Triethylamine (16 µL, 100 eq.) was then added and the reaction mixture was partitioned between CH_2Cl_2 and water. The layers were separated; the organic layer was washed with saturated aqueous ammonium chloride solution, water, saturated aqueous sodium bicarbonate solution, and brine. The organic layer was dried with Na_2SO_4, filtered and concentrated under reduced pressure. The residue was analysed by ^1H NMR in $C_2D_2Cl_4$ and compared to authentic samples of *E*-**1** and *Z*-**1** to determine the conversion of the operation (based on the integration of the hydrazone ester peaks).

5.2 ^{1}H NMR Spectra

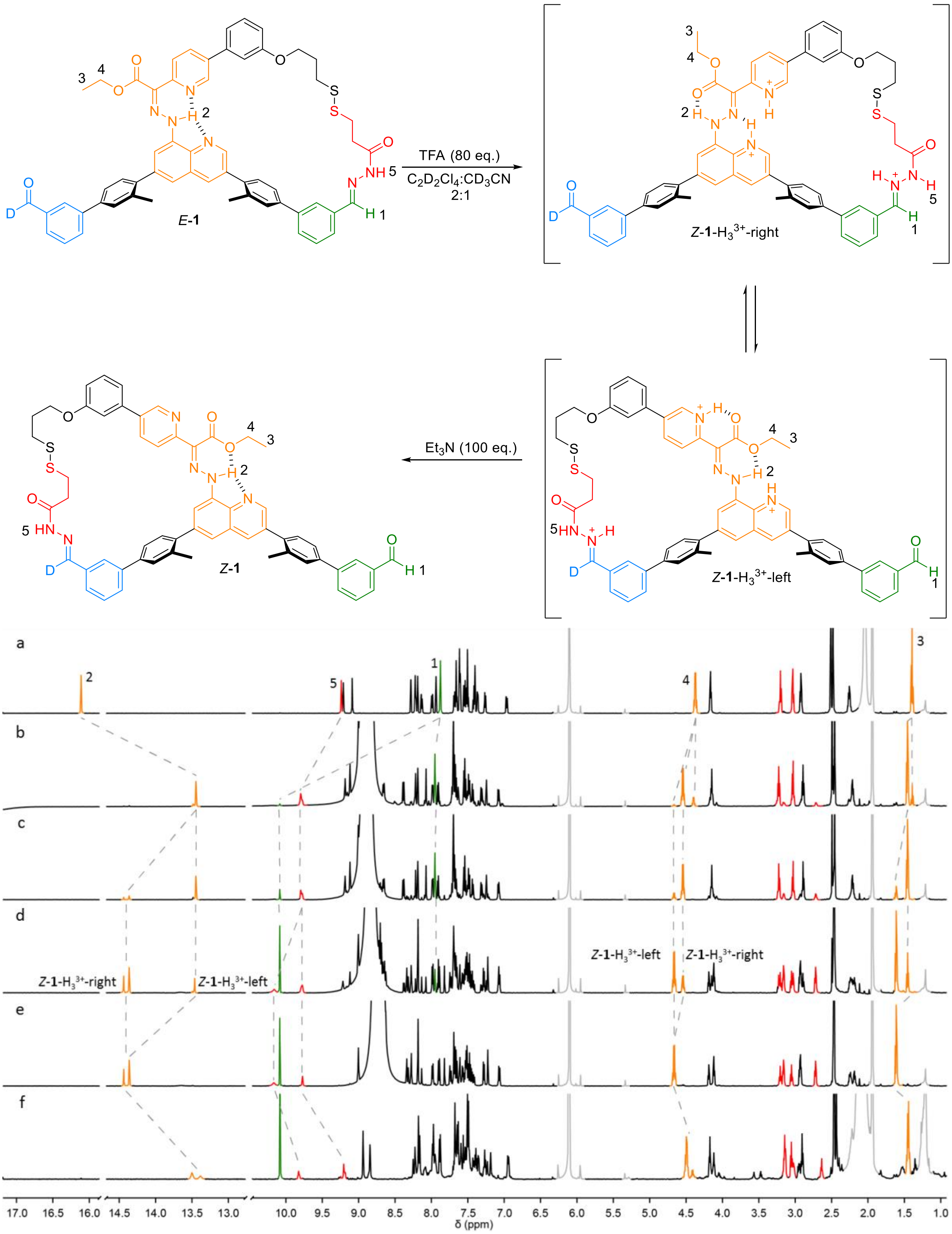

Figure 6. Partial ^{1}H NMR (600 MHz, 295 K, $C_2D_2Cl_4$:CD_3CN (2:1)) spectra of the conversion of *E*-**1** to *Z*-**1** with 80 equivalents of TFA. a) *E*-**1**; b) *Z*-**1**-H_3^{3+}-right:*E*-**1** (82:18) 10 mins after the addition; c) *Z*-**1**-H_3^{3+}-left: *Z*-**1**-H_3^{3+}-right (18:82) 1 h after the addition; d) *Z*-**1**-H_3^{3+}-left: *Z*-**1**-H_3^{3+}-right (72:28) 5 h after the addition; e) *Z*-**1**-H_3^{3+}-left: *Z*-**1**-H_3^{3+}-right (>98:2) 8 h after the addition; f) sample after neutralisation and work-up (*E*-**1**:*Z*-**1** >2:98). Signals from traces of residual solvents are shown in grey. Dashed lines connect resonances indicative of the configuration of the hydrazone switch (H_2, H_3 and H_4) and the position of the cargo (H_1 and H_5).

6. Site-to-Site Transport of Peptide Derivatives 9, 10 and 11

6.1 Forward Transport

6.1.1 General Procedure

To a solution of *Z*-**9**-**11** (1.125 µmol) in $C_2D_2Cl_4/C_6D_5CD_3$ (1:2, 450 µL, 2.5 mM) in a standard NMR tube was added trifluoroacetic acid (7 µL, 90 µmol, 80 eq.) and the sample (final volume: 450 µL, 2.5 mM) was left to equilibrate at r.t.. Triethylamine (16 µL, 113 µmol, 100 eq.) was then added and the reaction mixture was partitioned between CH_2Cl_2 and water. The layers were separated; the organic layer was washed with saturated aqueous ammonium chloride solution, water, saturated aqueous sodium bicarbonate solution, and brine. The organic layer was dried with Na_2SO_4, filtered and concentrated under reduced pressure. The residue was analysed by ^{1}H NMR in $CDCl_3$ and compared to authentic samples of *Z*-**9**-**11** and *E*-**9**-**11** to determine the conversion of the operation.

6.1.2 ^{1}H NMR Spectra

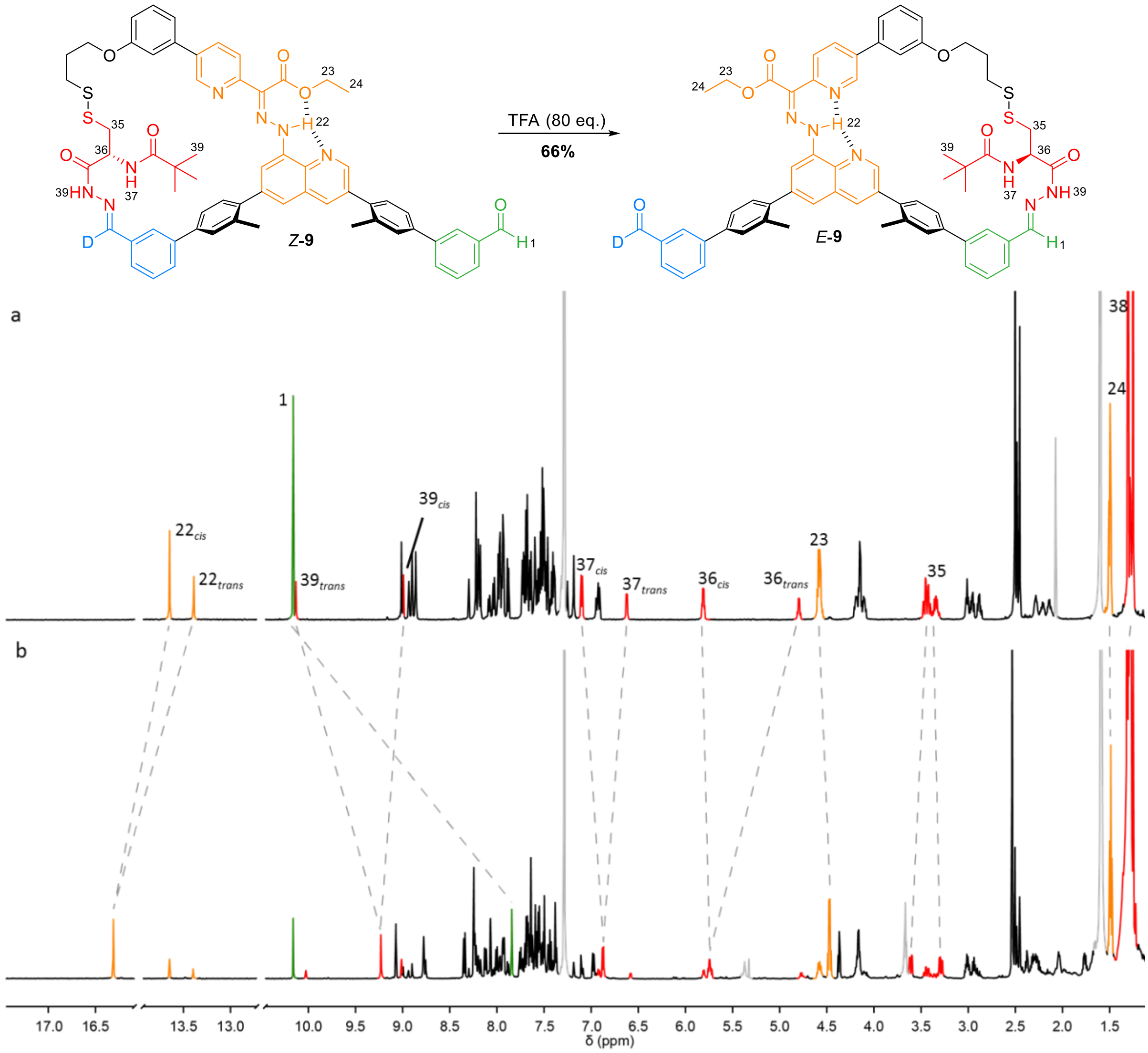

Figure 7. Partial ^{1}H NMR (600 MHz, 295 K, $CDCl_3$) spectra of the conversion of *Z*-**9** to *E*-**9** using the general procedure described above (**6.1.1**). a) *Z*-**9**; b) sample after neutralisation and work-up (*E*-**9**:*Z*-**9** 66:34). Signals from traces of residual solvents and organic salts are shown in grey. Dashed lines connect resonances belonging to the same proton.

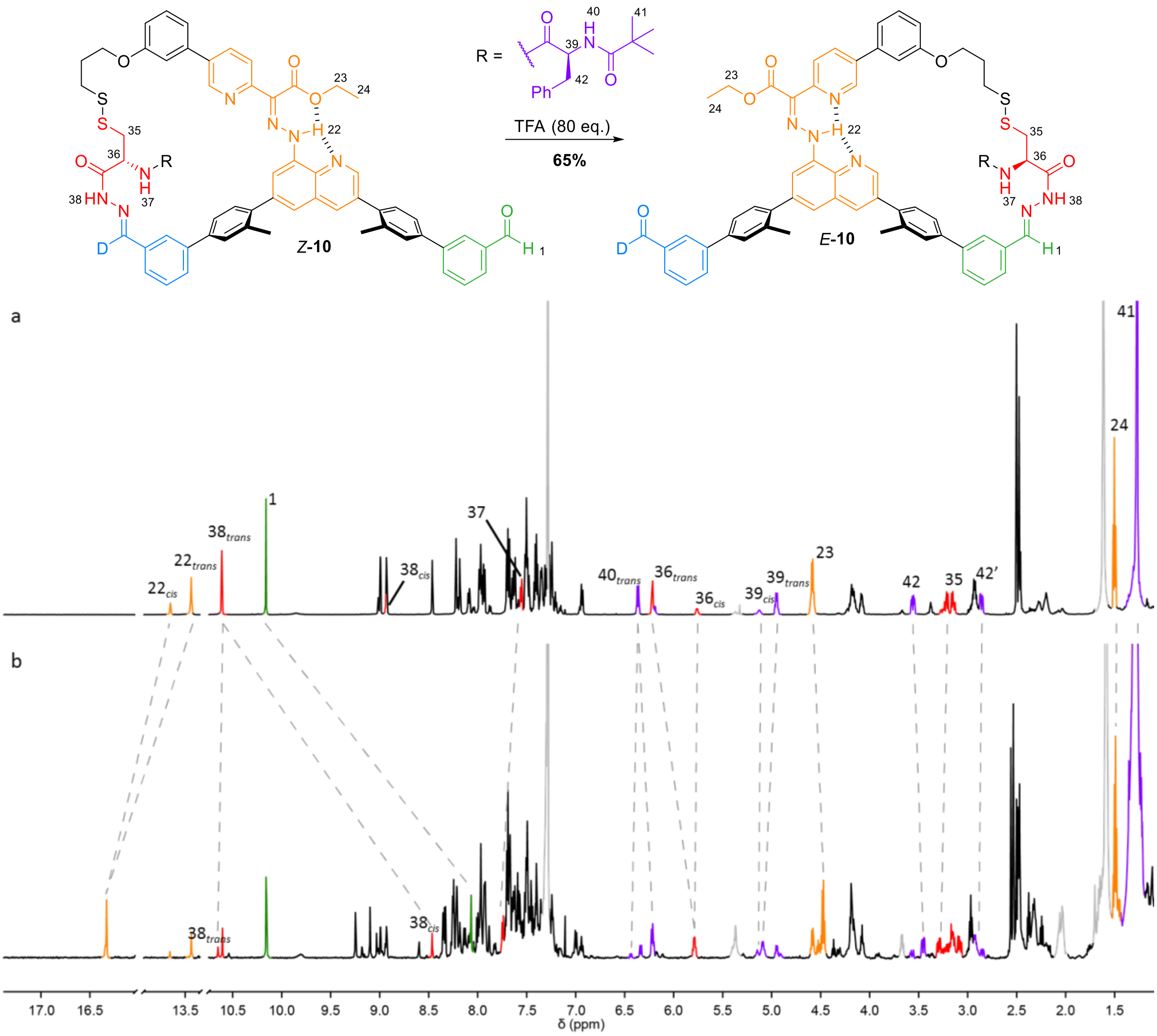

Figure 8. Partial ^{1}H NMR (600 MHz, 295 K, $CDCl_3$) spectra of the conversion of *Z*-**10** to *E*-**10** using the general procedure described above (**6.1.1**). a) *Z*-**10**; b) sample after neutralisation and work-up (*E*-**10**:*Z*-**10** 65:35). Signals from traces of residual solvents and organic salts are shown in grey. Dashed lines connect resonances belonging to the same proton.

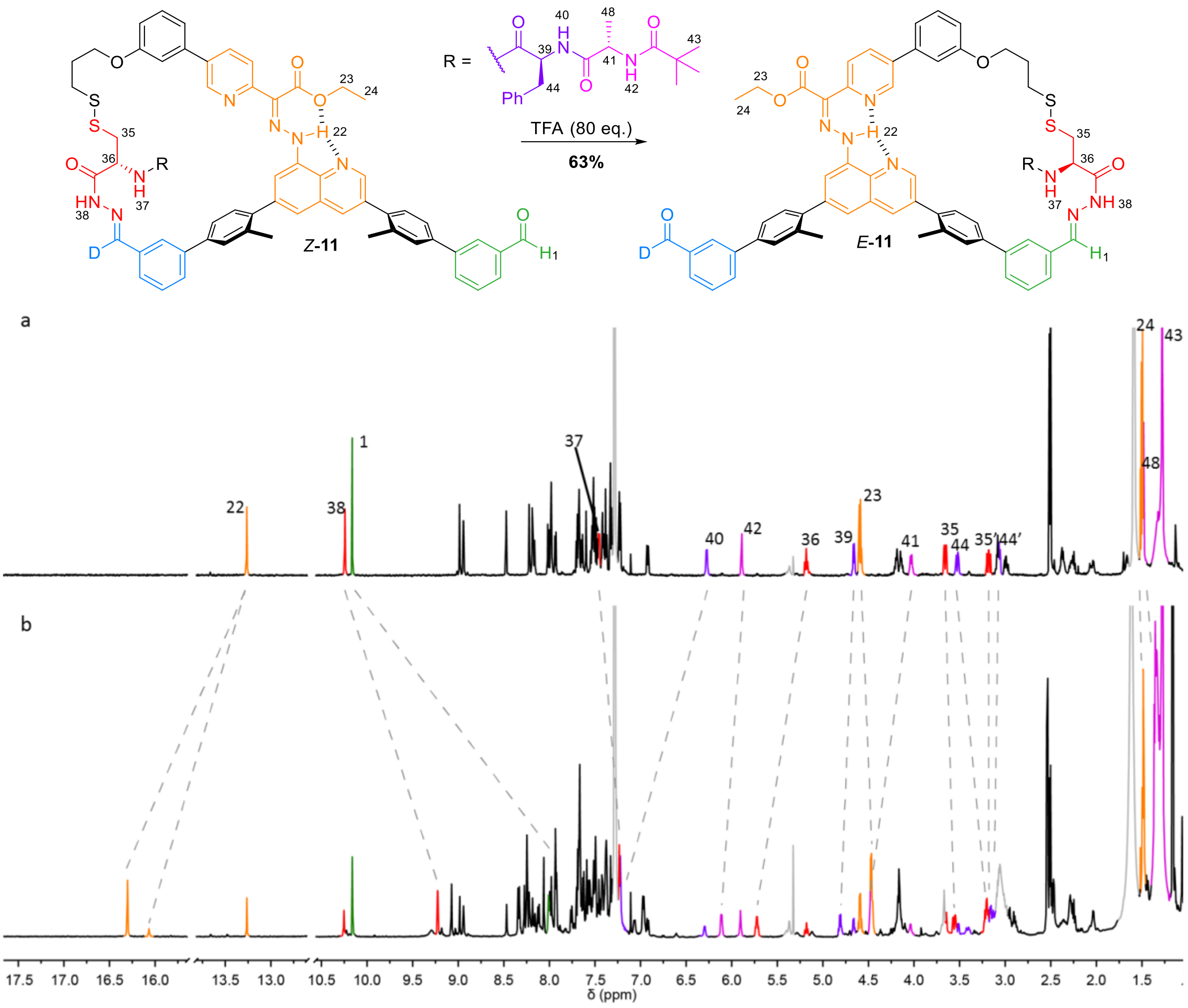

Figure 9. Partial ^{1}H NMR (600 MHz, 295 K, $CDCl_3$) spectra of the conversion of *Z*-**11** to *E*-**11** using the general procedure described above (**6.1.1**). a) *Z*-**11**; b) sample after neutralisation and work-up (*E*-**11**:*Z*-**11** 65:35). Signals from traces of residual solvents and organic salts are shown in grey. Dashed lines connect resonances belonging to the same proton.

6.2 Backward Transport

6.2.1 General Procedure

To a solution of *E*-**9**-**11** (1.125 µmol) in $C_2D_2Cl_4$:CD_3CN (2:1, 450 µL, 2.5 mM) in a standard NMR tube was added trifluoroacetic acid (7 µL, 90 µmol, 80 eq.) and the sample was left to equilibrate at r.t. Triethylamine (16 µL, 113 µmol, 100 eq.) was then added and the reaction mixture was partitioned between CH_2Cl_2 and water. The layers were separated; the organic layer was washed with saturated aqueous ammonium chloride solution, water, saturated aqueous sodium bicarbonate solution, and brine. The organic layer was dried with Na_2SO_4, filtered and concentrated under reduced pressure. The residue was analysed by ^{1}H NMR in $CDCl_3$ and compared to authentic samples of *Z*-**9**-**11** and *E*-**9**-**11** to determine the conversion of the operation.

6.2.2 ^{1}H NMR Spectra

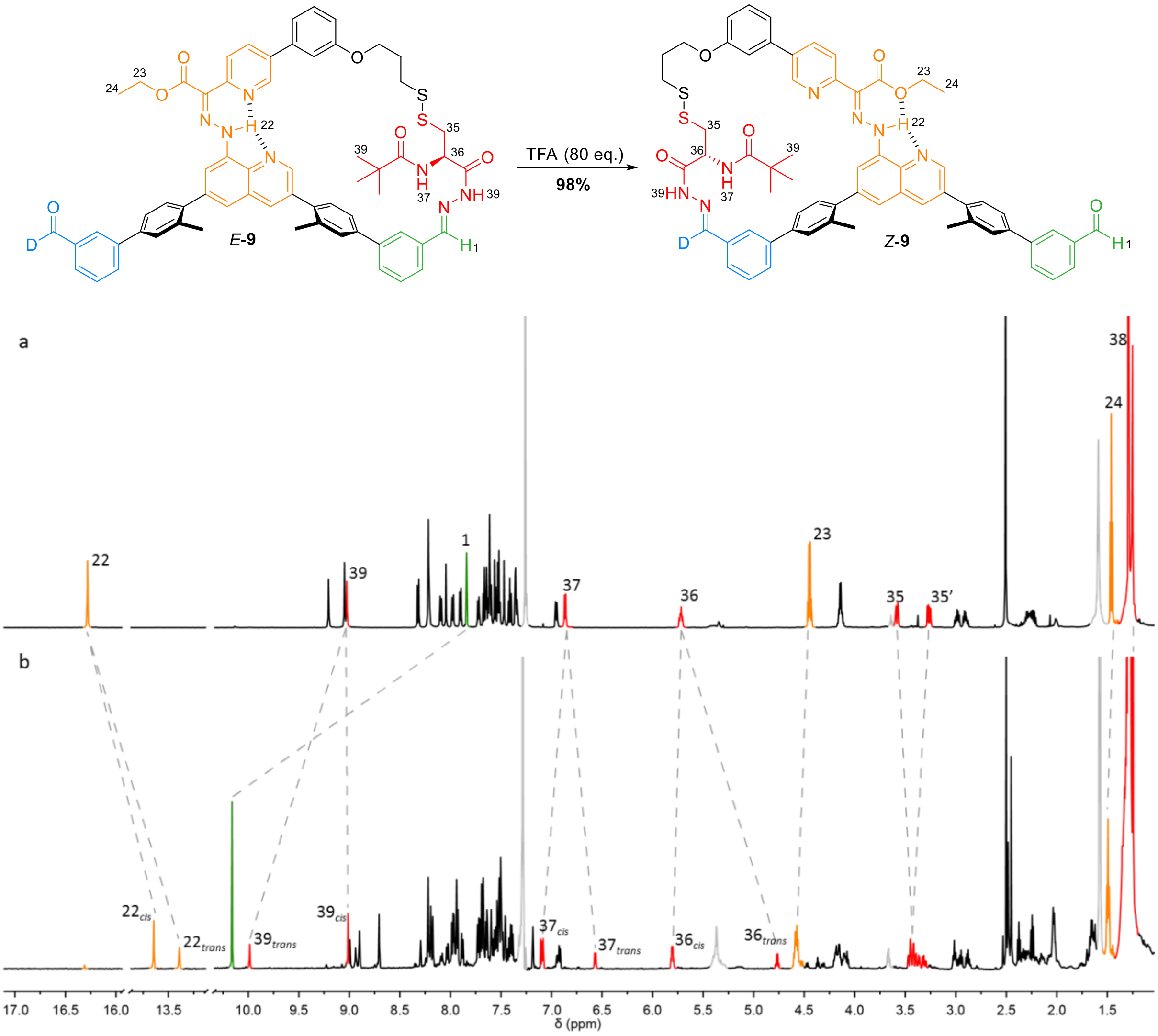

Figure 10. Partial ^{1}H NMR (600 MHz, 295 K, $CDCl_3$) spectra of the conversion of *E*-**9** to *Z*-**9** using the general procedure described above (**6.2.1**). a) *E*-**9**; b) sample after neutralisation and work-up (*E*-**9**:*Z*-**9** 2:98). Signals from traces of residual solvents and organic salts are shown in grey. Dashed lines connect resonances belonging to the same proton.

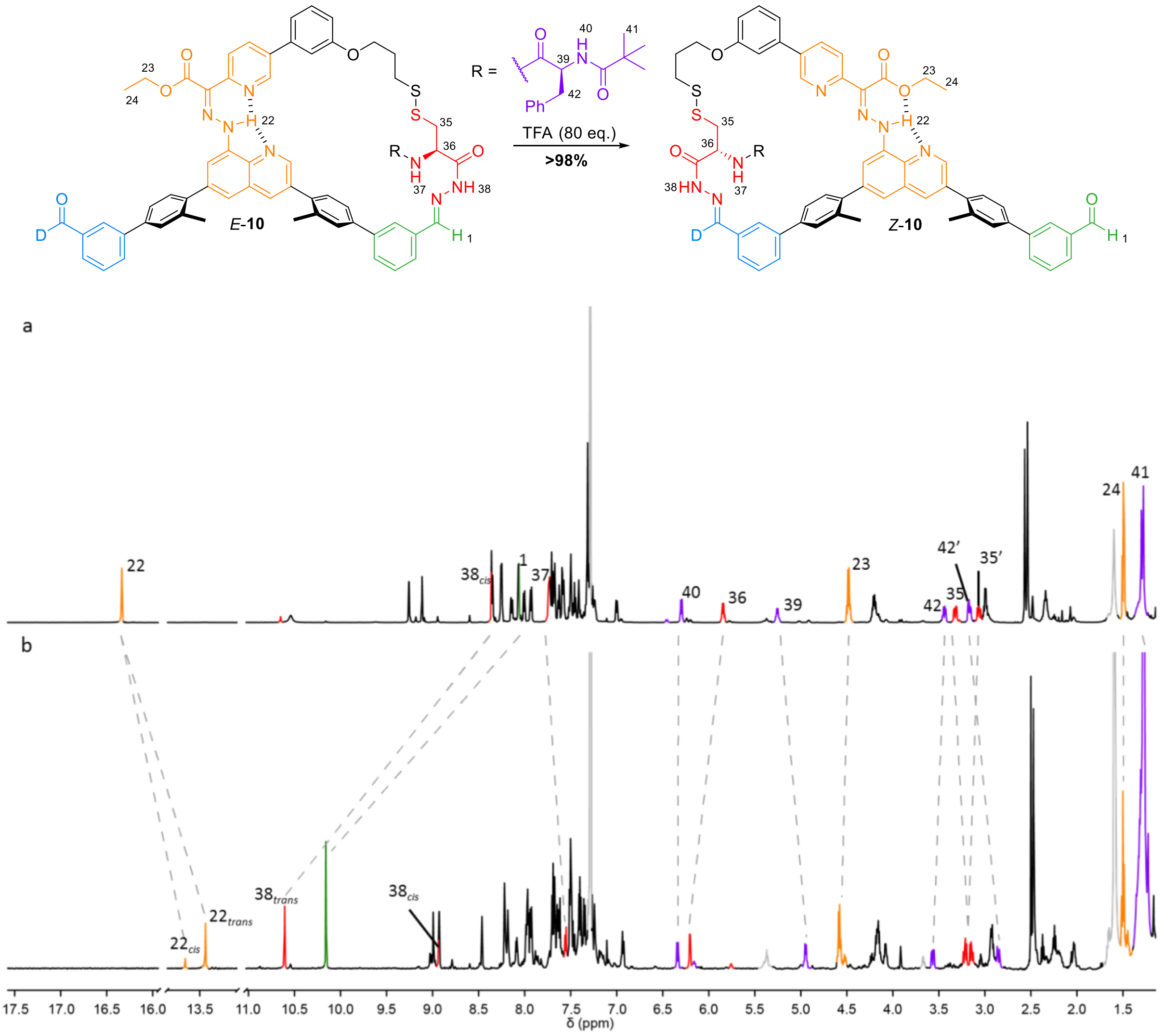

Figure 11. Partial ^{1}H NMR (600 MHz, 295 K, $CDCl_3$) spectra of the conversion of *E*-**10** to *Z*-**10** using the general procedure described above (**6.2.1**). a) *E*-**10**; b) sample after neutralisation and work-up (*E*-**10**:*Z*-**10** >2:98). Signals from traces of residual solvents and organic salts are shown in grey. Dashed lines connect resonances belonging to the same proton.

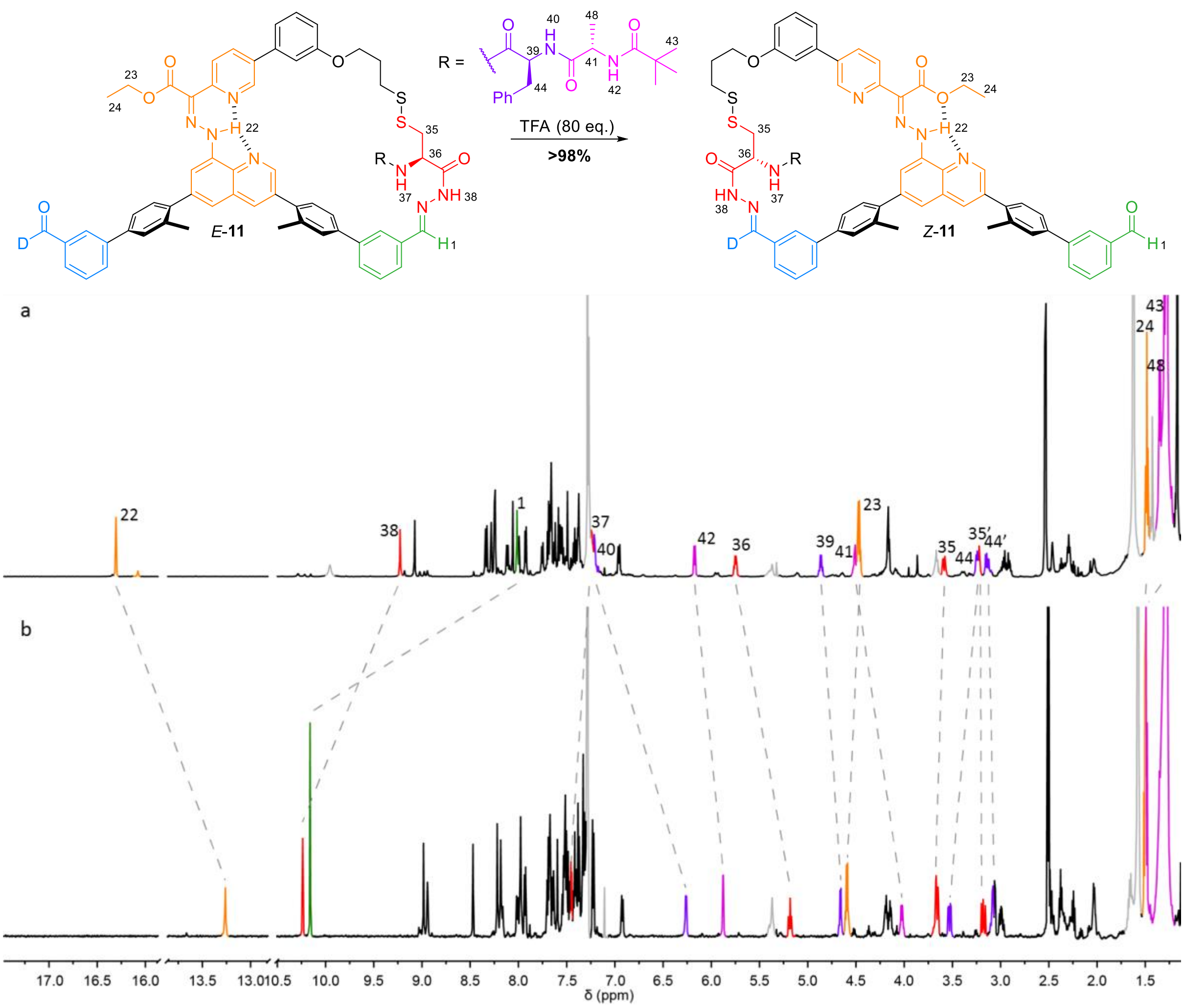

Figure 12. Partial ^{1}H NMR (600 MHz, 295 K, $CDCl_3$) spectra of the conversion of *E*-**11** to *Z*-**11** using the general procedure described above (**6.2.1**). a) *E*-**11**; b) sample after neutralisation and work-up (*E*-**11**:*Z*-**11** >2:98). Signals from traces of residual solvents and organic salts are shown in grey. Dashed lines connect resonances belonging to the same proton.

7. Spectroscopic Data

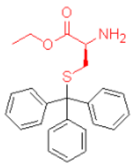

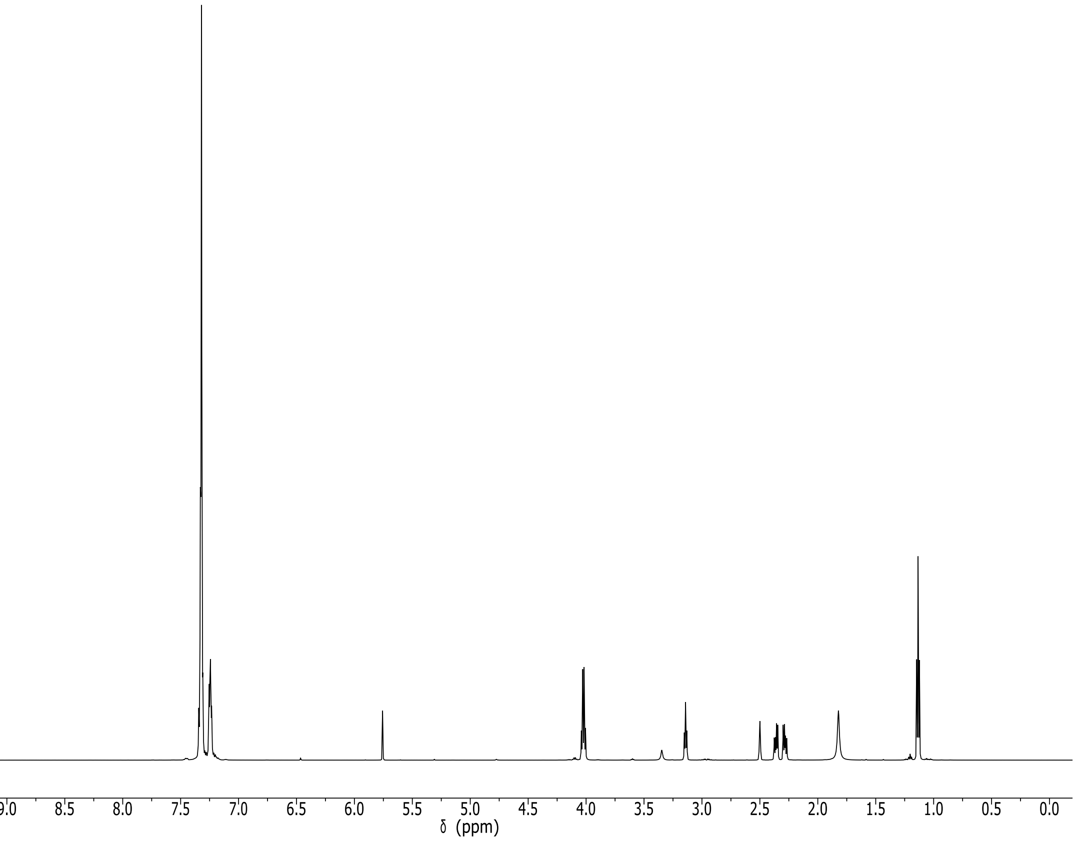

Spectrum 1. ^{1}H NMR (600 MHz, DMSO-d_6) of **S1**

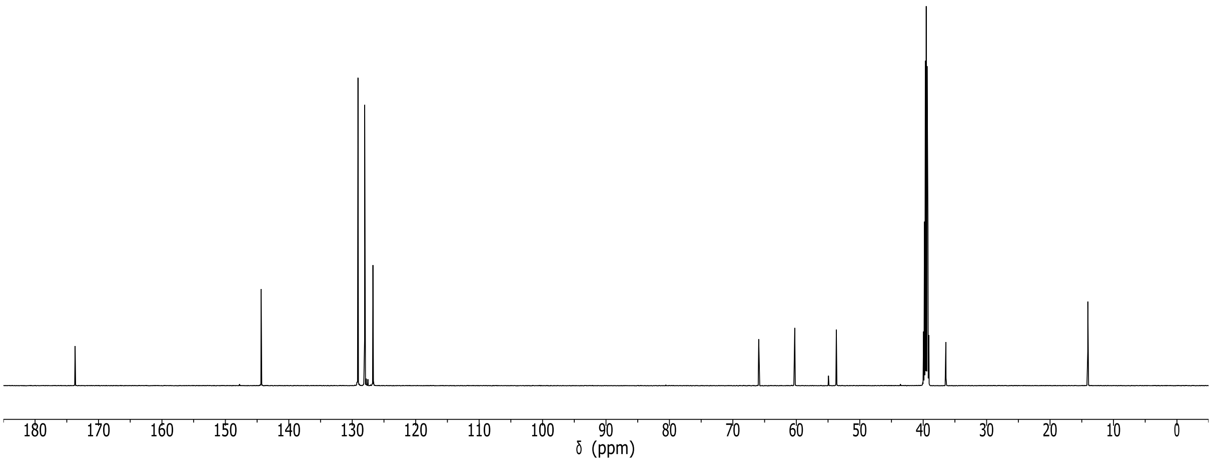

Spectrum 2. ^{13}C NMR (151 MHz, DMSO-d_6) of **S1**

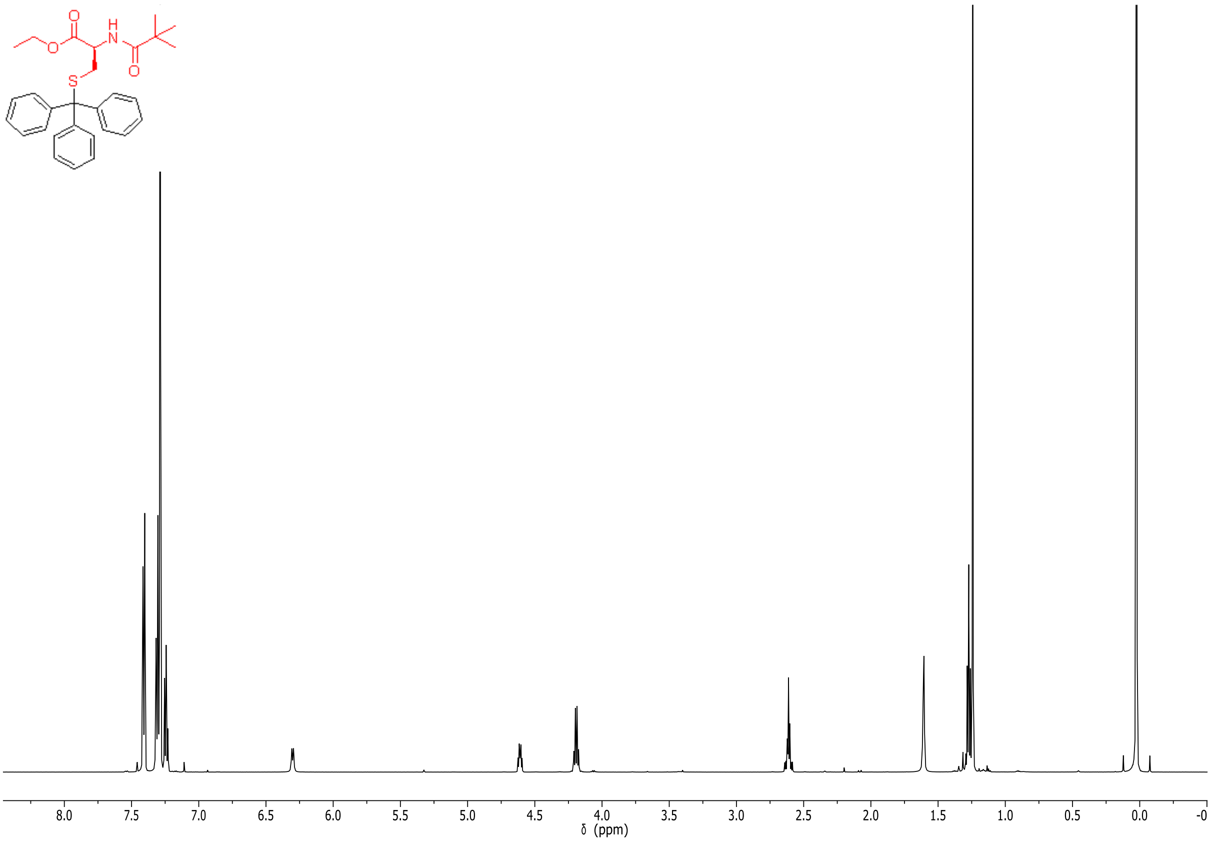

Spectrum 3. ^{1}H NMR (600 MHz, $CDCl_3$) of **S2**

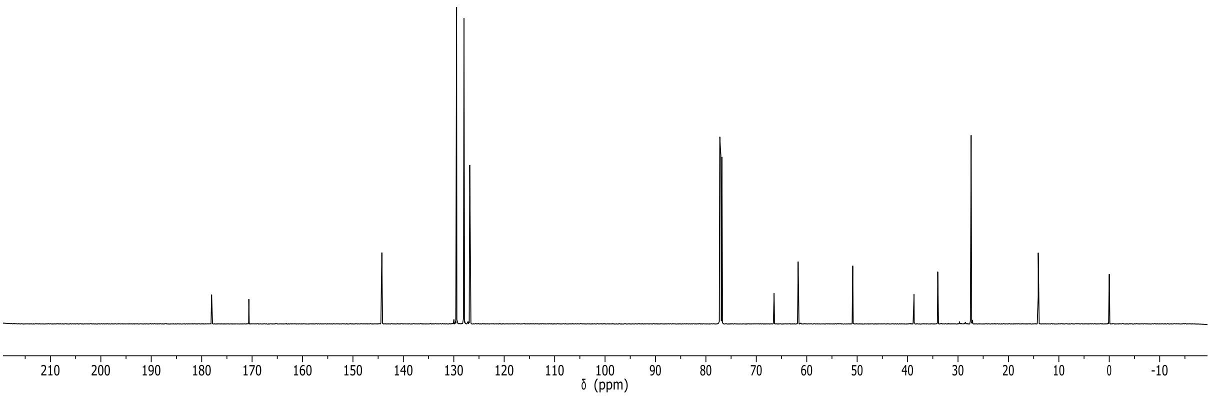

Spectrum 4. ^{13}C NMR (151 MHz, $CDCl_3$) of **S2**

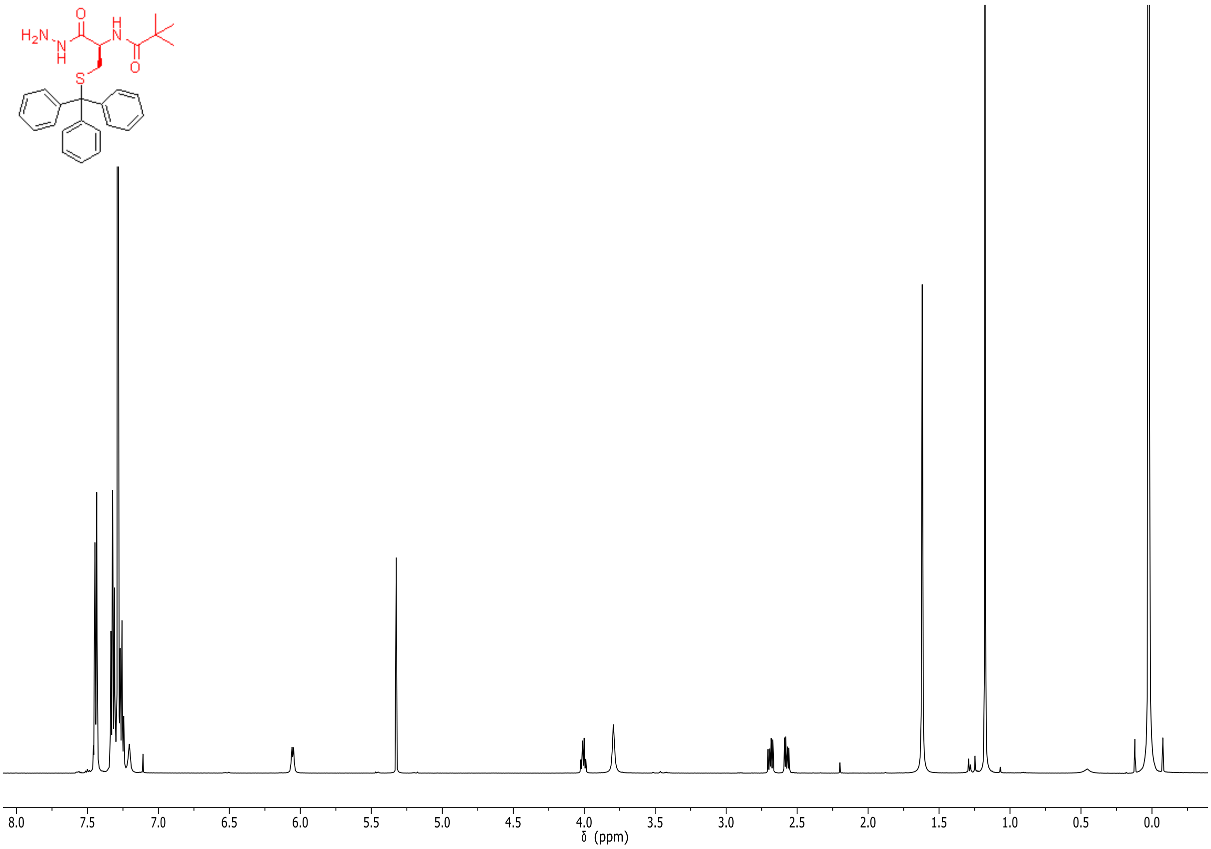

Spectrum 5. ^{1}H NMR (600 MHz,CDCl3) of **S3**

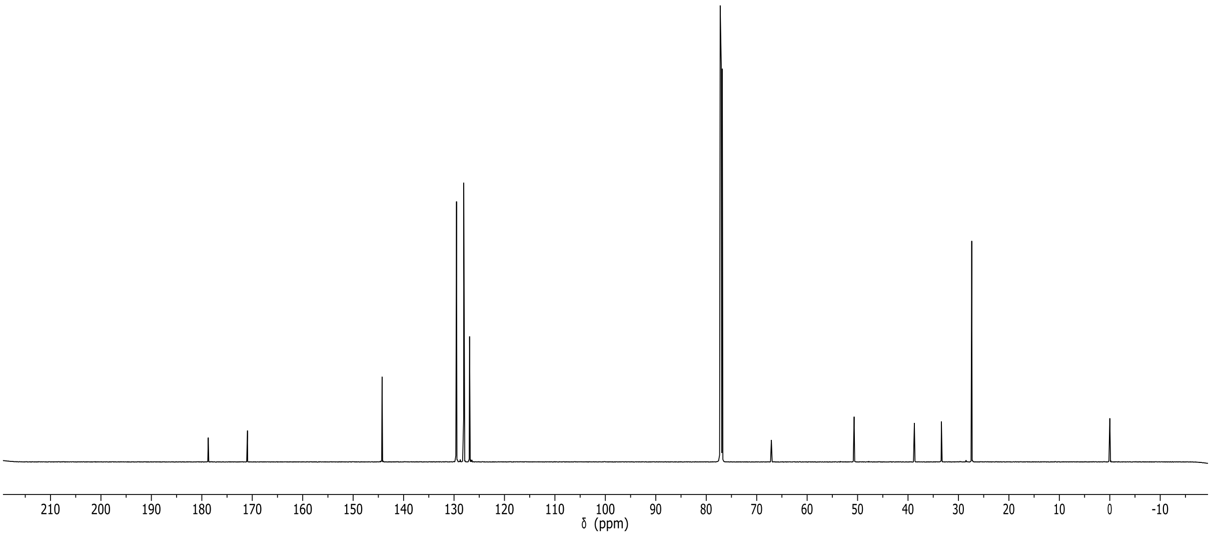

Spectrum 6. ^{13}C NMR (151 MHz, $CDCl_3$) of **S3**

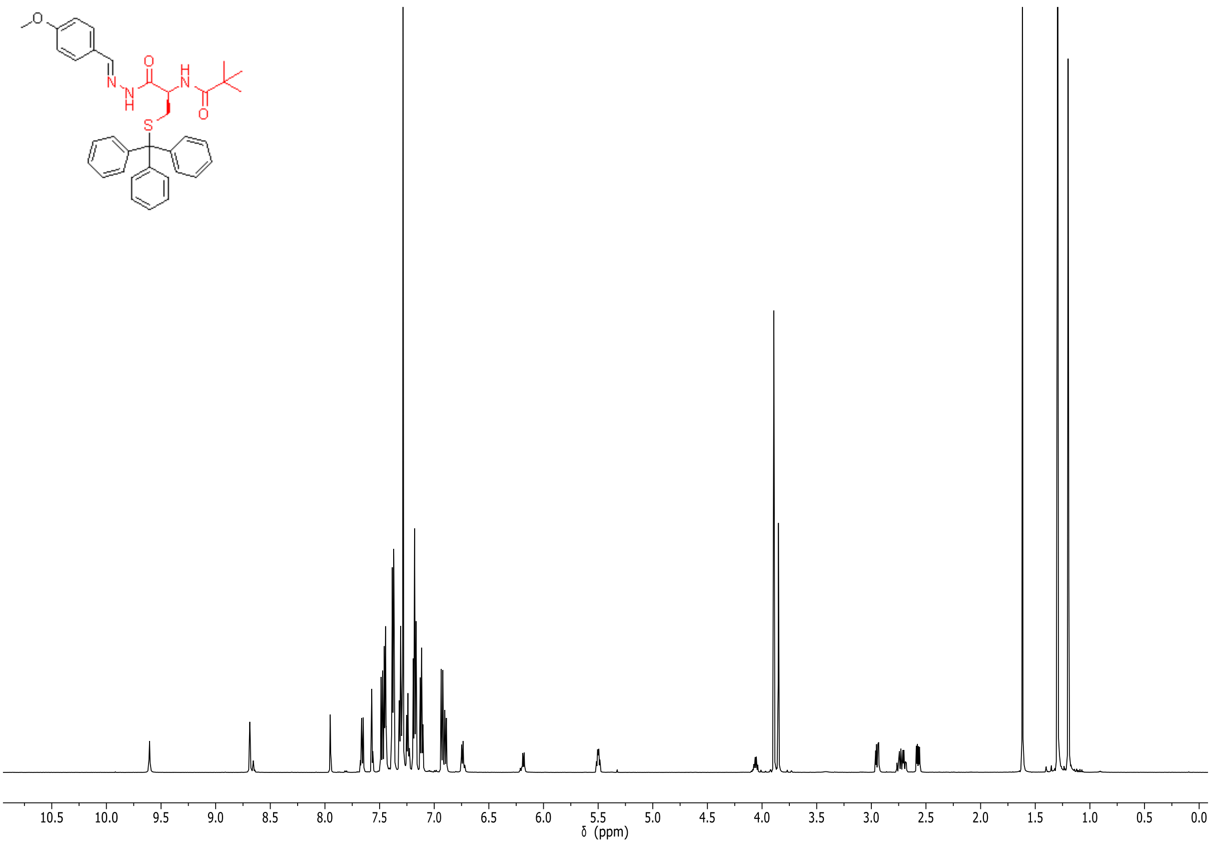

Spectrum 7. ^{1}H NMR (600 MHz, $CDCl_3$) of **3**

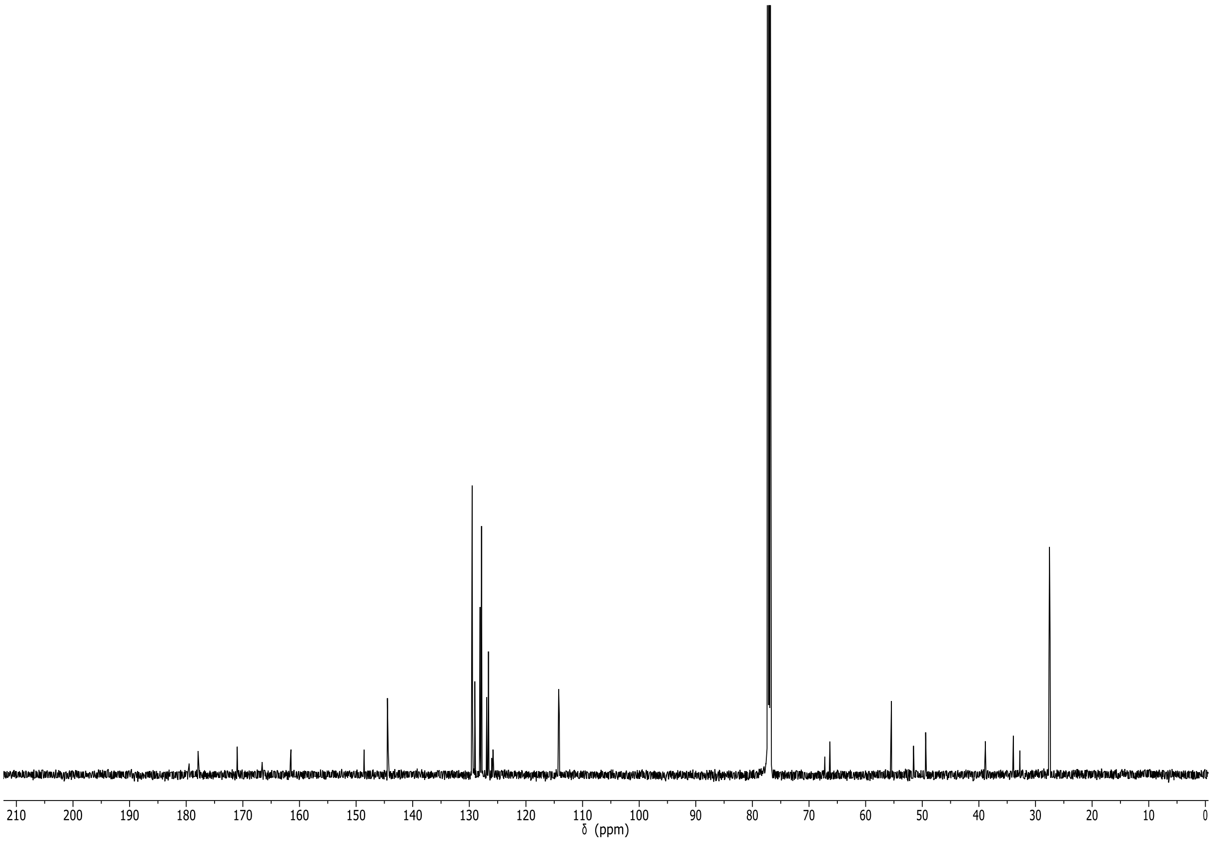

Spectrum 8. ^{13}C NMR (151 MHz, $CDCl_3$) of **3**

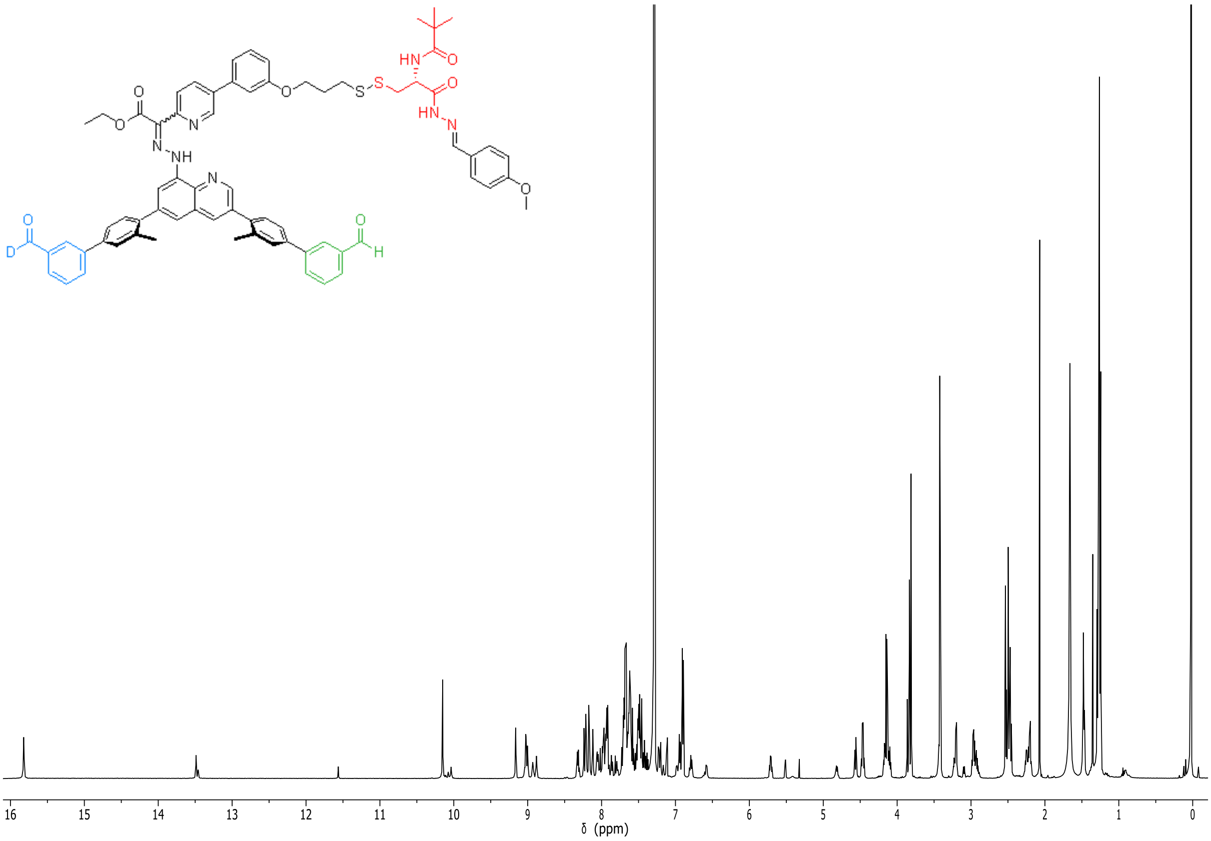

Spectrum 9. ^{1}H NMR (600 MHz, $CDCl_3$) of *EZ*-**6**

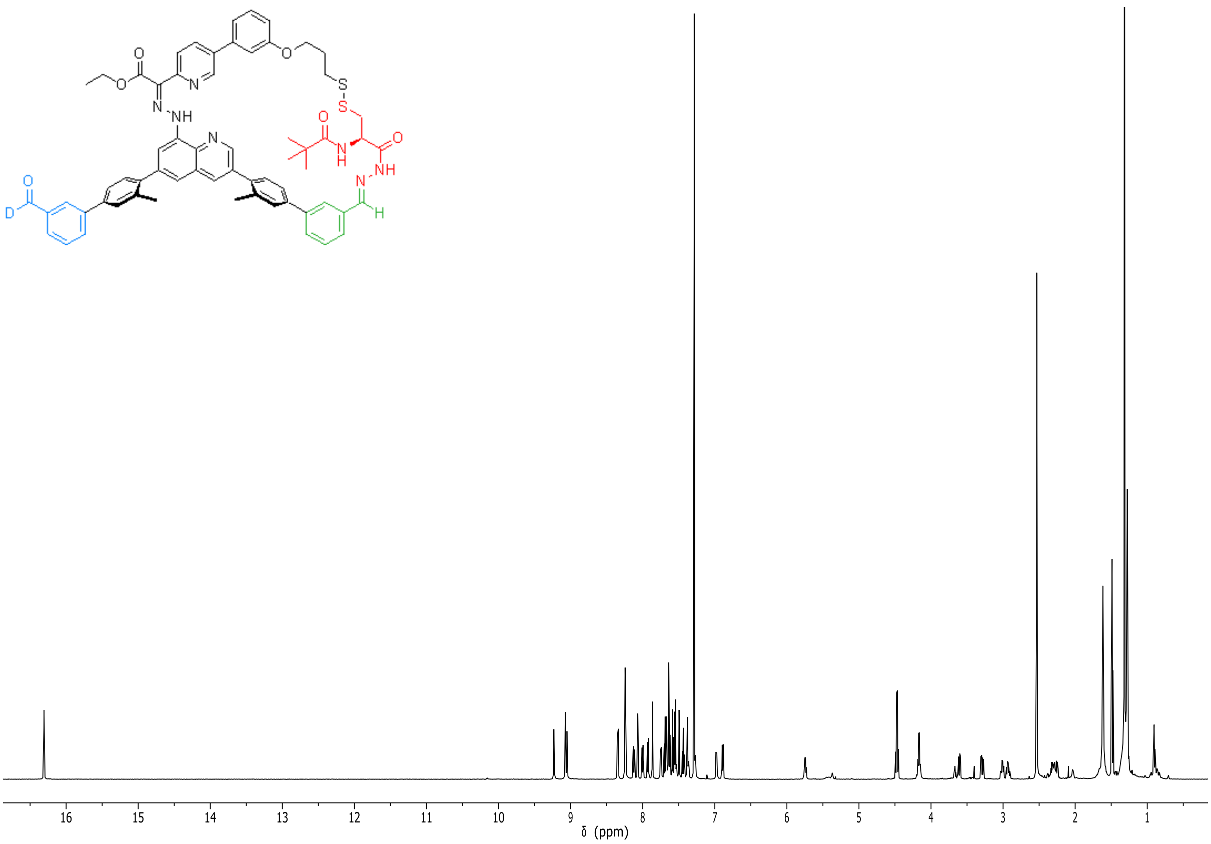

Spectrum 10. ^{1}H NMR (600 MHz, $CDCl_3$) of *E*-**9**

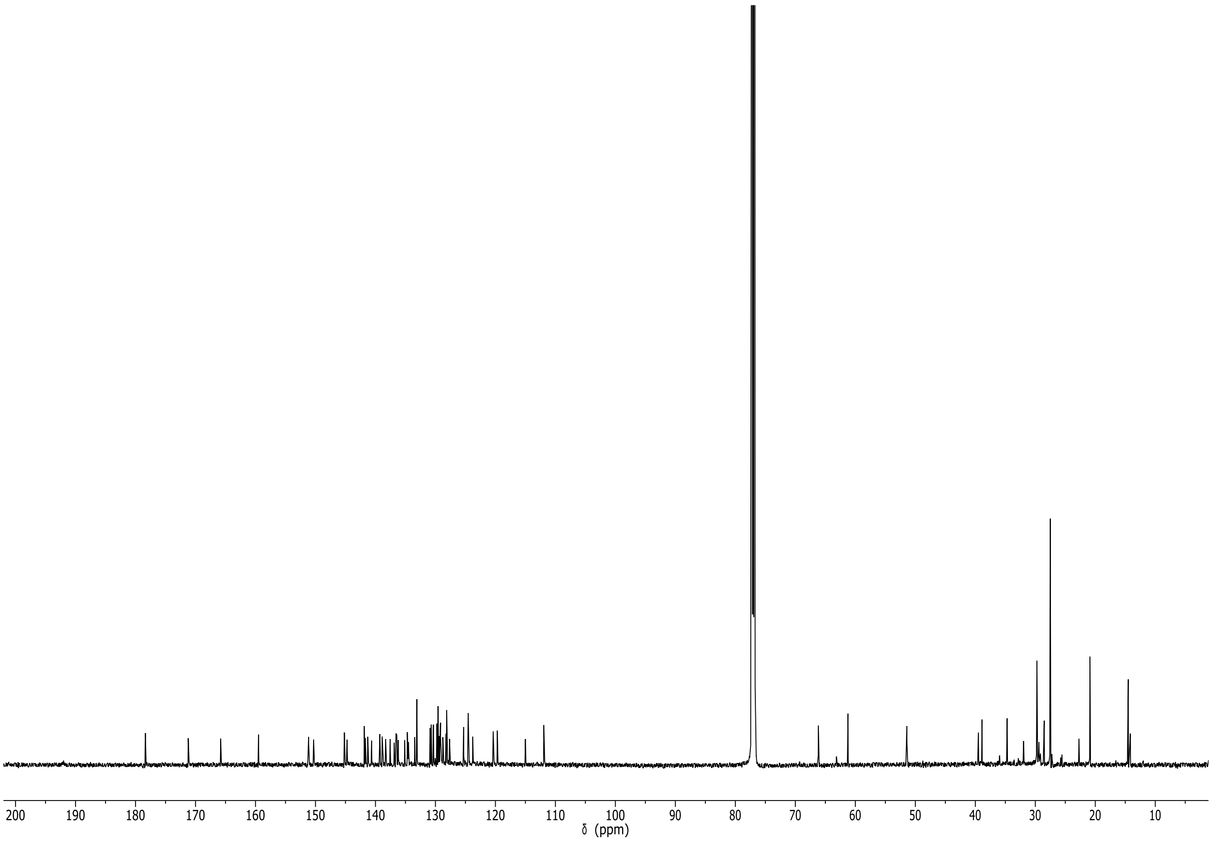

Spectrum 11. ^{13}C NMR (151 MHz, $CDCl_3$) of *E*-**9**

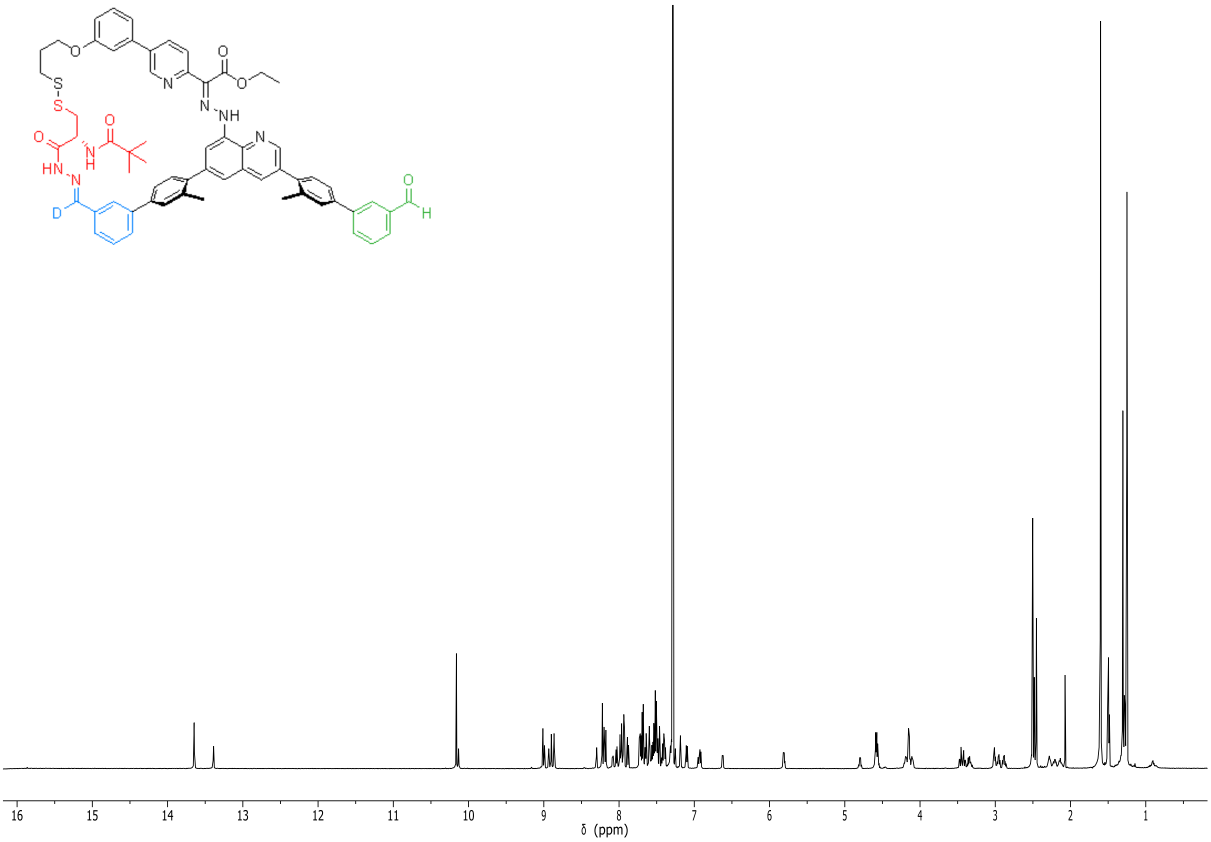

Spectrum 12. ^{1}H NMR (600 MHz, $CDCl_3$) of *Z*-**9**

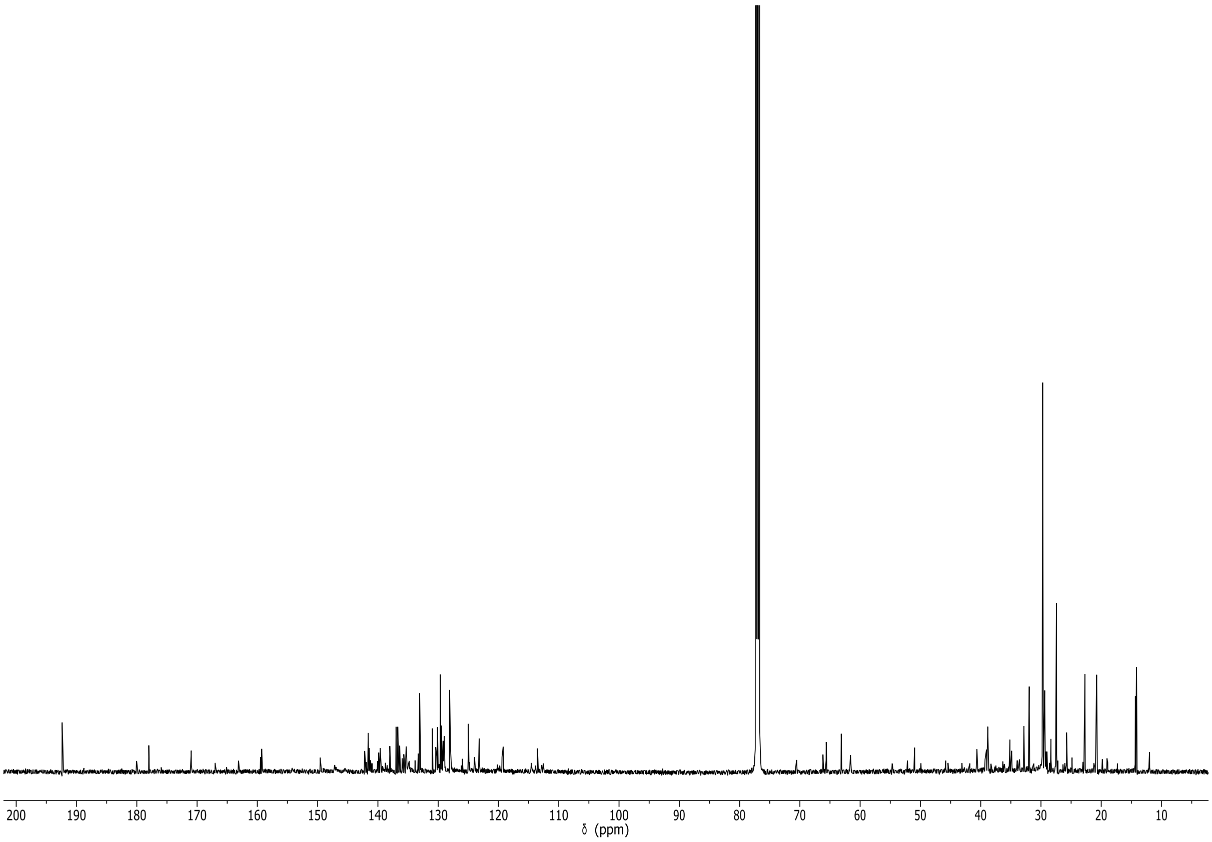

Spectrum 13. ^{13}C NMR (151 MHz, $CDCl_3$) of *Z*-**9**

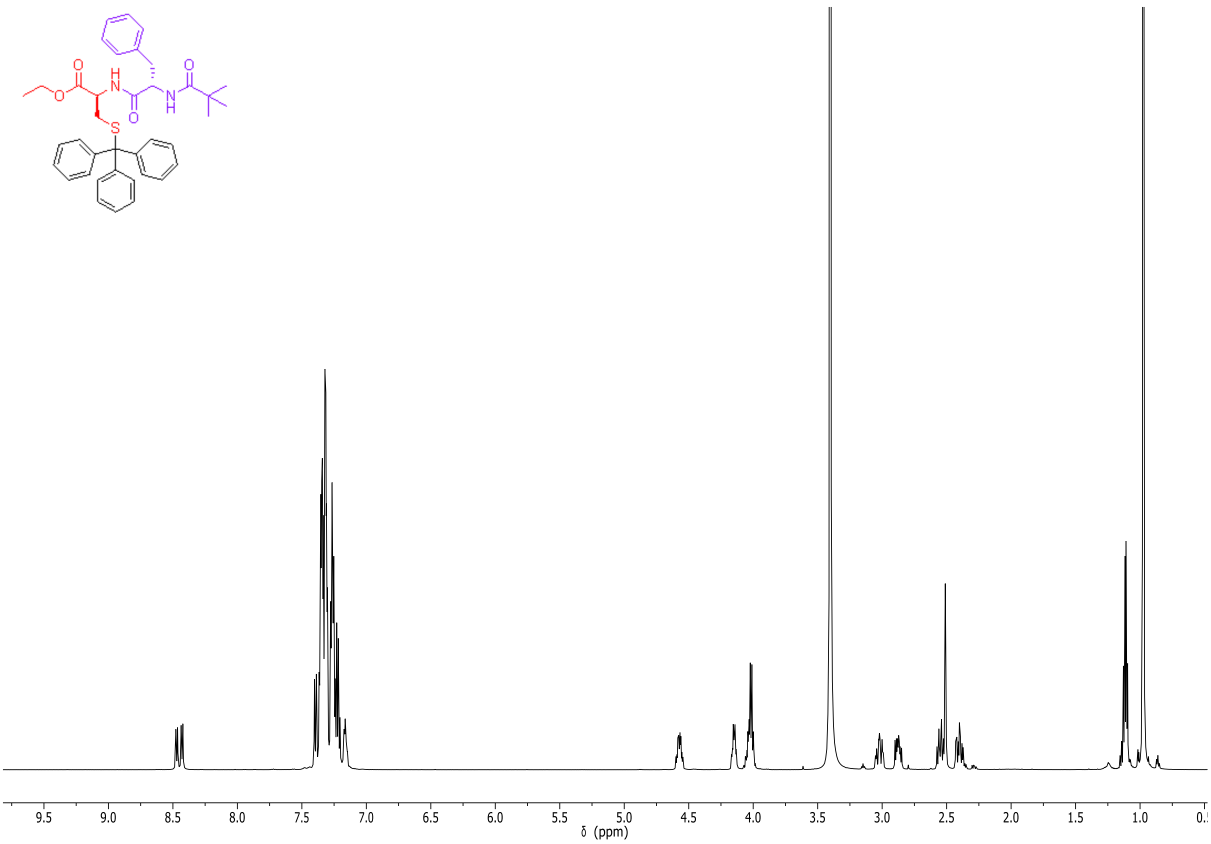

Spectrum 14. ^{1}H NMR (600 MHz, DMSO-d_6) of **S4**

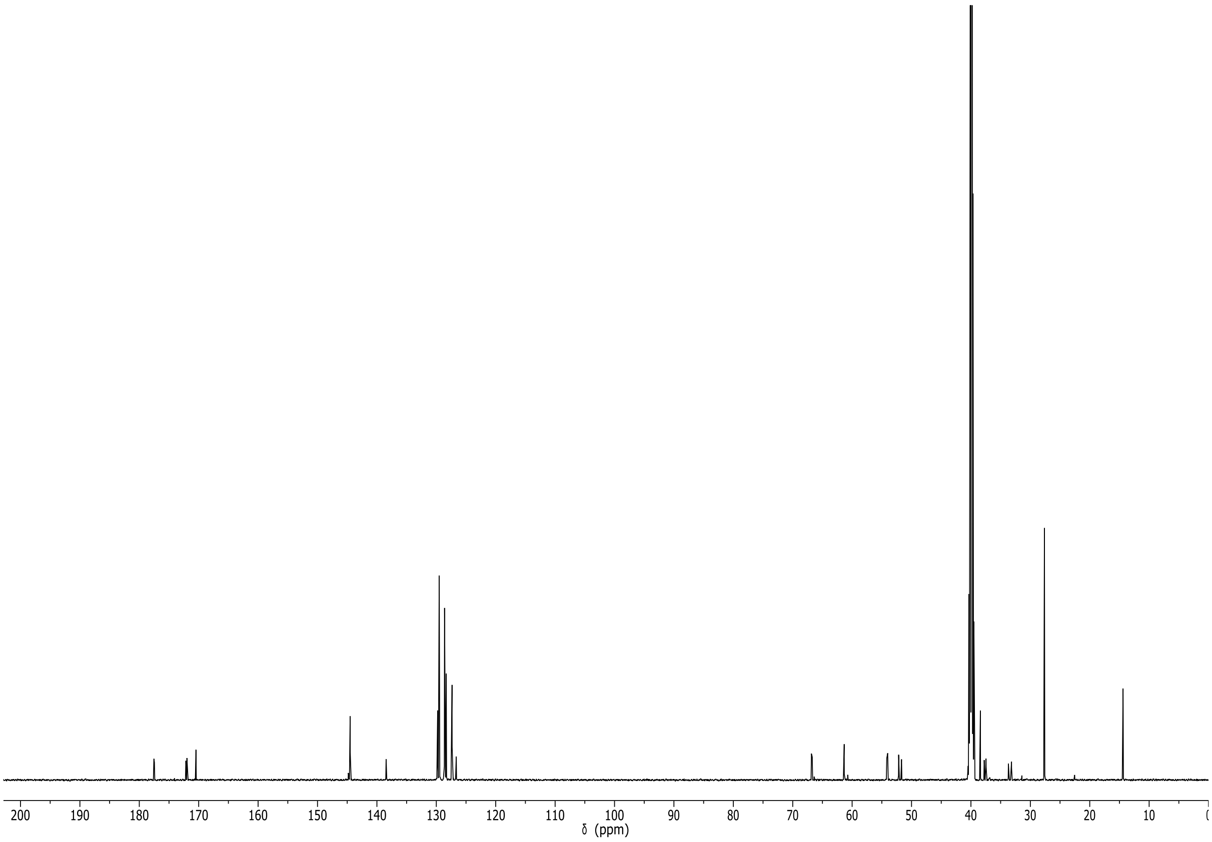

Spectrum 15. ^{13}C NMR (151 MHz, $CDCl_3$) of **S4**

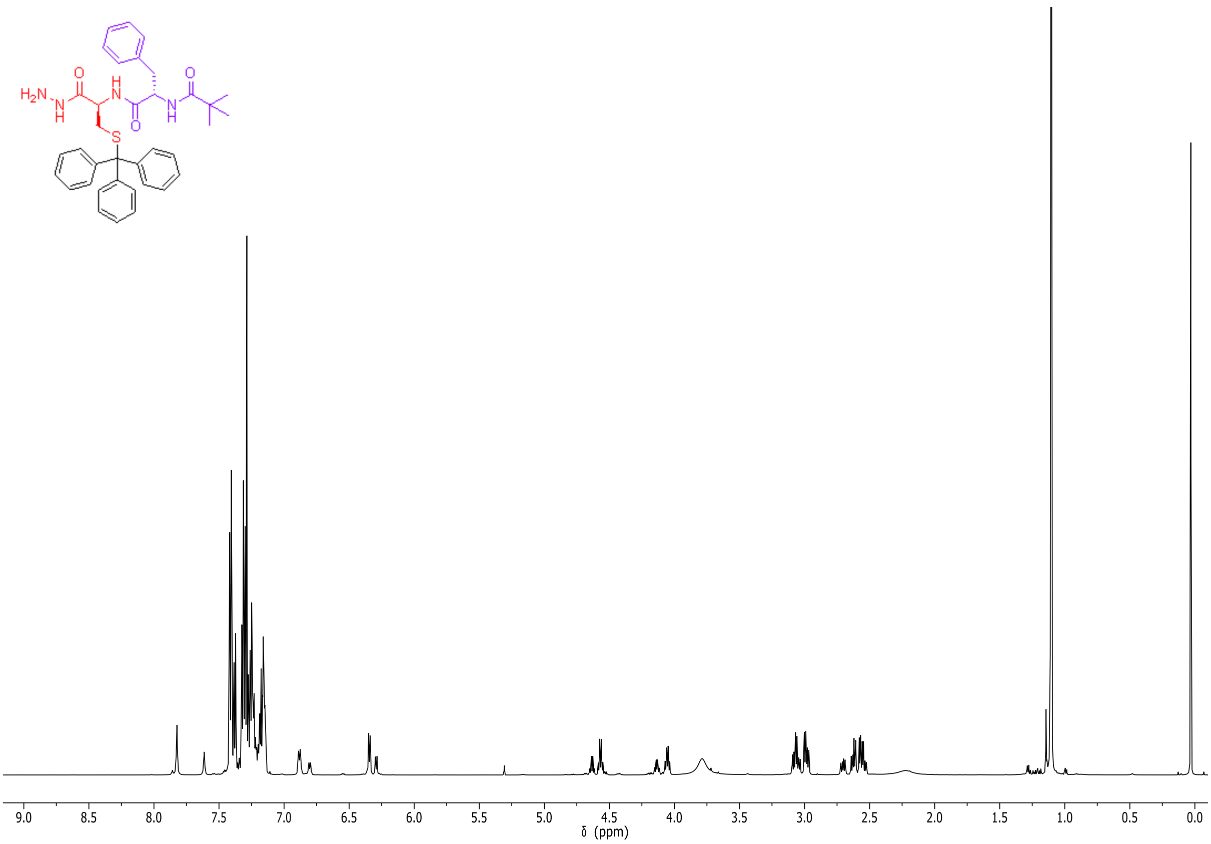

Spectrum 16. ^{1}H NMR (600 MHz, $CDCl_3$) of **S5**

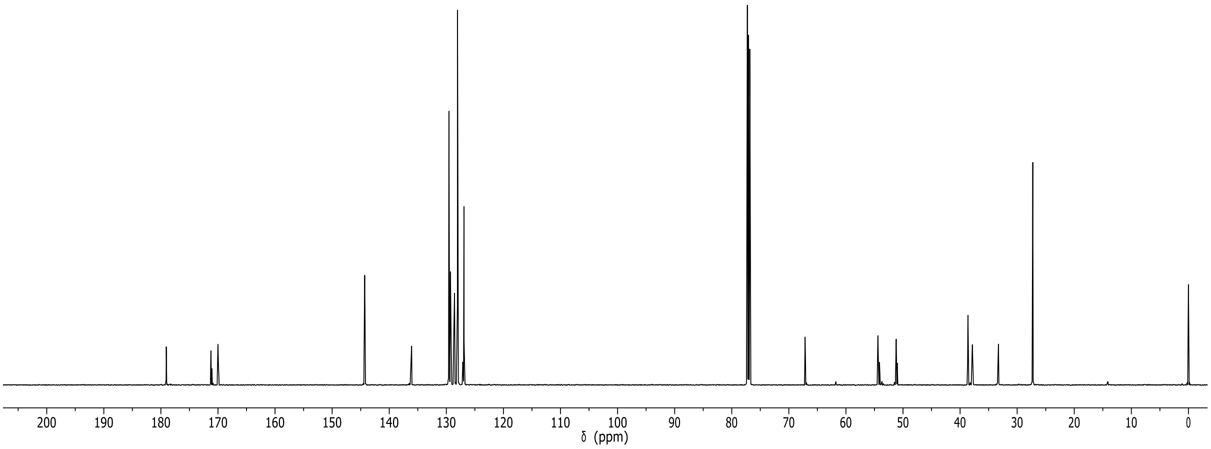

Spectrum 17. ^{13}C NMR (151 MHz, $CDCl_3$) of **S5**

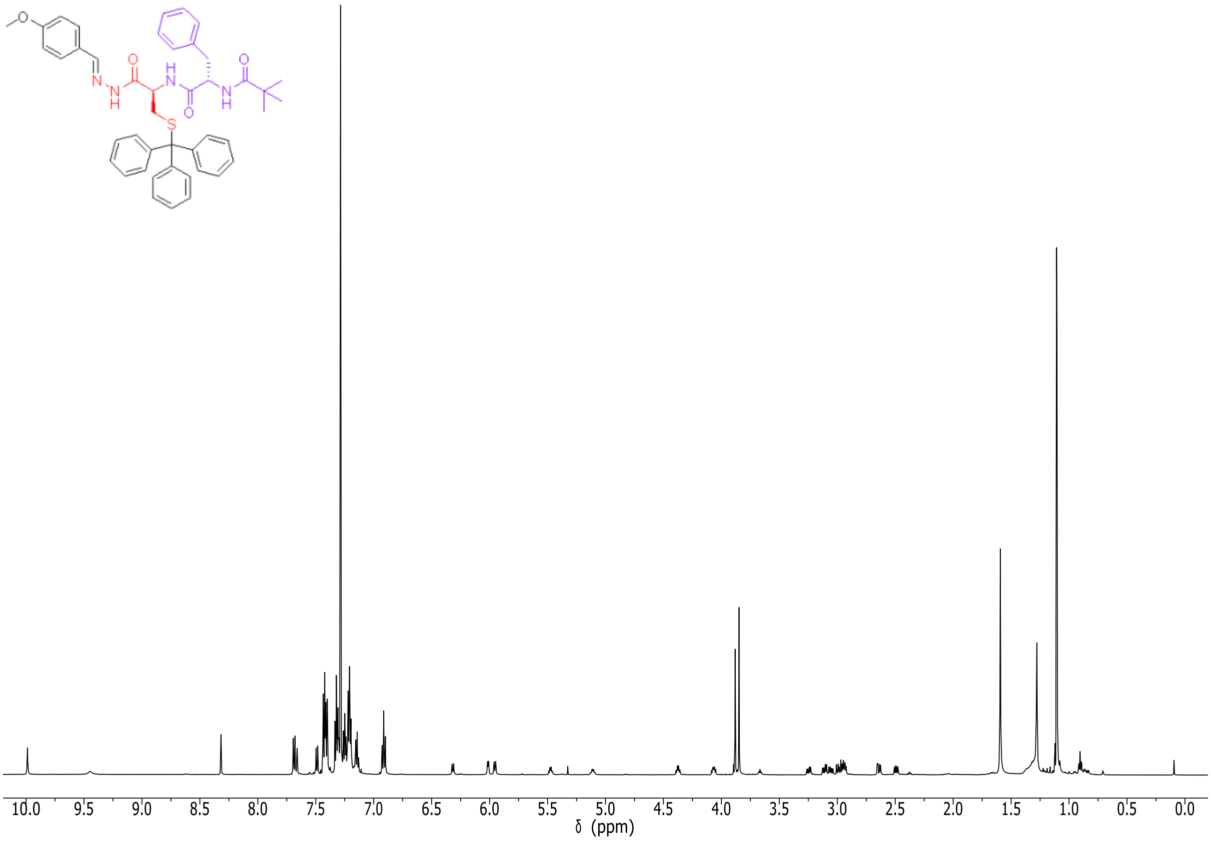

Spectrum 18. ^{1}H NMR (600 MHz, $CDCl_3$) of **4**

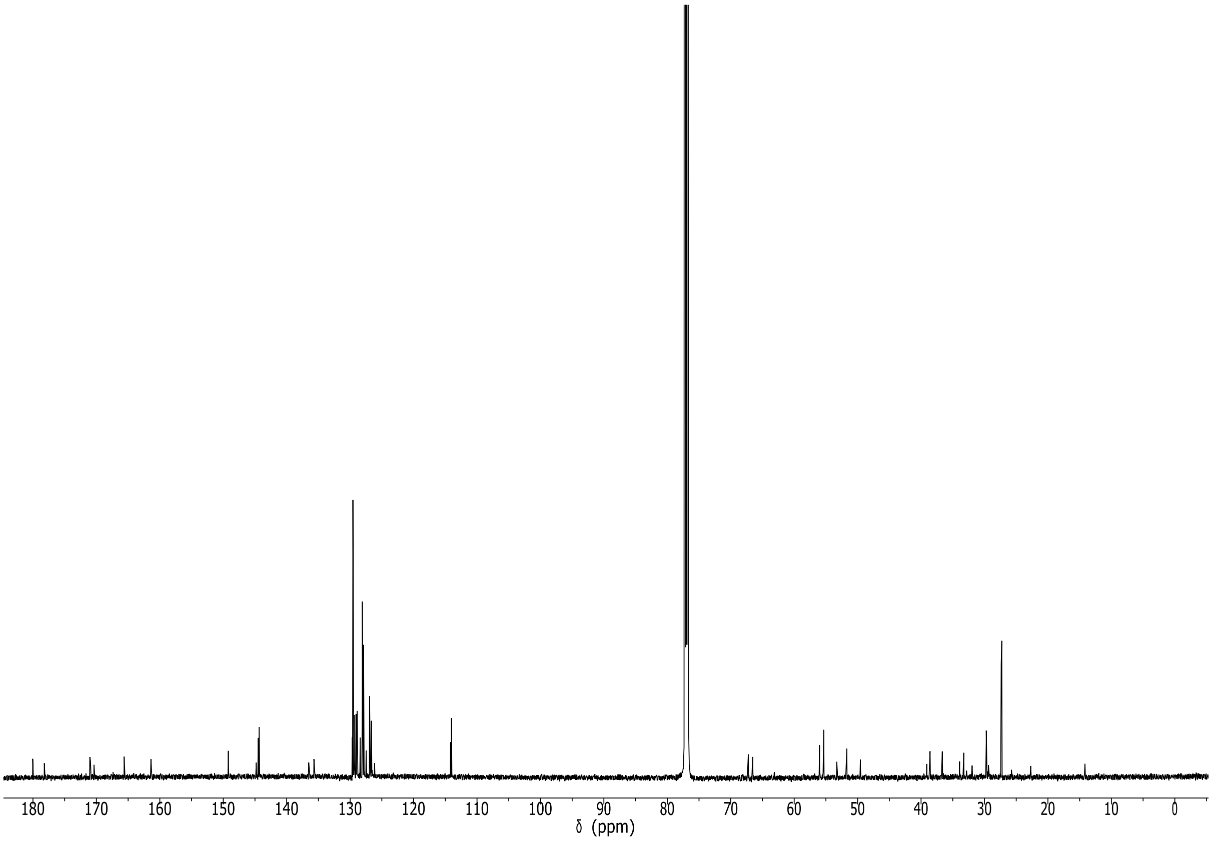

Spectrum 19. ^{13}C NMR (151 MHz, $CDCl_3$) of **4**

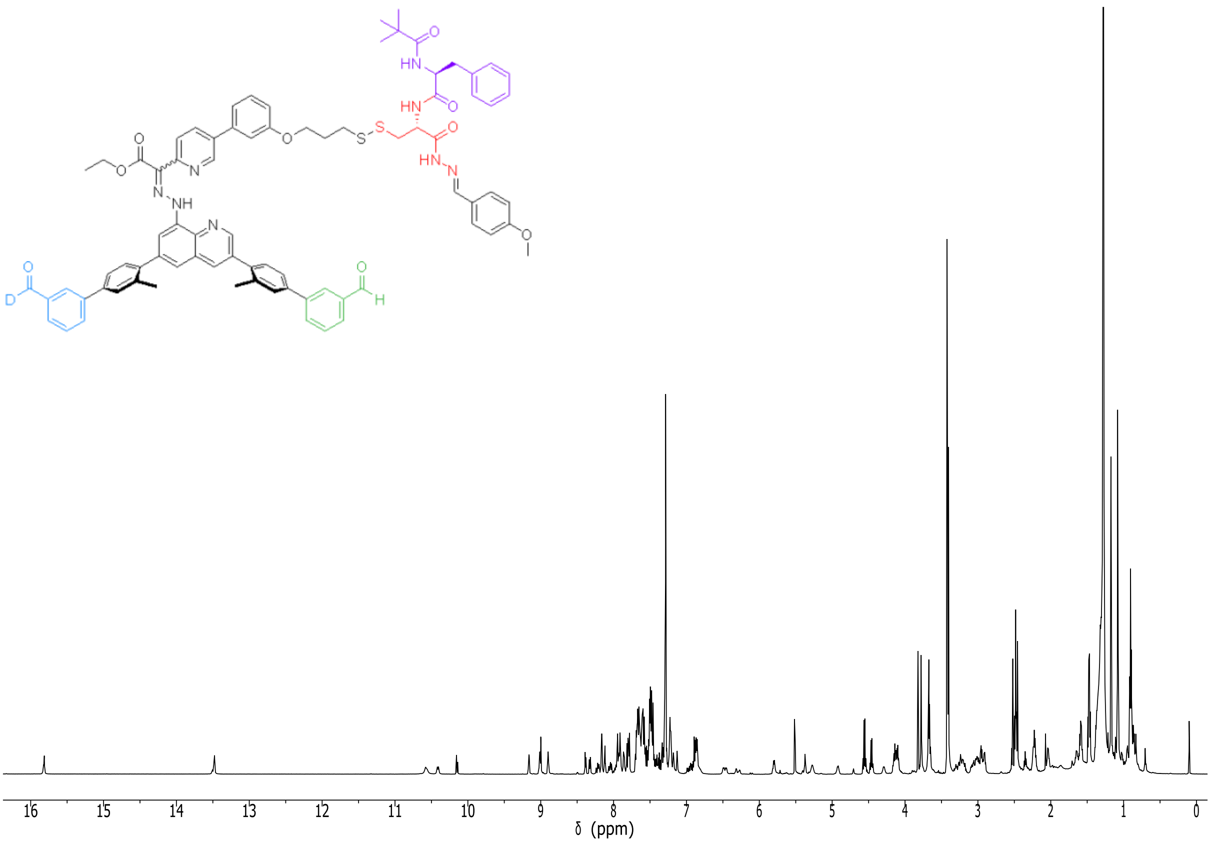

Spectrum 20. ^{1}H NMR (600 MHz, $CDCl_3$) of *EZ*-**7**

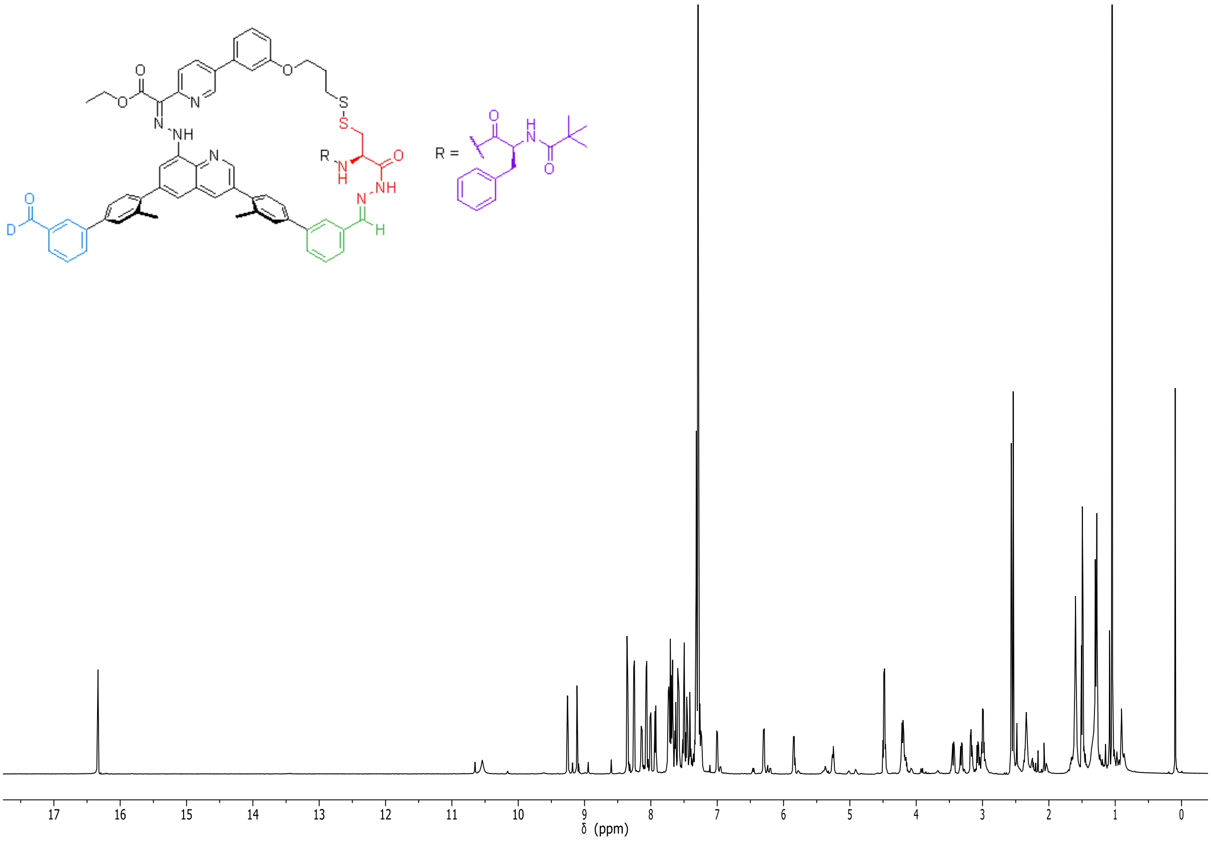

Spectrum 21. ^{1}H NMR (600 MHz, $CDCl_3$) of *E*-**10**

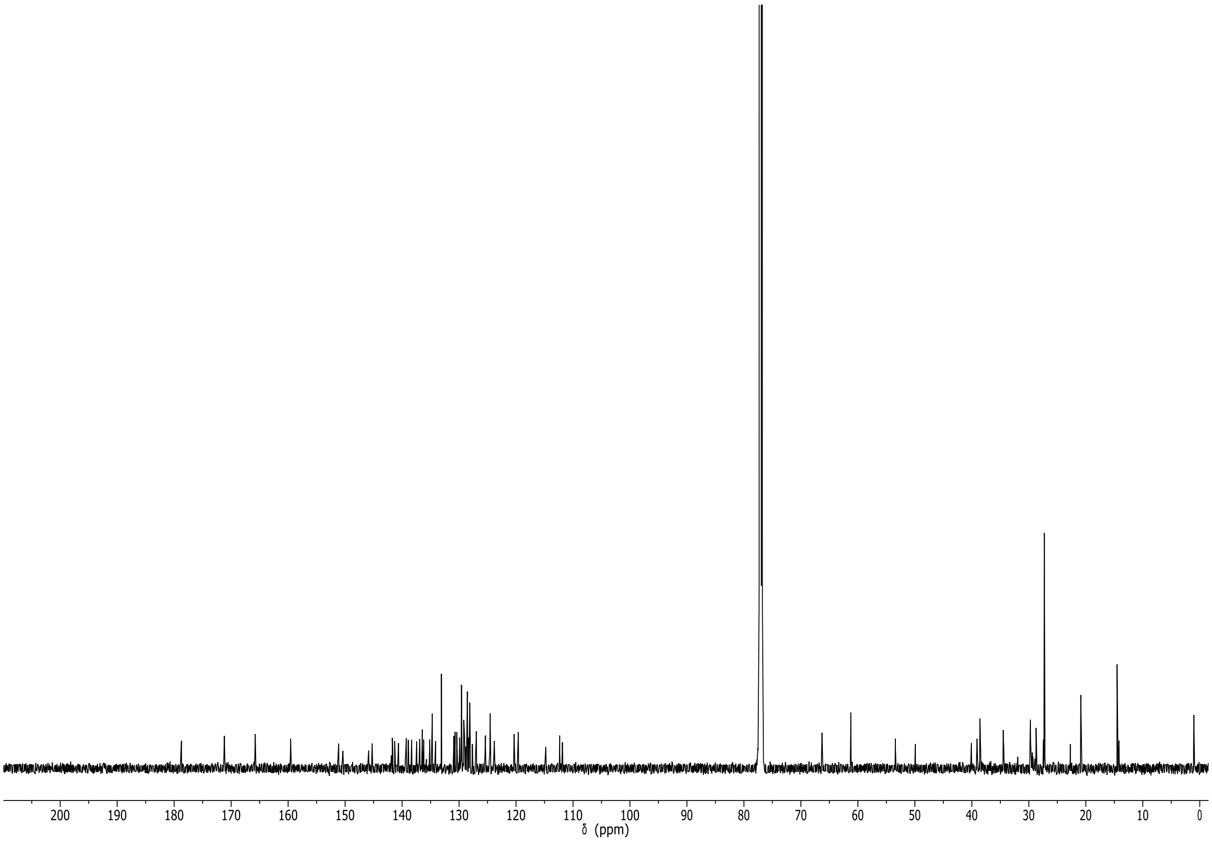

Spectrum 22. ^{13}C NMR (151 MHz, $CDCl_3$) of *E*-**10**

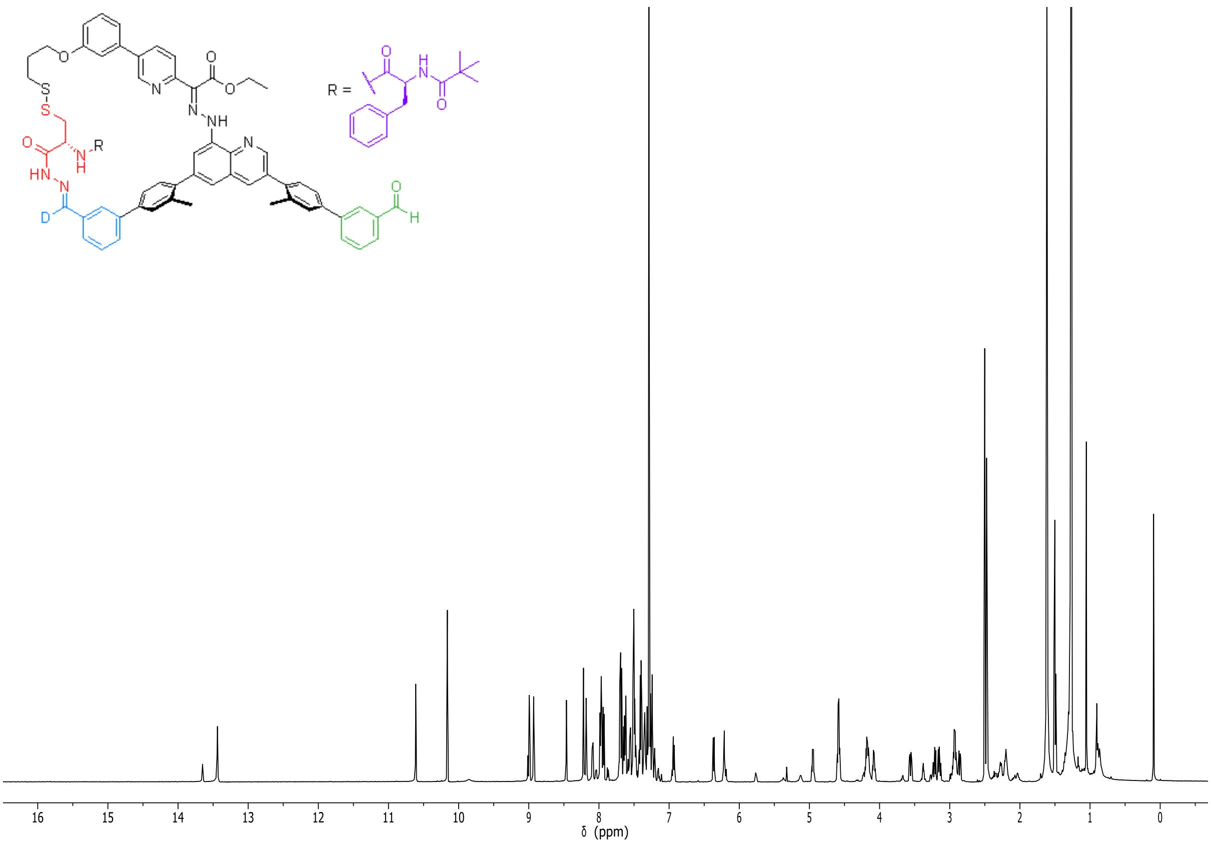

Spectrum 23. ^{1}H NMR (600 MHz, $CDCl_3$) of *Z*-**10**

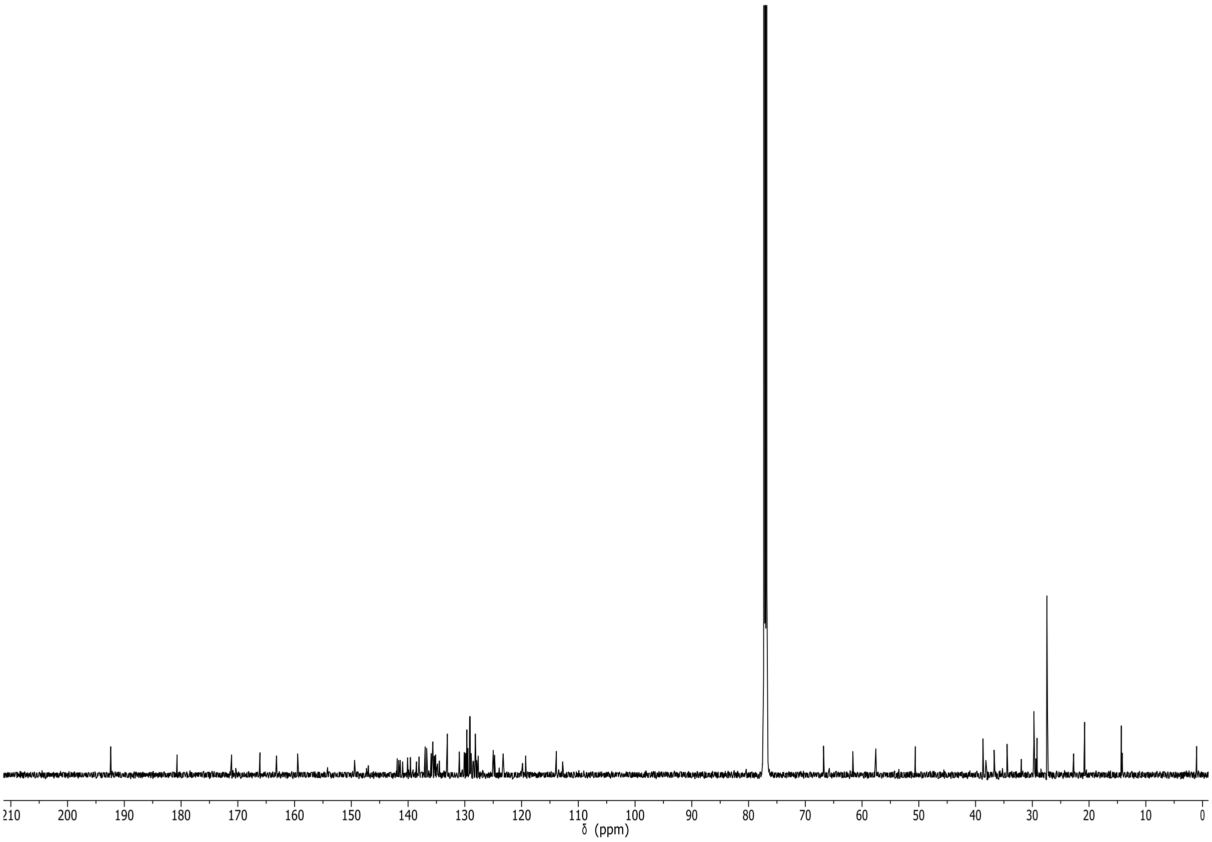

Spectrum 24. ^{13}C NMR (151 MHz, $CDCl_3$) of *Z*-**10**

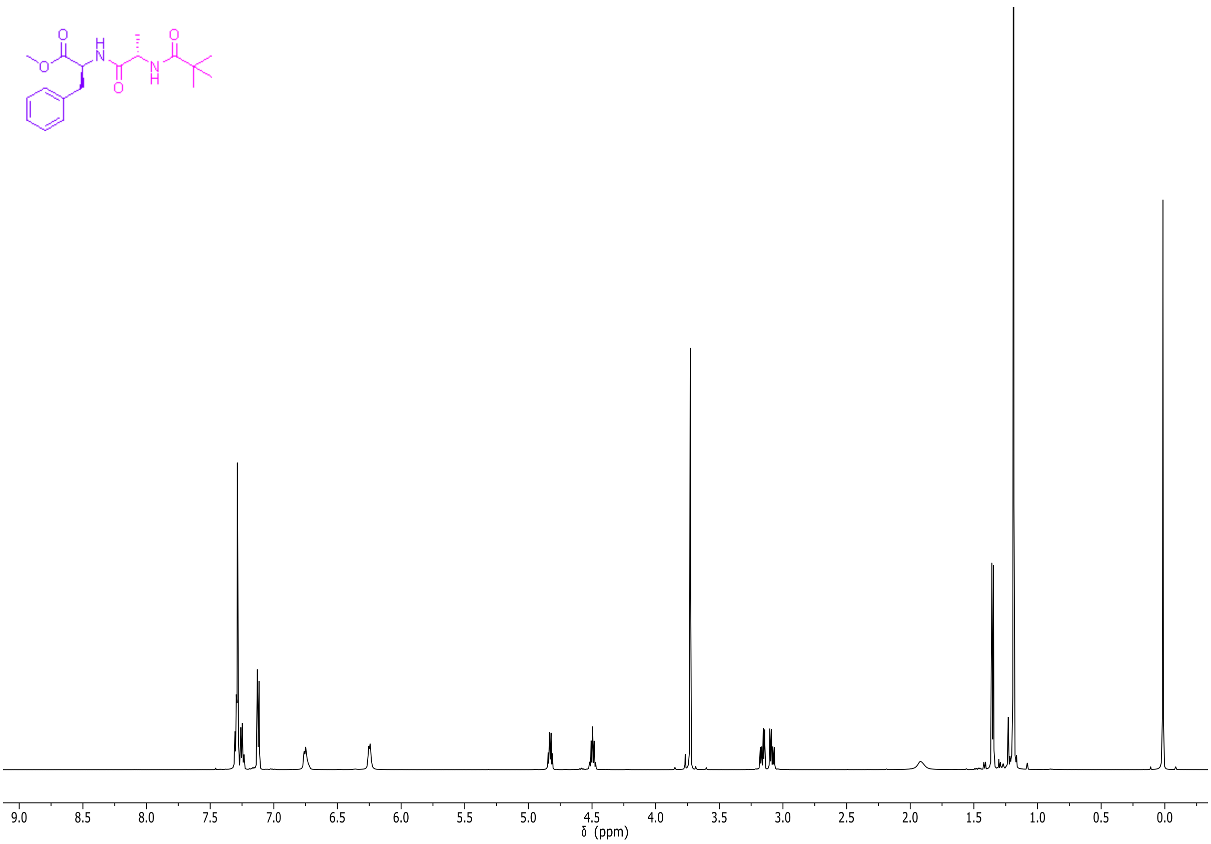

Spectrum 25. ^{1}H NMR (600 MHz, $CDCl_3$) of **S6**

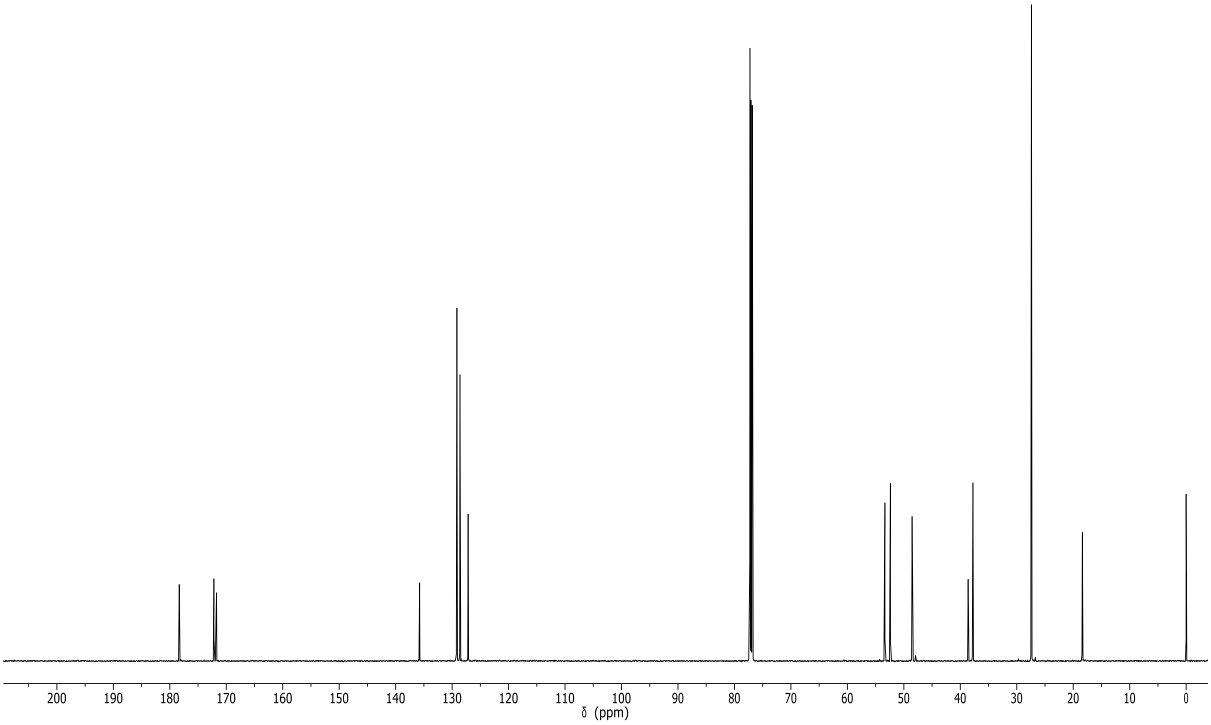

Spectrum 26. ^{13}C NMR (151 MHz, $CDCl_3$) of **S6**

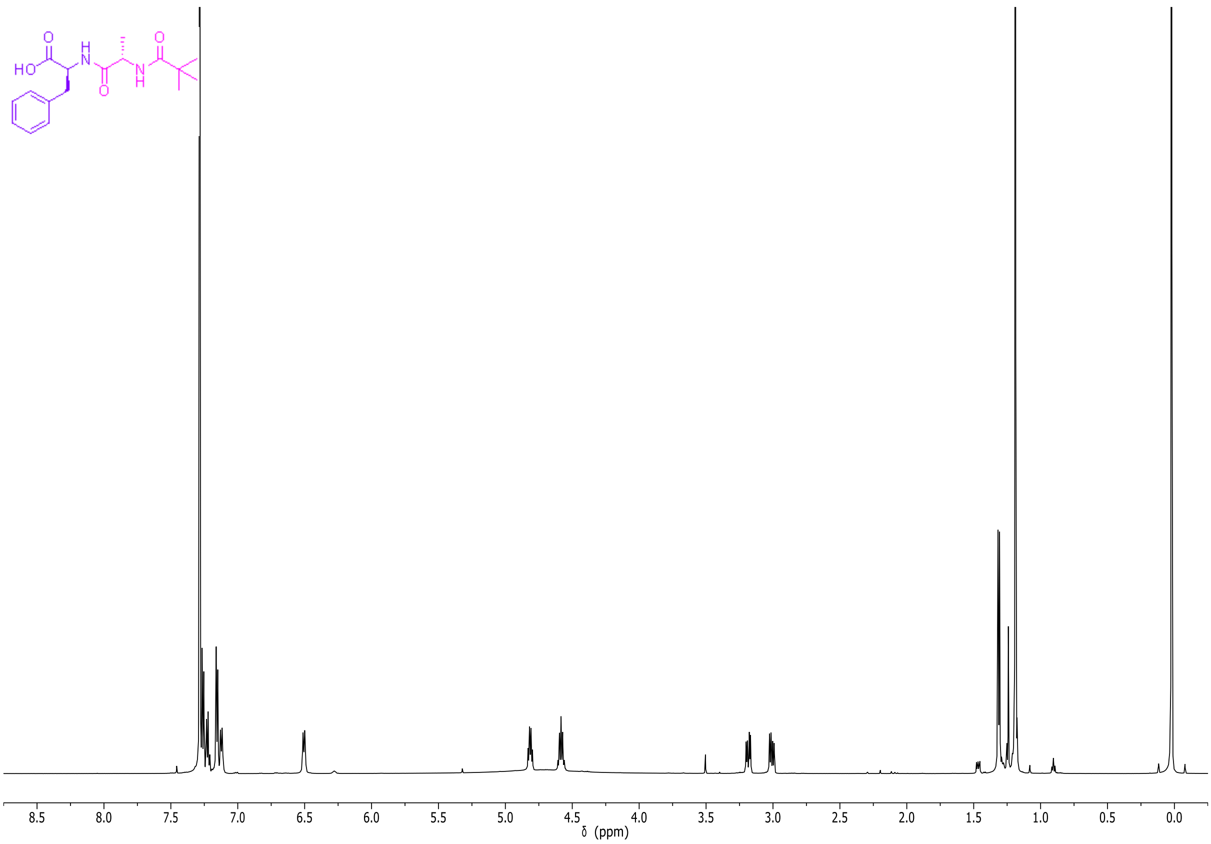

Spectrum 27. ^{1}H NMR (600 MHz, $CDCl_3$) of **S7**

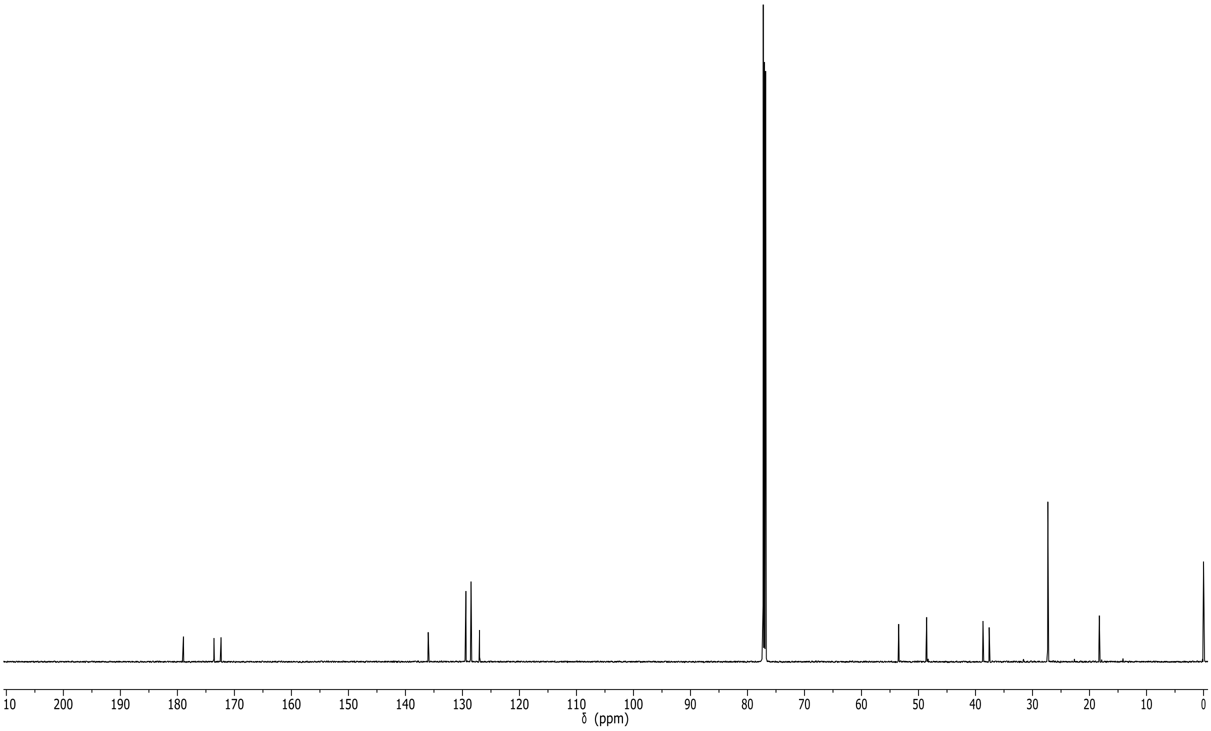

Spectrum 28. ^{13}C NMR (151 MHz, $CDCl_3$) of **S7**

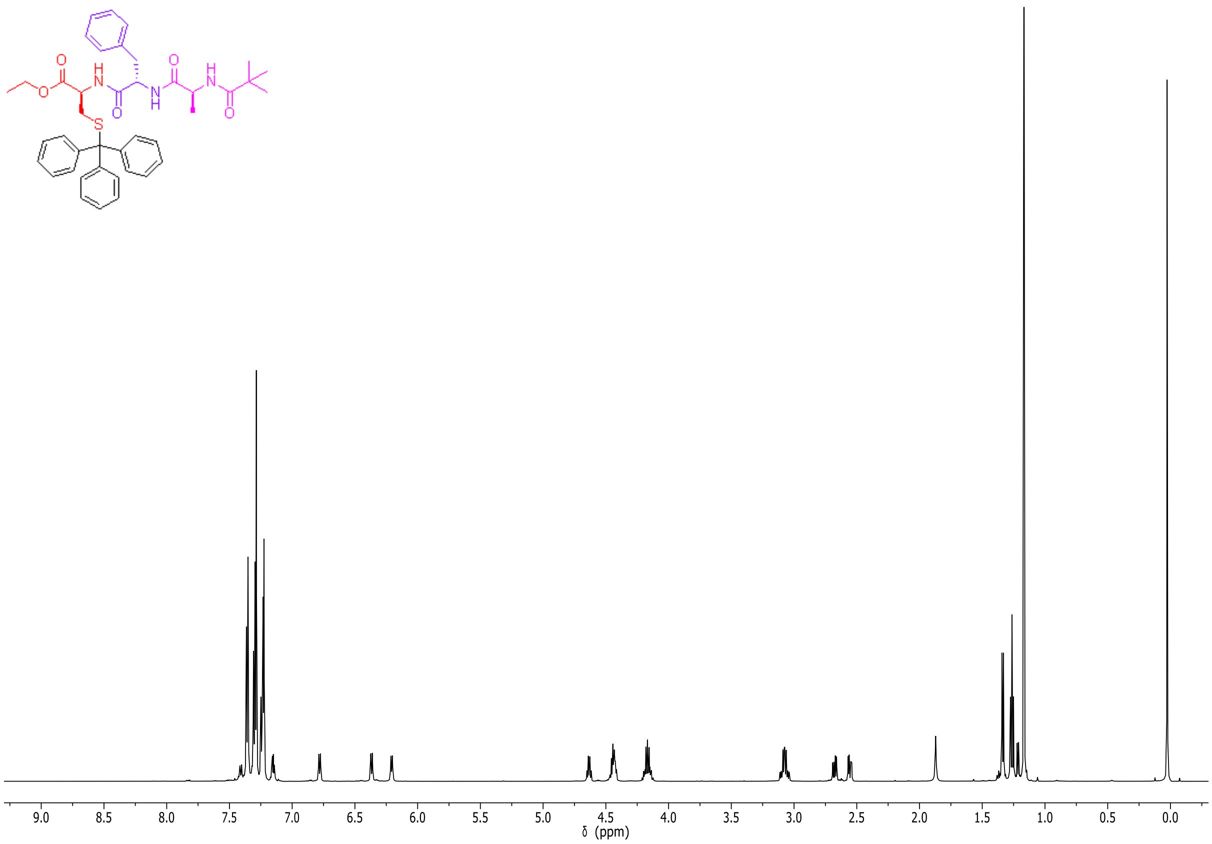

Spectrum 29. ^{1}H NMR (600 MHz, $CDCl_3$) of **S8**

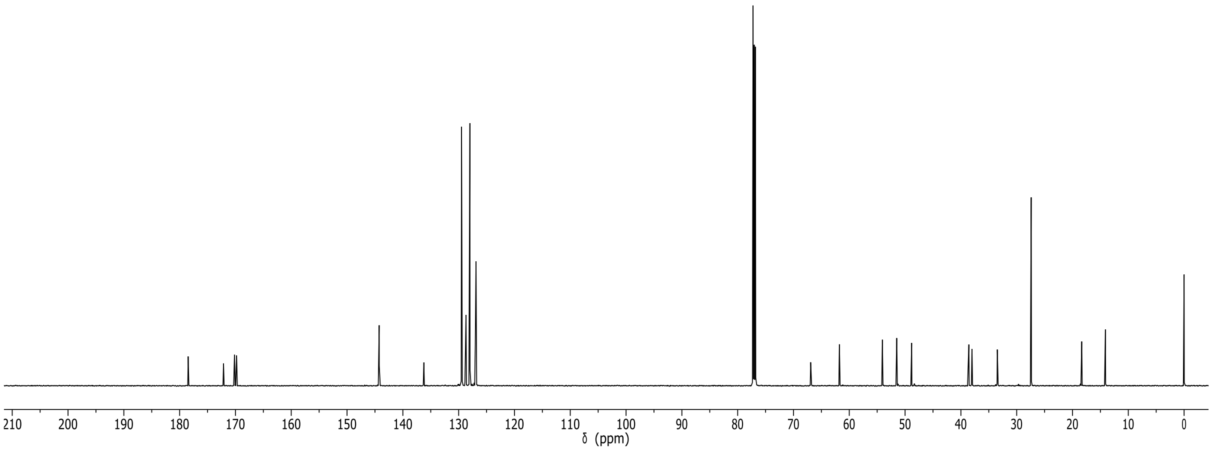

Spectrum 30. ^{13}C NMR (151 MHz, $CDCl_3$) of **S8**

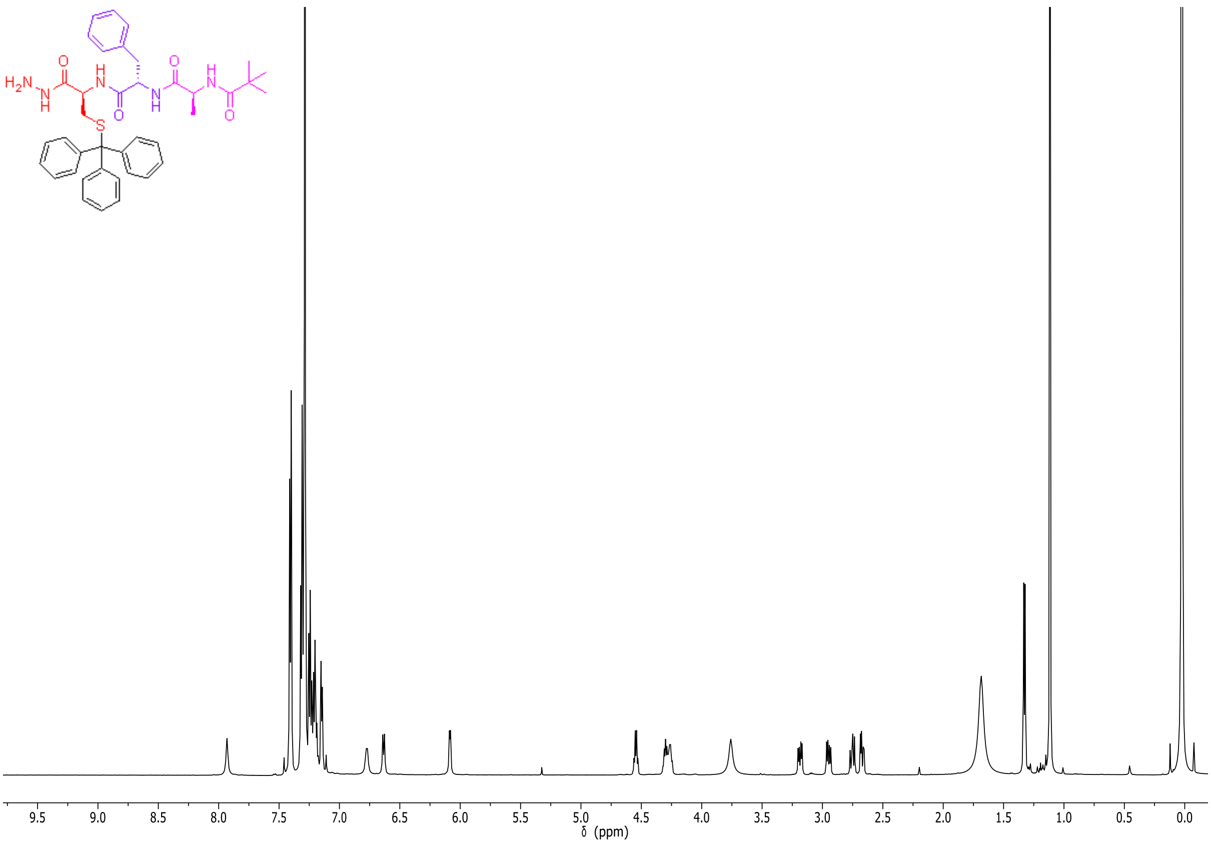

Spectrum 31. ^{1}H NMR (600 MHz, $CDCl_3$) of **S9**

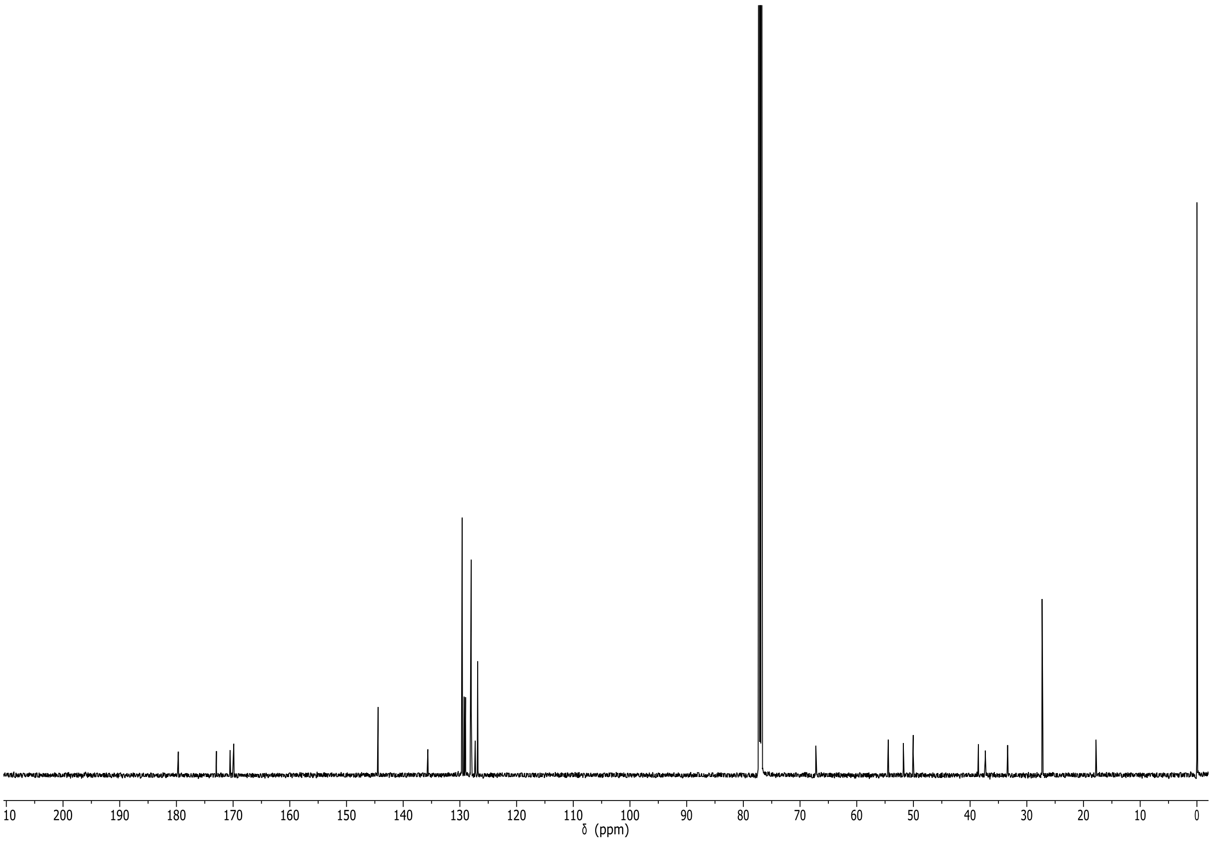

Spectrum 32. ^{13}C NMR (151 MHz, $CDCl_3$) of **S9**

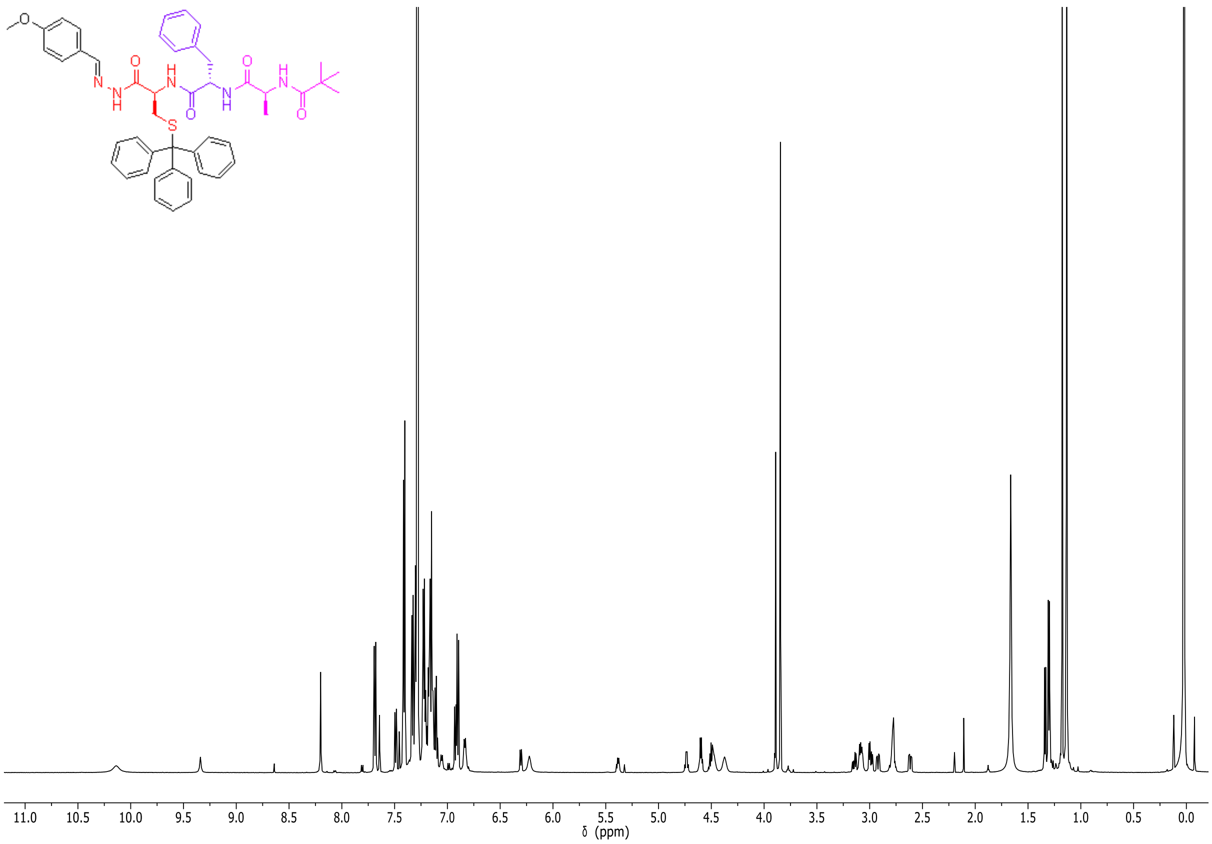

Spectrum 33. ^{1}H NMR (600 MHz, $CDCl_3$) of **5**

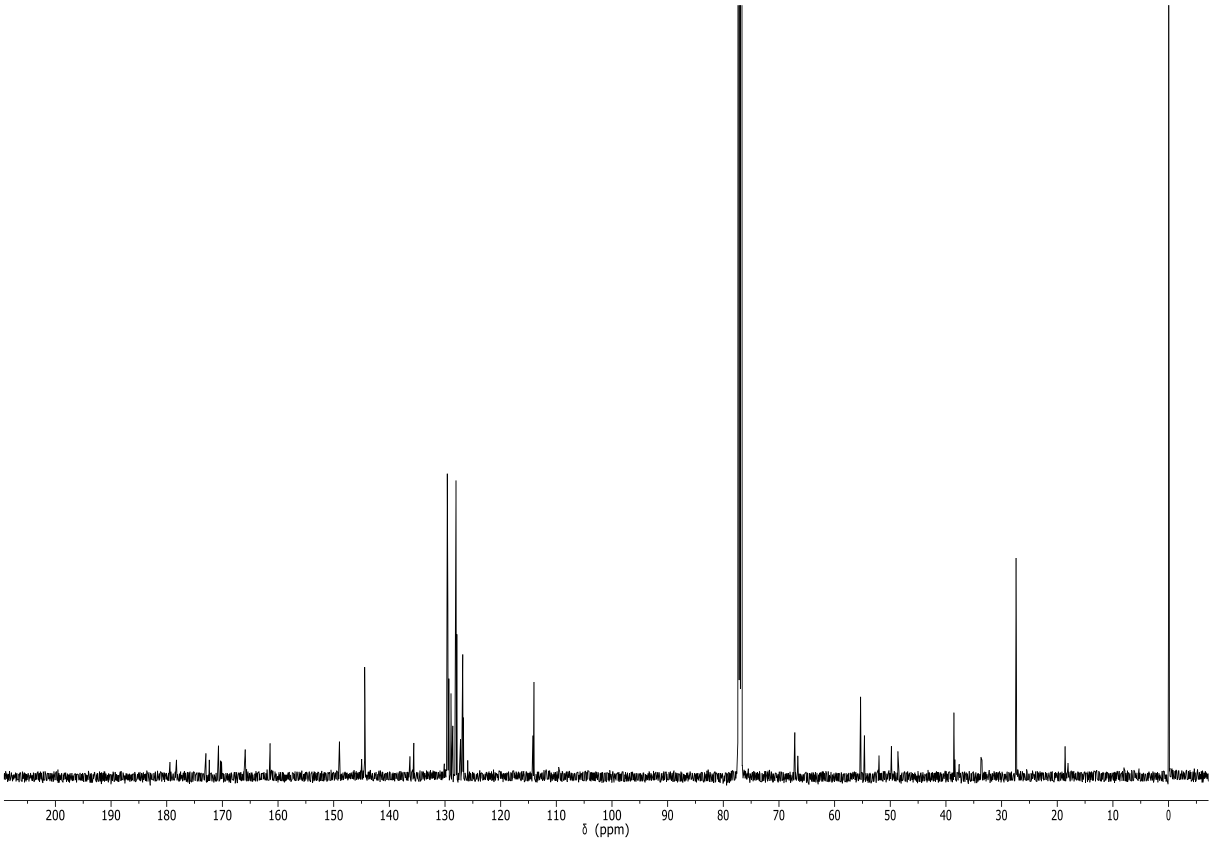

Spectrum 34. ^{13}C NMR (151 MHz, $CDCl_3$) of **5**

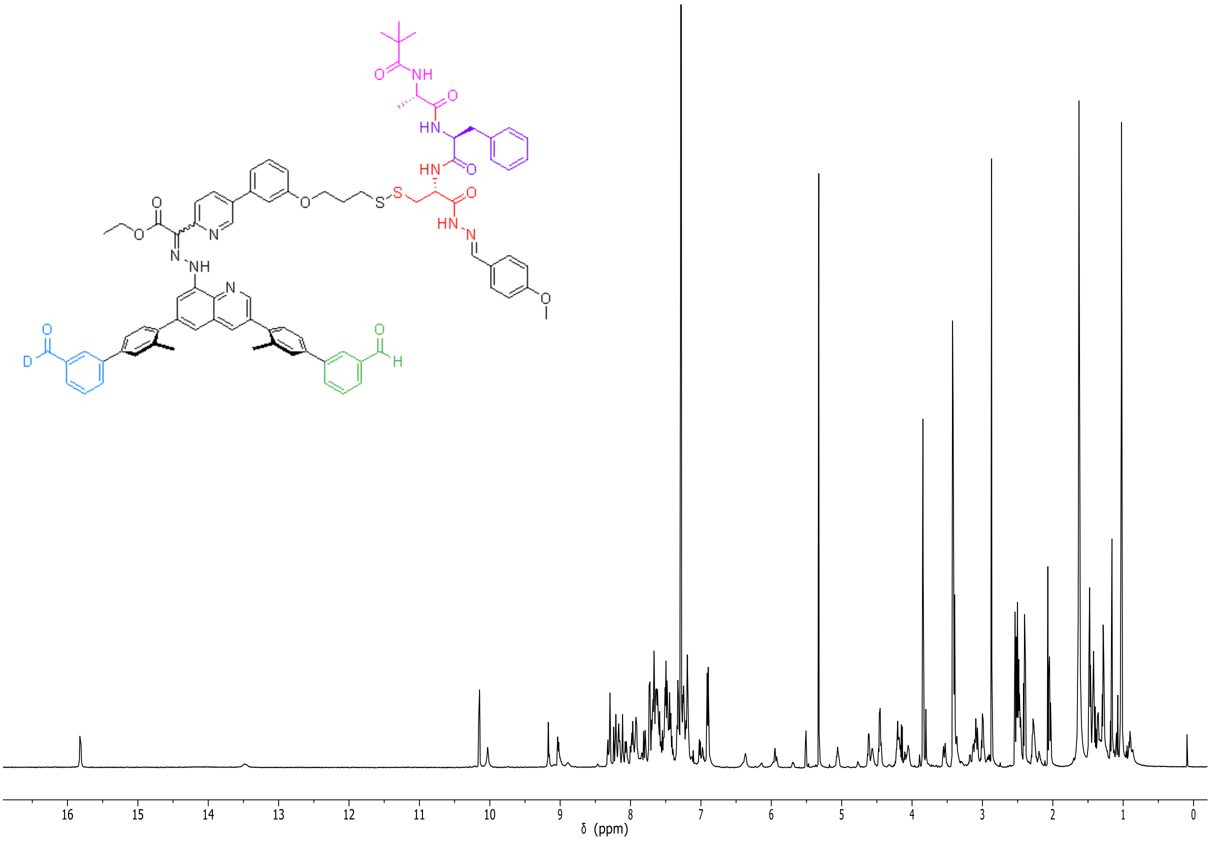

Spectrum 35. ^{1}H NMR (600 MHz, $CDCl_3$) of *EZ*-**8**

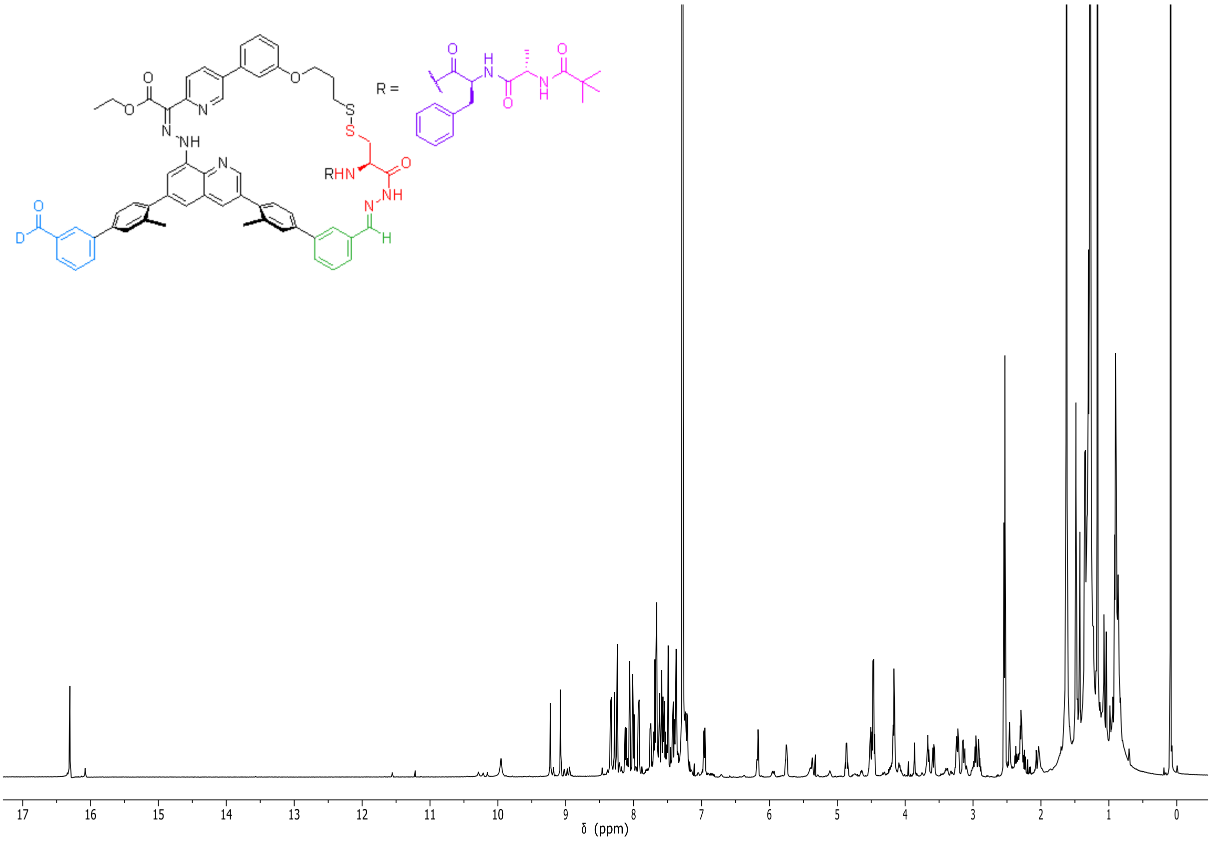

Spectrum 36. ^{1}H NMR (600 MHz, $CDCl_3$) of *E*-**11**

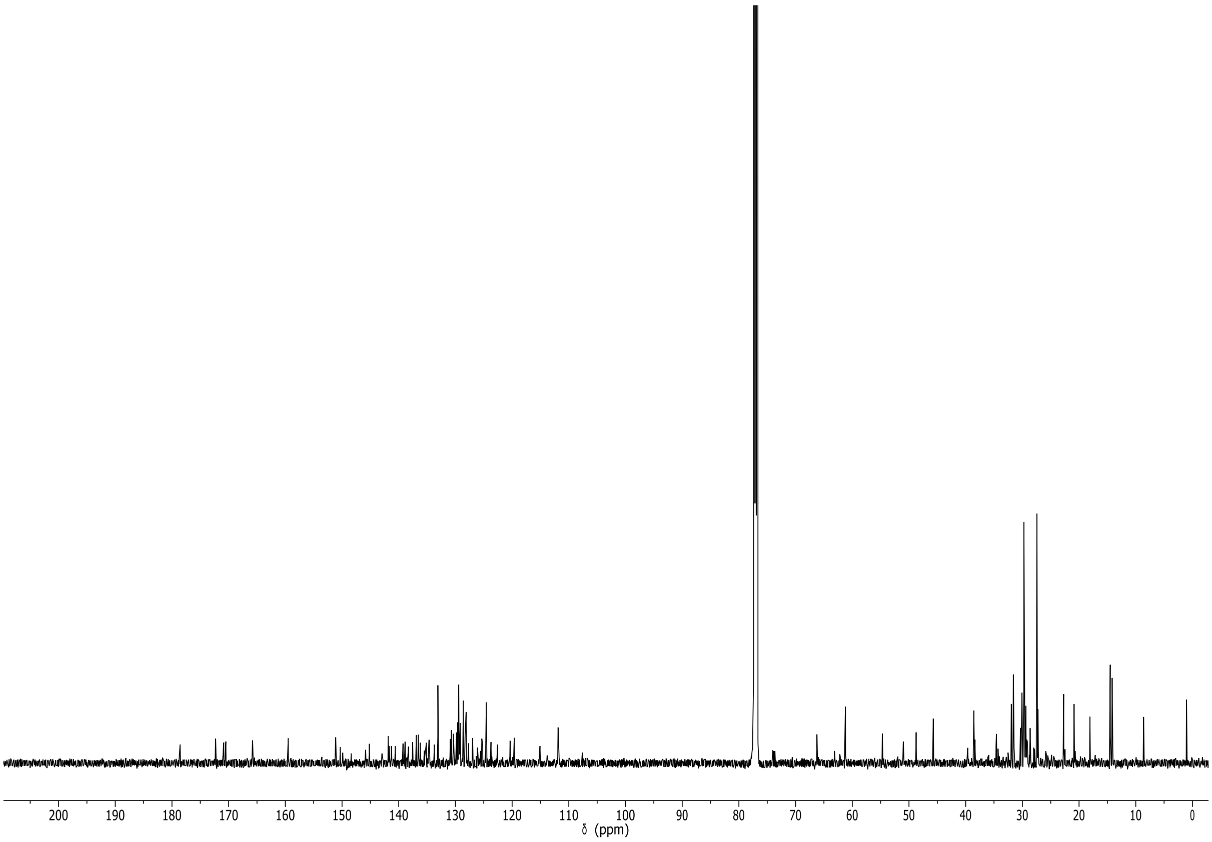

Spectrum 37. ^{13}C NMR (151 MHz, $CDCl_3$) of *E*-**11**

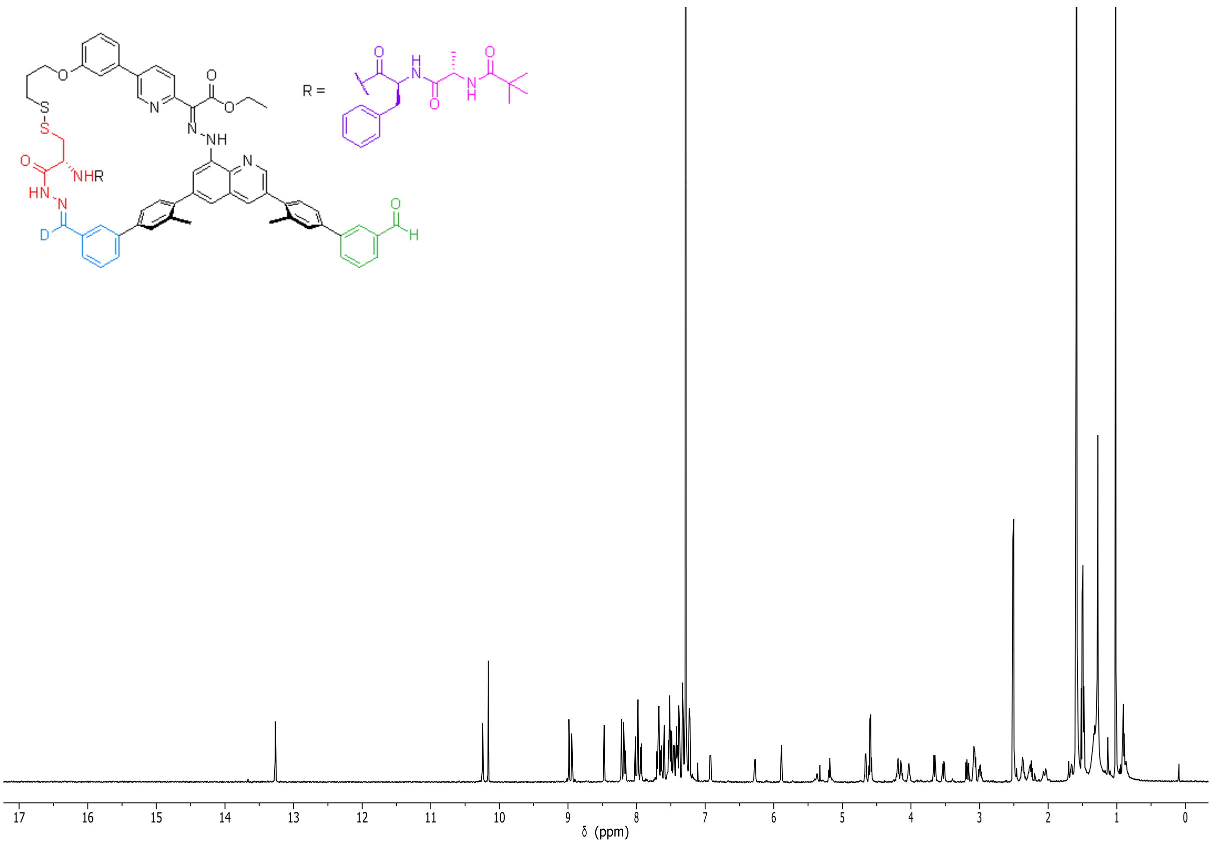

Spectrum 38. ^{1}H NMR (600 MHz, $CDCl_3$) of *Z*-**11**

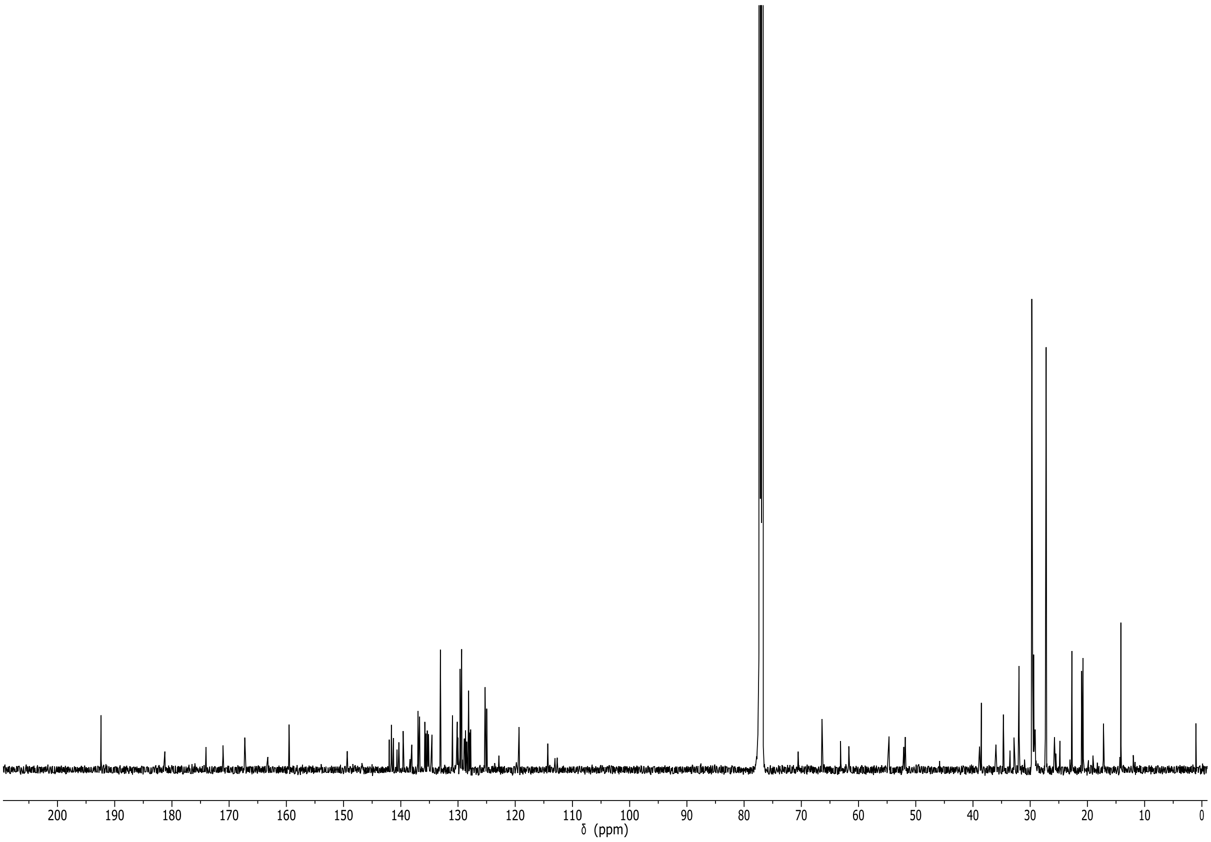

Spectrum 39. ^{13}C NMR (151 MHz, $CDCl_3$) of *Z*-**11**

8. References

[1] S. Kassem, A. T. L. Lee, A. Markevicius, D. A. Leigh, J. Solá, *Nat. Chem*. **2016**, *8*, 138.

[2] M. Kalek, G. C. Fu, *J. Am. Chem. Soc.* **2015**, *137*, 9438.

[3] H. E. Gottlieb, V. Kotlyar, A. Nudelman, *J. Org. Chem.* **1997**, *62*, 7512.

[4] S. Budavari, M. J. O'Neil, A. Smith, P. E. Heckelman, *The Merck Index, an Encyclopedia of Chemicals, Drugs, and Biologicals — Eleventh Edition*; Merck Co., Inc.: Rahway, NJ, **1989**.
